# Predicted Deep-Sea Coral Habitat Suitability for the U.S. West Coast

**DOI:** 10.1371/journal.pone.0093918

**Published:** 2014-04-23

**Authors:** John M. Guinotte, Andrew J. Davies

**Affiliations:** 1 Marine Conservation Institute, Seattle, Washington, United States of America; 2 School of Ocean Sciences, Bangor University, Menai Bridge, Anglesey, United Kingdom; College of Charleston, United States of America

## Abstract

Regional scale habitat suitability models provide finer scale resolution and more focused predictions of where organisms may occur. Previous modelling approaches have focused primarily on local and/or global scales, while regional scale models have been relatively few. In this study, regional scale predictive habitat models are presented for deep-sea corals for the U.S. West Coast (California, Oregon and Washington). Model results are intended to aid in future research or mapping efforts and to assess potential coral habitat suitability both within and outside existing bottom trawl closures (i.e. Essential Fish Habitat (EFH)) and identify suitable habitat within U.S. National Marine Sanctuaries (NMS). Deep-sea coral habitat suitability was modelled at 500 m×500 m spatial resolution using a range of physical, chemical and environmental variables known or thought to influence the distribution of deep-sea corals. Using a spatial partitioning cross-validation approach, maximum entropy models identified slope, temperature, salinity and depth as important predictors for most deep-sea coral taxa. Large areas of highly suitable deep-sea coral habitat were predicted both within and outside of existing bottom trawl closures and NMS boundaries. Predicted habitat suitability over regional scales are not currently able to identify coral areas with pin point accuracy and probably overpredict actual coral distribution due to model limitations and unincorporated variables (i.e. data on distribution of hard substrate) that are known to limit their distribution. Predicted habitat results should be used in conjunction with multibeam bathymetry, geological mapping and other tools to guide future research efforts to areas with the highest probability of harboring deep-sea corals. Field validation of predicted habitat is needed to quantify model accuracy, particularly in areas that have not been sampled.

## Introduction

Predictive habitat suitability modelling is a cost effective approach to assist scientific research, conservation and management of vulnerable marine ecosystems (VMEs) in the deep sea. To date, the majority of studies using predictive models in the deep sea have focused on deep-sea corals, mostly due to the conservation status of this group and the relative abundance of data compared to other VMEs (e.g. [1], [2]–[7]). These models identify areas with the highest probability of containing deep-sea corals and enhance our knowledge of the factors that control the distribution of these organisms. Whitmire and Clarke [8] reviewed the state of deep coral ecosystems in the waters of California, Oregon, and Washington and reported 101 species of corals from six cnidarian orders have been identified in the region. These included 18 species of stony corals (Class Anthozoa, Order Scleractinia) from seven families, seven species of black corals (Order Antipatharia) from three families, 36 species of gorgonians (Order Gorgonacea) from 10 families, eight species of true soft corals (Order Alcyonacea) from three families, 27 species of pennatulaceans (Order Pennatulacea) from eleven families, and five species of stylasterid corals (Class Hydrozoa, Order Anthoathecatae, Family Stylasteridae). The U.S. West Coast has been relatively well sampled for deep-sea corals in comparison to many other regions of the world's oceans, but the spatial distribution of deep-sea corals in unsurveyed areas within the EEZ remains largely unknown.

Predictive habitat models work by extrapolating potential species' distributions from presence data and a range of environmental variables. These two components are critical, as incomplete or erroneous data can reduce confidence in the approach and can potentially lead to predictions of limited conservation or management value [9]. When considering the utility of a model, one further consideration is the selection of an appropriate spatial scale. For example, poor spatial resolution of environmental data continues to hinder the spatial accuracy of deep-sea habitat modelling at global scales [3], [5], [7]. To address this, several studies have focused on improving smaller-scale, local models (i.e. 10 to 100 km2) by integrating terrain variables derived from multibeam bathymetry (e.g. [10], [11], [12]). Whilst smaller-scale modelling produces valuable data on species distributions in localised areas, it often requires intensive sampling effort and is of limited use in the identification of unknown habitat for cruise planning, management and conservation initiatives. Regional-scale models are needed to predict habitat suitability for corals in areas that have not been surveyed and have to be accurate enough to guide a research vessel towards a clearly defined area where sampling can be targeted [7]. Recent approaches have investigated the overlap between areas of protection and models of the distribution of vulnerable marine species [13], [14].

This manuscript presents a predictive habitat suitability modelling effort for deep-sea corals within U.S. Exclusive Economic Zone (EEZ) waters off the coasts of California, Oregon and Washington. The objectives of this manuscript are to; 1) develop predictive habitat suitability models at the highest possible spatial and taxonomic resolution for deep-sea corals, 2) use model results, in addition to other tools and data, to help guide field research efforts to areas with the highest probability of harboring deep-sea corals, and 3) integrate model results with existing bottom trawl closures (i.e. essential fish habitat (EFH) area closures) and National Marine Sanctuary boundaries to determine high probability habitat areas that remain at risk from human activity.

## Methods

### Coral presence data

Coral distribution data were gathered from several sources including: Monterey Bay Aquarium Research Institute (MBARI), NOAA Fisheries, NOAA National Marine Sanctuaries, Smithsonian Institute's National Museum of Natural History, and Washington State University. These records were obtained from a variety of gear types: remotely operated vehicles (ROVs), manned submersibles, cameras, grabs and bottom trawls. Over 90,000 coral records were collected for the U.S. West Coast region. However, only of a fraction of these could be retained for use in the habitat suitability models. Coral observations were eliminated if they matched the following criteria: 1) records were collected as bycatch in bottom trawls as they have inherent spatial and taxonomic accuracy issues, creating uncertainties that stem from both the method in which they were collected and the taxonomic knowledge of observers on fishing vessels. Bottom trawls can be several kilometers in length and it can be difficult, if not impossible, to determine the position of the actual coral occurrence [15]. 2) Records were located in waters of less than 50 m depth. This depth cutoff was based on the fact that most zooxanthellate corals are found in shallow waters and this study is focused on deep-sea azooxanthellate corals, which tend to occur in waters deeper than 50 m. 3) The taxonomy of coral records was uncertain at the family level. 4) If more than one coral record of the same taxon (order or suborder) was located within the same 500 m grid cell. The spatial resolution of the bathymetry, environmental data, and model results was 500 m×500 m. If more than one coral record from the same taxon occurred in the same 500 m grid cell, it was treated as a ‘spatial duplicate' and removed. Spatial duplicates skew models towards the environmental conditions found in those cells resulting in distorted model predictions. Some sampling approaches, such as ROVs, drop cameras or manned submersibles, document numerous coral records along relatively short distances and can introduce significant spatial bias into the analysis if all records are retained.

**Figure 1:**
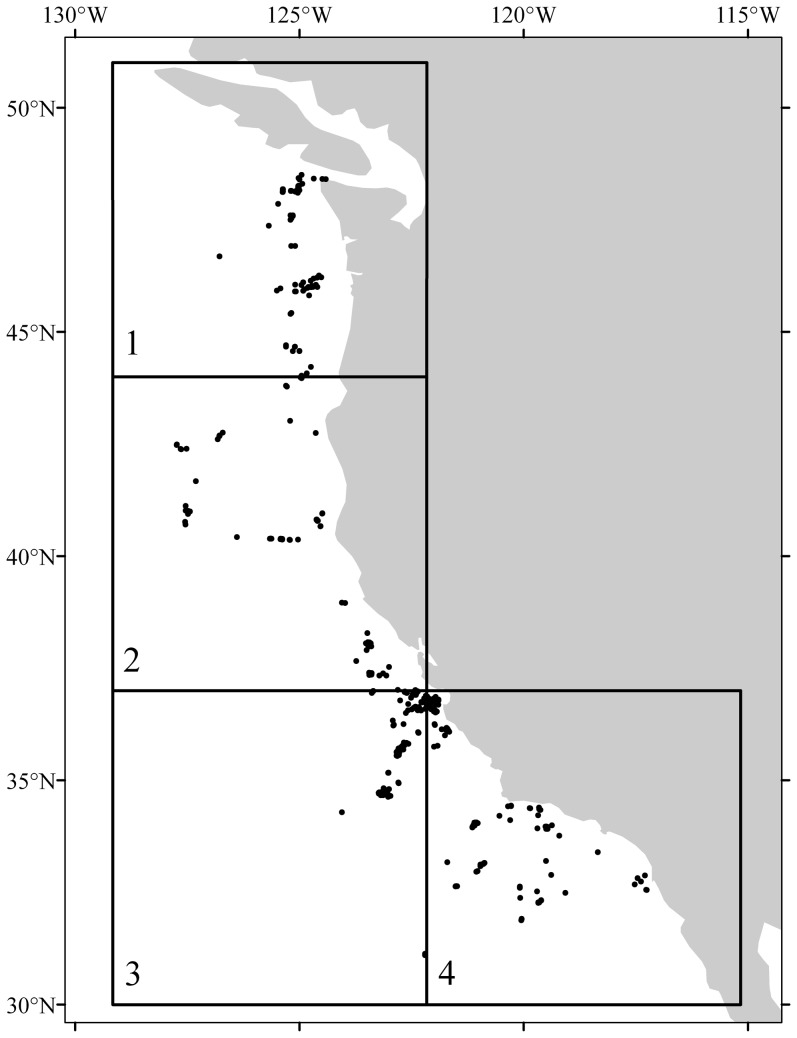
Location of all coral records (black markers) and regions 1–4 used for Maxent model cross-validation for each coral taxon.

There were several issues which prevented models from being performed at the species level: 1) taxonomic disagreement, 2) varying degrees of taxonomic knowledge among observers and collectors, and 3) many coral presences are documented without a sample being collected to conclusively determine coral taxonomy to species. These are concerns that have been similarly noted in global models for octocoral habitat suitability [16]. For these reasons coral records were binned and modelled at the Suborder and Order levels. Suborders for which coral presence data were obtained included Alcyoniina, Calcaxonia, Filifera, Holaxonia, Scleraxonia, Stolonifera. Order level data included Antipatharia and Scleractinia. A total of 2,120 coral records were retained for analysis (Table 1 and Figure 1). Predictive models were not performed for Suborders Filifera (n = 12) and Stolonifera (n = 30) due to a paucity of coral records. It should be noted that nine of the 203 scleractinian presence records used in the predictive models were habitat-forming scleractinians (e.g. *Lophelia pertusa* and *Oculina profunda*). All other scleractinian records were solitary, non-branching corals. Most scleractinian records used in this analysis were not structure forming, but habitat suitability was modelled due to the high level of research interest for this taxon.

**Table 1:** Coral records retained for habitat suitability modelling by taxon.

Taxa	Records retained
*Order*	
Antipatharia	128
Scleractinia	203
*Suborder*	
Alcyoniina	791
Calcaxonia	413
Holaxonia	308
Scleraxonia	277
*Total*	2120

Families of coral records listed included Order Antipatharia: Antipathidae, Cladopathidae, and Schizopathidae; Order Scleractinia: Fungiacyathidae, Micrabaciidae, Oculinidae, Caryophyllidae, Flabellidae, and Dendrophyllida; Suborder Alcyoniina: Alcyoniidae; Suborder Calcaxonia: Chrysogorgiidae, Isididae, and Primnoidae; Suborder Holaxonia: Acanthaogorgiidae, Gorgoniidae, and Plexauridae; Suborder Scleraxonia: Anthothelidae, Coralliidae, Paragorgiidae, and Plexauridae).

### Bathymetry

**Figure 2:**
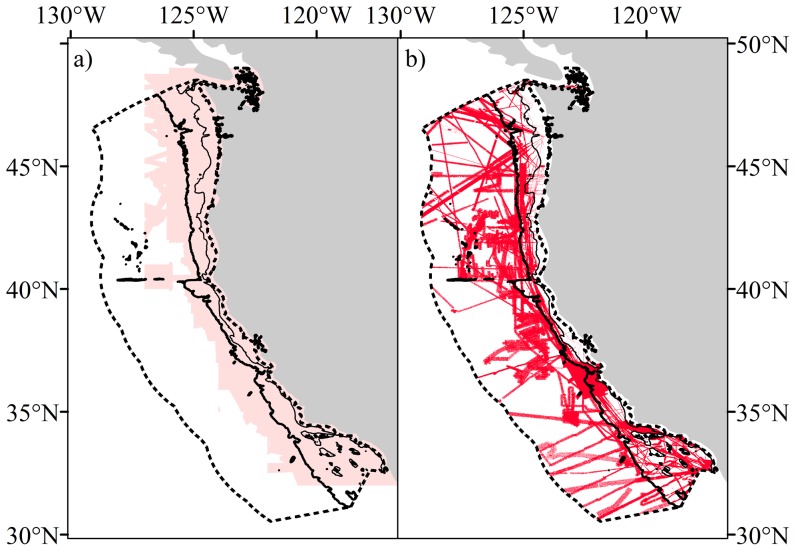
Custom bathymetry. a) Extent of the Coastal Relief Model data from NOAA (faint red) and the target model domain (dashed line). Faint black line indicates the 200 m contour, bold black line the 2000 m contour. b) Extent of soundings used to construct the custom 500 m×500 m bathymetry used in this study (red). Faint black line indicates the 200 m contour, bold black line the 2000 m contour and the dashed line indicates the analysis extent.

The bathymetry of the U.S. West Coast shelf consists of a complex series of canyons, ridges and seamounts [8]. There has been significant effort in the acquisition of reliable bathymetry in this region and several data products are available. The most prominent is the Coastal Relief Model (CRM) generated by NOAA's National Geophysical Data Centre (NGDC). This is a publically available dataset with a stated cell resolution of 3-arc seconds (∼90 m). The bathymetric component of the model is constructed from soundings obtained from National Oceanographic Service (NOS) hydrographic soundings, the NGDC multibeam database, and recently digitized soundings from NOS [17]. Soundings are gridded into a continuous grid for much of the shelf using the Generic Mapping Tools program Surface. However, the final CRM output is highly smoothed, omits smaller-scale features, and is of limited extent due to the high density of soundings in the shallower waters of the shelf (Figure 2a).

A custom bathymetry with a resolution of 500 m×500 m was produced from NOS hydrographic soundings, the NGDC multibeam database, and Trackline data [17]–[19]. Raw soundings were extracted into XYZ for the target area of interest (Figure 2b) using MB System [20]. As with the CRM dataset, the raw sounding data was not corrected to the same vertical and horizontal datum, but this has little effect on the accuracy of the final grid output [21]. Null values and erroneous soundings were removed using either a PERL script or were removed manually. The final grid was created using the spline interpolation program MB Grid. In total, 35% of the area of interest was covered by sounding data with additional data used to infill areas with sparse soundings from Smith and Sandwell's global seafloor topography version 14.1 [22]. The final resolution of the custom grid was 500 m×500 m; smaller cell sizes showed little improvement in the quality of the bathymetry as it is limited by the spatial coverage and density of soundings. The custom grid was highly correlated with CRM data (Pearson's correlation  = 0.999, p<0.001 based on 500 random points within the extent of CRM), spanned the entire study region, and retained more topographic complexity than CRM. However, considering this is the first development of a custom bathymetry, care should be taken when interpreting data in areas that contain sparsely distributed or no soundings as these areas rely on satellite derived altimetry for the bathymetry (Figure 2b).

### Environmental, physical and chemical data

Environmental layers were collated and constructed from sources that included ship-based CTD casts, satellites and global climatologies such as World Ocean Atlas (Table 2). The majority of source data was available as gridded datasets partitioned into standardized depth-bins ranging from 0 to 5500 m. Other data were available only as single layers from the surface (e.g. surface primary productivity) (Table 2). For depth-binned datasets, it was assumed that the conditions found at a specific gridded depth were representative of conditions on the seafloor. This allowed for the creation of continuous representations of seafloor conditions by extrapolating each depth-bin to the corresponding area of seafloor at that depth. This approach was initially developed for the creation of global environmental, physical, and chemical datasets [7].

**Table 2:** Environmental, physical, and chemical layers developed for this study.

Variable	Native resolution	Source
*Terrain variables*		
Depth^b^	0.0083°	Custom bathymetry
Slope^1^ ^, b^	0.0083°	Custom bathymetry
*Chemical variables*		
Apparent oxygen utilisation^b^, dissolved oxygen^b^, percent oxygen saturation^b^.	1°	Garcia et al. [51]
Aragonite^b^ and calcite^b^ saturation states	1°, 3.6°x0.8–1.8°	Orr et al. [52] ^2^, Steinacher et al. [53] ^3^
Nitrate^b^, phosphate^b^, silicate^b^	1°	Garcia et al. [54]
Salinity^b^, temperature^b^	0.25°	Boyer et al. [55]
*Biological variables*		
Particulate organic carbon^a^	0.08°	Lutz et al. [24]

1 Derived using ArcGIS spatial analyst and in several layers created using moving windows of 500 m, 1 km, 2.5 km, 5 km, 20 km. ^2^Extracted from OCMIP2 model data for 1995. ^3^Extracted from SRES B1 scenario model; mean 2000–2009. ^a^Indicates a surface variable. ^b^indicates a seafloor variable.

Converting depth-binned datasets into representations of seafloor conditions involved several computer intensive processes that were conducted within a series of Python scripts. Firstly, each depth-bin of the gridded data is extracted into a single layer and interpolated at a higher spatial resolution (usually 0.1°) using inverse distance weighting. The interpolation was required to reduce gaps that appear between adjacent depth bins due to a lack of overlap when extrapolated to the bathymetry. Each of these layers was then resampled to match the extent and resolution of the bathymetry with no further interpolation. Secondly, these layers were resampled to match the extent and cell resolution of the bathymetry. Thirdly, each resampled depth-bin was clipped by the area of seafloor that was available at that particular depth. Each bin did not overlap and all were merged to produce a continuous representation of the variable on the seafloor.

This approach essentially develops a model of potential conditions for each variable. It uses the best available data, but makes several assumptions: 1) environmental conditions from the gridded CTD data are representative of the conditions at the seafloor. The majority of CTD casts do not normally reach the seafloor as they are usually stopped between 5 and 10 m from the bottom to reduce the chance of damage to the CTD system through impact, and 2) seafloor conditions are relatively stable. Annual mean values were used to maximize the amount of environmental data incorporated. While much of the deep sea is relatively stable below 200 m, there is still significant temporal variability in shelf and coastal areas and caution should be taken when interpreting predictions in shallow-water areas. However, the longevity of many deep-sea coral species far exceeds the measuring period of most oceanographic variables. Surface datasets were not up-scaled by the above process, as they were only available as a single depth-bin. Surface variables were interpolated to a higher spatial resolution using the data-interpolating variational analysis approach (DIVA; [23]) that is written into Ocean Data View version 4. For this study, we selected particulate organic carbon flux to the seafloor from Lutz et al. [24] as the productivity variable. Slope was calculated within ArcGIS Spatial Analyst using a moving window to extrapolate both fine scale slopes (1 km, 5 km) and broad-scale slopes (10 km, 20 km) using Horn's algorithm [25].

**Figure 3:**
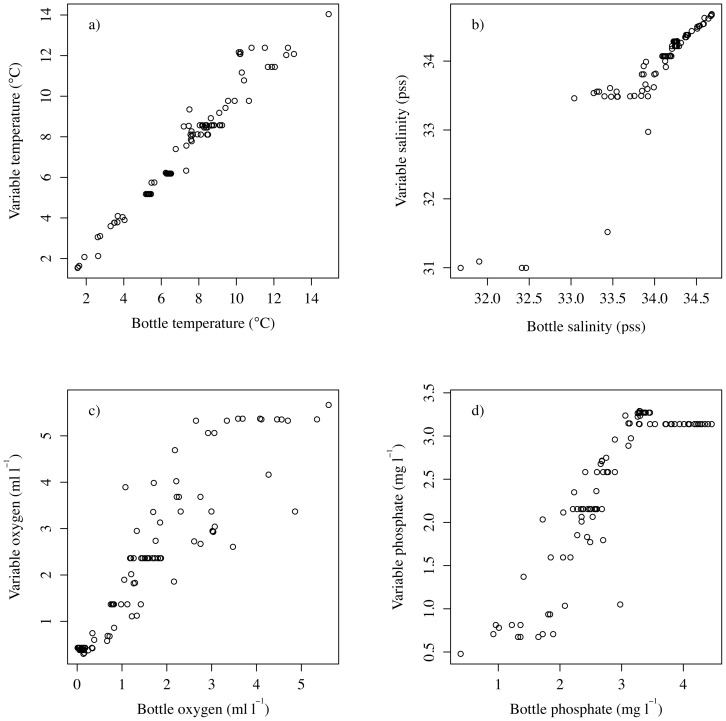
Validation of the environmental layer creation process by comparing the variable value on y axis (Variable), against WOD 2009 CTD data (Bottle, on × axis). a) Temperature, b) salinity, c) dissolved oxygen and d) phosphate.

The accuracy of the up-scaled environmental variables was tested using quality controlled water bottle data obtained from the 2009 version of the World Ocean Database (WOD) [26]. Only points collected post-2001 were used in the statistical comparison and null values were removed as these were not used in the development of the temperature or salinity grids. WOD data were filtered to ensure 1) values contained a bottom depth meta-data flag, and 2) data values were within 5% of the total depth from the custom bathymetry for a cast location. Four variables contained adequate data for statistical comparison with environmental layers: temperature, salinity, phosphate and dissolved oxygen (Figure 3). The four up-scaled environmental variables that were assessed with WOD water bottle data were highly correlated at each sampling location (Pearson's correlation, R2, temperature  = 0.98 (*n* = 108), salinity  = 0.93 (*n* = 105), dissolved oxygen  = 0.91 (*n* = 100) and phosphate  = 0.88 (*n* = 101), all values significant at *p*<0.001). The phosphate comparison showed an artifact at bottle concentrations above 3.5 mg l-1 (Figure 3). This occurred in regions that have low topographic relief, resulting in environmental variables not being upscaled by the bathymetry and the original resolution of the environmental variable being visible (in this case 1°). Several other CTD datasets from the U.S. West Coast were assessed for suitability, but many did not penetrate into the deep sea and did not include bottom depth as meta-data making it impossible to determine whether a cast went near the seafloor.

### Variable selection

Variables were selected based on a literature search of environmental, physical, and chemical factors known or thought to influence deep-sea coral growth and survival. Temperature, salinity, aragonite saturation state, and topographic complexity have been shown to be strong predictors of bioherm forming scleractinian coral distribution in recent deep-sea modelling efforts [3], [5], [7], [27]. Calcite saturation state was chosen over aragonite saturation state for use in this study as the majority of coral taxa that were modelled use calcite to build their calcium carbonate spicules and structures [16]. Scleractinians have aragonitic skeletons, but the vast majority of scleractinian corals used in this analysis were solitary, non-reef forming species. Living specimens of these solitary species have been collected in highly undersaturated waters with respect to aragonite, which led to the deduction that aragonite saturation state would not be a strong predictor for determining their potential distribution at a regional scale [28]. Slope was calculated at a variety of spatial resolutions ranging from 1 km–20 km and is a useful proxy for current acceleration and mixing, which are known to influence coral distribution and abundance [29]–[31].

Covariation between environmental datasets is a complication that must be addressed in many predictive modelling efforts. Environmental datasets used in this analysis were assessed for covariation in correlation matrices (See Figures S1–S10). Although Maxent is reasonably robust with respect to covariation, an *a priori* variable selection process was used to reduce covariation by removing variables that were highly correlated and likely to adversely affect final predictions. Covariation was assessed using correlation matrices in R. Strong correlations between variables (> 0.7) were addressed by omitting one of the environmental variables (except for calcite saturation state, temperature, and depth; see Results and Discussion). The importance of each variable in the model was assessed using a jack-knifing procedure that compared the contribution of each variable (when absent from the model) with a second model that included the variable. The final habitat suitability maps were produced by applying the calculated models to all cells in the study region, using a logistic link function to yield a habitat suitability index (HSI) between zero and one [32].

### Modelling Methods

Maxent version 3.2.1 (http://www.cs.princeton.edu/~schapire/maxent) was used to model predicted deep-sea coral distributions for the U.S. West Coast. Maxent (maximum entropy modelling) consistently outperforms other presence-only modelling packages including Ecological Niche Factor Analysis (ENFA) [5], [33]. Presence-only modelling is one of the only methods available for modelling species distributions in the deep sea because documented absence data is sparse and when available can be unreliable. Maxent's underlying assumption is the best way to determine an unknown probability distribution is to maximise entropy based on constraints derived from environmental variables [32]. Default model parameters were used as they have performed well in other studies (a convergent threshold of 10^−5^, maximum iteration value of 500 and a regularisation multiplier of 1 [34]).

Model accuracy between the test data and the predicted suitability models was assessed using a threshold-independent procedure that used a receiver operating characteristic curve with area under curve (AUC) for the test localities and a threshold-dependent procedure that assessed misclassification rate. With presence-only data, Phillips et al. [32] define the AUC statistic as the probability that a presence site is ranked above a random background site. In this situation, AUC scores of 0.5 indicate that the discrimination of the model is no better than random, with the maximum achievable AUC value being 1, which implies perfect discrimination of validation data. To develop the models in this study, coral presence data was spatially partitioned into four regions to calculate validation metrics and assess whether or not spatial sampling bias of coral records was influencing model performance (regions depicted in Figure 1). Four Maxent models were performed for each coral taxon (order/suborder) so models could be spatially cross-validated. For example, model 1 for any given taxon used coral records from regions 2, 3, and 4 as training records and region 1 coral records as test data to assess model performance using AUC. Model 2 used coral records from regions 1, 3, and 4 as training records and region 2 coral records as test records. The same procedure was performed for models 3 and 4. The cross-validation of models across the four regions was necessary due to the high number of coral presence records provided by MBARI for Monterrey Canyon and Davidson seamount and has the benefit of testing models with spatially independent data (see regions 3 and 4 in Figure 1). In this study, spatially cross-validated models with AUC scores >0.7 were retained for further analysis and used in the production of thresholded predictions, models scoring lower than this were omitted.

There is ongoing debate regarding the interpretation of Maxent's logistic prediction values (0–1) for habitat suitability [35], [36], but it represents the best metric at present. Several studies have defined a binary threshold, which states that a species is likely to be found in an area with a habitat suitability value above a given threshold, but not likely to be found below it [37]–[39]. To create usable predictions from the cross-validations we used a 0.5 logistic presence value threshold for each taxa and all taxa to provide a cut-off point for suitability in this study (below which was considered unsuitable and above suitable), we also used a 0.75 logistic presence value threshold to further constrain the model output for EFH management applications. These values are higher than used in previous studies (i.e. the 10^th^ percentile training presence used in [7]) because the main application of this modelling effort is to use predictions to help target areas for future field research and provide an assessment of EFH area closures. Summary maps were generated for each order and suborder by creating thresholded predicted outputs for each of the four regions (using 0.5 or 0.75 as the cutoff presence/absence value). If predicted logistic suitability was greater than 0.5 or 0.75 for any given cell, that cell was assigned a value of 1. If predicted habitat suitability was less than the cutoff value, the cell was assigned a value of 0. The binary models were summed for each coral taxon resulting in final consensus grids that had cell values ranging 0–4 depending on the number of retained spatially-partitioned models (maximum  = 4).

### Fishing intensity data

To estimate the amount of fishing activity within the U.S. West Coast, a map of fishing intensity was acquired that showed the relative intensity of commercial bottom trawling from 12 June 2006 to 31 December 2010 [40]. The map was developed from a commercial logbook program administered by coastal states and records for bottom-contact fishing gear (e.g., “small” footrope, “large” footrope, flatfish, selective flatfish, and roller trawl) collated by the Pacific Fisheries Information Network (PacFIN). This is not a fully comprehensive dataset, as some states do not submit full data, state-managed fisheries such as pink shrimp, ridgeback prawn and sea urchin are not included in PacFIN and cells with data from less than three fishing vessels were omitted from available maps to protect privacy. The final layer represents the total bottom trawl lines that fall within a 3 km radius neighbourhood centered on cells within a 500×500 m grid similar to the custom bathymetry (represented as km of trawl per km^2^). These data were contrasted against a thresholded prediction for all taxa, with the threshold raised to 0.75 to locate areas that are highly suitable habitat for cold-water corals.

### Substrate data

Substrate data was obtained for a limited subset of the model domain that mostly covered the shallower shelf of the US West Coast [41]. This layer was built from a variety of archived data including limited multibeam echosounder bathymetry and backscatter and was provided as a 25 m×25 m grid [42]. The substrate types described included, probable soft sediment, probable rock (including predicted rock based on expert knowledge) and a mixture of soft sediment and rock. This layer was also accompanied with a confidence layer that shows that for the majority of the data, the probable substrate type was of low confidence with only shallower water being granted medium and high confidence levels. This data layer and any output from it must be considered with substantial caution especially given that a wide variety of mapping approaches were used in its creation and these sources were interpreted into the three coarse categories. Due to the low confidence in the substrate data, an exploratory analysis was undertaken to determine how the habitat suitability models for each species fared in light of the available substrate in the region.

## Results

### Species' niche

From the available environmental data, an *a priori* variable selection process that took into account closely related and highly correlated variables, identified seven variables that were likely to influence the probability of species presence (temperature, salinity, particulate organic carbon, depth, dissolved oxygen, calcite saturation state, slope 1 km) (Tables 3, 4 and S1–S10 in File S1). The jack-knife of variable contribution showed slope 1 km, temperature, and salinity were the strongest predictors for Suborder Alcyoniina, Order Antipatharia, Suborder Calcaxonia, and Suborder Scleraxonia. Temperature and salinity were consistently strong predictors in models for Suborder Holaxonia, whereas, salinity and depth were the strongest predictors for Order Scleractinia. For all taxa combined the strongest predictors were salinity, temperature and depth. Three highly correlated variables (depth, calcite saturation state, and temperature) were retained due to ecophysiological importance and the strength of their contributions. This must be interpreted with caution as these layers covary and may contain similar information, which can artificially inflate variable contribution scores. However, the test AUC scores for models generated with a single variable reinforced that these variables were top predictor variables regardless of covariation (Tables 3 and 4). Suborder Holaxonia was the only group to have calcite saturation state as one of the top three predictor variables (two of the four models) indicating some species within this Suborder could be sensitive to changes in carbonate chemistry.

**Table 3:** Validation statistics and jack-knife analysis of variable contributions to the models for all taxa (50^th^ percentile), Alcyoniina, Antipatharia and Calcaxonia.

	All Taxa				Alcyoniina				Antipatharia				Calcaxonia			
Cross-validation cell	1	2	3	4	1	2	3	4	1*	2	3	4	1	2	3	4
*Validation statistics*																
Test AUC	0.82	0.846	0.815	0.943	0.871	0.861	0.881	0.95	0.577	0.835	0.929	0.912	0.787	0.923	0.922	0.896
Test AUC standard deviation	0.016	0.012	0.016	0.005	0.026	0.015	0.013	0.004	0.204	0.033	0.017	0.037	0.036	0.016	0.01	0.011
10th percentile training presence	0.4866	0.5028	0.4545	0.4222	0.515	0.577	0.4962	0.4586	0.434	0.4829	0.2917	0.4321	0.6061	0.5946	0.4228	0.4631
Maximum test sensitivity plus specificity	0.181	0.262	0.412	0.379	0.153	0.162	0.098	0.35	0.57	0.054	0.375	0.26	0.046	0.195	0.117	0.323
*Jack-knife of variable importance (jack of regularized training gain)*																
Depth	**0.7343**	0.8258	**1.0306**	**0.6101**	0.9197	1.0217	**1.2143**	0.7726	1.0188	1.1635	0.528	1.0441	1.1326	1.1511	1.3354	1.1089
Dissolved Oxygen	0.5784	0.5817	0.6399	0.5601	0.8359	0.8946	0.9726	**0.9285**	1.2431	1.3786	0.4208	**1.4051**	1.0852	1.0692	1.1143	**1.3166**
Calcite Saturation State	0.6823	0.7536	0.9572	0.5682	0.9203	1.0206	1.1697	0.8571	1.2913	1.4405	**0.5438**	1.3341	1.1359	1.1417	1.2385	1.2377
Particulate Organic Carbon	0.6244	0.7207	0.9181	0.5533	0.7423	0.8689	1.0206	0.6541	0.798	0.7914	0.2422	1.0263	0.5591	0.55	0.9316	0.7141
Salinity	0.815	**0.8898**	**1.0605**	**0.6733**	**0.9825**	**1.1053**	**1.2755**	0.8786	1.2911	**1.4415**	0.5035	**1.3727**	**1.2273**	**1.2426**	**1.3415**	1.296
Slope (1 km)	**0.9349**	**0.9133**	0.8904	0.5992	**1.2309**	**1.3442**	**1.3521**	**1.0203**	1.4655	**1.5807**	**1.3852**	1.3643	**1.5481**	**1.4591**	**1.4562**	**1.3894**
Temperature	**0.749**	**0.8451**	**1.0054**	**0.6698**	**0.9414**	**1.0662**	1.1859	**0.8904**	1.4282	**1.627**	**0.7199**	**1.4986**	**1.2905**	**1.2951**	**1.3924**	**1.3747**
*Test AUC for a single variable*																
Depth	**0.9045**	0.7761	0.7048	**0.8734**	**0.9403**	**0.8161**	0.784	0.8639	0.6869	0.6496	0.8357	0.7562	**0.8125**	0.8368	0.8067	**0.8675**
Dissolved Oxygen	0.7588	**0.8111**	**0.7253**	0.7746	0.8504	0.8013	**0.7904**	0.8379	0.5127	**0.7592**	0.7807	0.6544	0.7517	**0.853**	0.7973	0.7244
Calcite Saturation State	**0.8791**	0.7738	**0.7312**	0.8449	**0.9165**	**0.8303**	**0.8193**	0.845	0.5853	0.6969	**0.8488**	**0.8689**	**0.8115**	0.8512	**0.8235**	0.8435
Particulate Organic Carbon	0.8558	0.7159	0.6523	0.7759	0.7956	0.7	0.673	0.6805	0.611	0.771	0.6574	0.5644	0.693	0.6625	0.6173	0.6079
Salinity	0.8185	**0.8036**	0.7082	0.8618	0.9113	0.7971	0.7875	0.8921	0.6308	**0.8093**	0.8468	0.5811	0.7874	**0.8524**	**0.8133**	0.8549
Slope (1 km)	0.6041	**0.7819**	**0.8298**	**0.9205**	0.7739	**0.8421**	**0.8948**	**0.9515**	0.7553	**0.7945**	**0.9116**	**0.9571**	0.727	**0.9184**	**0.9324**	**0.9436**
Temperature	**0.8882**	0.7622	0.6842	**0.8656**	**0.9331**	0.7594	0.7889	0.8476	0.6478	0.6091	**0.8535**	**0.7822**	**0.8095**	0.8448	0.8045	**0.8603**

Higher values for the regularized training gain of the jack-knife test indicates greater contribution to the model for a variable (these values are not directly comparable between the different taxa). Test AUC numbers in parentheses are the standard deviation of the Test AUC scores. The top three variables are highlighted in bold for each taxon, both for the jack-knife variable contribution and test AUC values for Maxent models generated using a single variable. *indicates cross-validation cells that were eliminated due to low Test AUC scores.

**Table 4:** Validation statistics and jack-knife analysis of variable contributions to the models for Holaxonia, Scleractinia and Scleraxonia.

	Holaxonia				Scleractinia				Scleraxonia			
Cross-validation cell	1	2	3	4	1	2	3*	4	1	2	3	4
*Validation statistics*												
Test AUC	0.854	0.898	0.851	0.934	0.926	0.95	0.396	0.959	0.824	0.957	0.942	0.895
Test AUC standard deviation	0.011	0.018	0.011	0.006	0.014	0.006	0.059	0.009	0.021	0.01	0.009	0.01
10th percentile training presence	0.4679	0.3864	0.3992	0.4575	0.544	0.4343	0.4062	0.483	0.6404	0.556	0.4058	0.5849
Maximum test sensitivity plus specificity	0.05	0.17	0.031	0.174	0.122	0.338	0.515	0.087	0.019	0.244	0.004	0.092
*Jack-knife of variable importance (jack of regularized training gain)*												
Depth	1.2116	**1.244**	**1.3494**	1.1917	**1.1782**	**1.1851**	1.5965	**1.1525**	1.4298	1.3367	**1.6165**	1.3282
Dissolved Oxygen	0.8588	0.7853	0.7656	0.7875	0.3822	0.3561	0.4062	0.6344	1.4176	1.2455	1.3594	1.4416
Calcite Saturation State	**1.192**	1.2233	1.2653	**1.2093**	0.9057	0.889	1.4655	0.7286	1.4166	1.3256	1.4452	**1.5113**
Particulate Organic Carbon	0.9251	1.0044	**1.3606**	0.8145	1.1713	**1.1957**	1.5983	0.9781	0.7525	0.7048	1.1665	0.8166
Salinity	**1.26**	**1.3094**	**1.3832**	**1.297**	**1.3867**	**1.387**	1.7	**1.3008**	**1.5009**	**1.395**	**1.5007**	**1.5334**
Slope (1 km)	1.1797	0.9897	0.7825	0.5465	0.6968	0.8238	0.932	0.1163	**1.6012**	**1.416**	1.3632	1.1158
Temperature	**1.2693**	**1.3011**	1.328	**1.3376**	**1.2203**	1.1763	1.5474	**1.2191**	**1.5832**	**1.5469**	**1.7183**	**1.5909**
*Test AUC for a single variable*												
Depth	**0.8897**	**0.8617**	0.7838	**0.8747**	**0.9606**	0.9258	0.4169	**0.898**	**0.8057**	**0.9097**	0.8256	0.8735
Dissolved Oxygen	0.758	0.8098	**0.8116**	0.8161	0.6083	0.8045	0.3762	0.5607	0.6921	**0.9164**	**0.8485**	0.7649
Calcite Saturation State	**0.8652**	0.8545	0.7811	0.8398	**0.9398**	**0.9279**	0.3778	0.8443	**0.7799**	0.91	**0.8437**	0.8363
Particulate Organic Carbon	0.8273	0.7954	0.597	0.8424	0.9024	0.8826	0.5526	0.8698	0.6085	0.6775	0.4183	0.6408
Salinity	0.8436	**0.8631**	**0.7988**	0.8686	**0.9441**	**0.9398**	0.4193	**0.9038**	0.7735	0.8932	0.8038	0.8598
Slope (1 km)	0.5424	0.7232	**0.927**	**0.9077**	0.7569	0.4317	0.4468	0.7678	0.6157	0.8681	**0.9331**	0.9469
Temperature	**0.8742**	**0.8641**	0.7633	**0.8897**	0.905	**0.9402**	0.3001	**0.9024**	**0.869**	**0.9115**	0.8395	0.8912

Higher values for the regularized training gain of the jack-knife test indicates greater contribution to the model for a variable (these values are not directly comparable between the different taxa). Test AUC numbers in parentheses are the standard deviation of the Test AUC scores. The top three variables are highlighted in bold for each taxon, both for the jack-knife variable contribution and test AUC values for Maxent models generated using a single variable. *indicates cross-validation cells that were eliminated due to low Test AUC scores.

**Figure 4:**
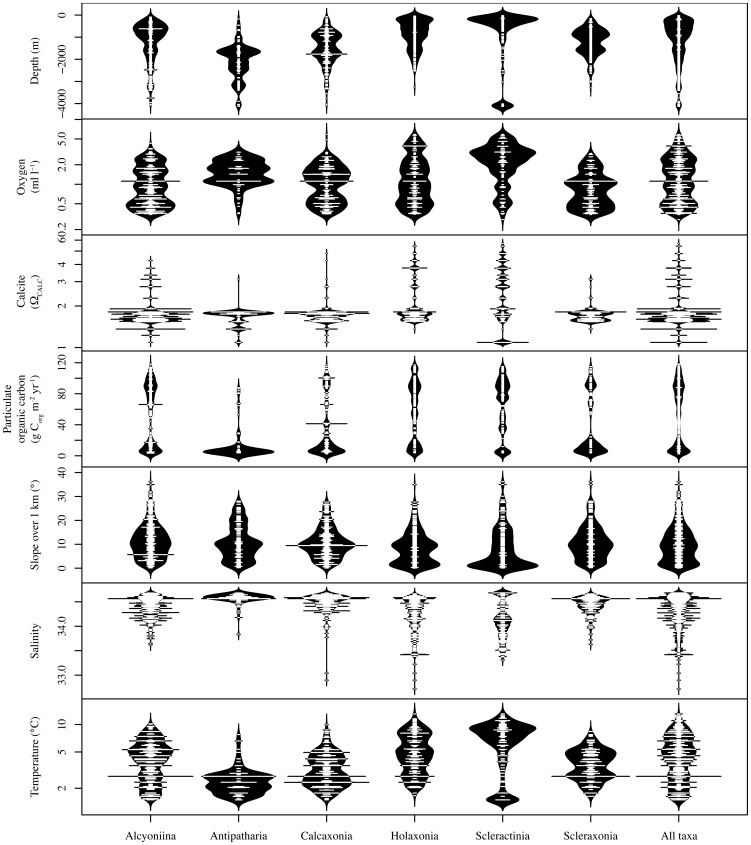
Bean plots of coral presences intersected with the environmental, physical and chemical variables used in the models (the small lines in the center of each bean shows individual coral presence points). The bean itself is a density trace that is mirrored to show as a full bean [56].

It was possible to gain insight into the species niches and the factors that are most important in driving their distribution by intersecting the distribution of coral records with the environmental, physical, and chemical layers (Figure 4). For Ω_CALC_, all coral records were found in waters supersaturated with respect to calcite (Ω_CALC_ >1) with the majority (82%) being found in waters with Ω_CALC_ between 1 and 2. Most coral records were found in waters with a temperature range of 1.5–8°C and salinity in the range of 33.5–34.7. Coral records were found in depths ranging from 50–4,129 m, but the majority (88%) were found between 50 and 2500 m. Slope 1 km values were widely distributed across taxa, but was an important predictor variable (see jack-knife of variable importance in Tables 3 and 4) for all taxa combined, Alcyoniina, Antipatharia, Calcaxonia, and Scleraxonia. Slope 1 km was not in the top three predictor variables for Holaxonia and Scleractinia. Dissolved oxygen values ranged from 0.3–5.9 ml l-1 with 89% of records having values in the 0.3–3.1 ml l-1 range. Particulate organic carbon values were widely distributed across taxa with Antipatharia having notably lower POC values (80% of Antipatharian records were found in waters with POC<7 g Corg m-2 yr-1).

## Model evaluation

**Figure 5:**
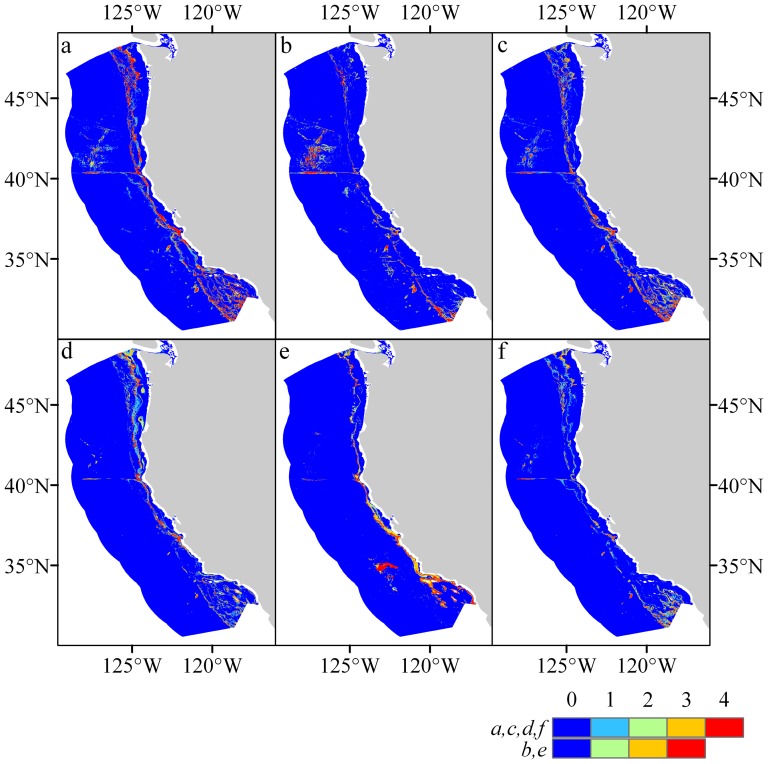
Predicted habitat suitability. Suborder Alcyoniina (a), Order Antipatharia (b), Suborder Calcaxonia (c), Suborder Holaxonia (d), Order Scleractinia (e), Suborder Scleraxonia (f). Legend shows the summed values for 0.5 cut off for each model validated by the spatial cross validation approach, the legend is on a scale of 0–4 for most variables, however, some only had 3 valid cells incorporated and the summed value is a maximum of 3.

**Figure 6:**
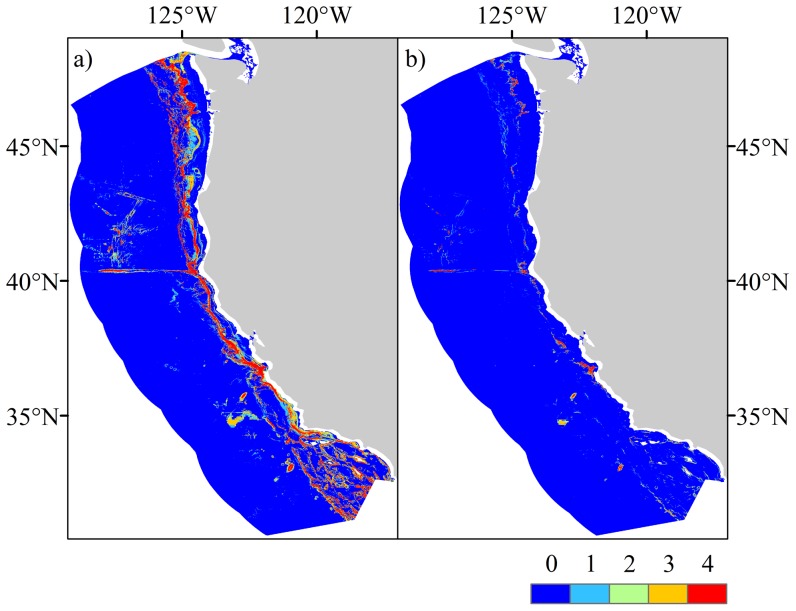
Predicted habitat suitability for all taxa. a) 0.5 threshold as per the previous figures, b) 0.75 threshold. The higher threshold greatly constrains the output, producing predictions that are more focused on areas of the highest suitability.

The coral habitat models performed well across all the metrics used to validate the modeled outputs. All, bar two AUC scores, were >0.7 and were significantly different from that of a random prediction of AUC = 0.5 (Wilcoxon rank-sum test, p<0.01). High AUC scores were supported by high test gains and low omission rates across many of the modeled taxa indicating most presences were accounted for in the predictions (Tables 3 and 4). Models generated for the spatial partitions of Antipatharia (Model 1) and Scleractinia (Model 4) were excluded from the summed grids for each taxon as the AUC scores for these partitions were <0.7, so suitability was ranked between 0–3, rather than 0–4 as for all other taxa (Figures 5 and 6).

## Habitat suitability maps for National Marine Sanctuaries

**Figure 7:**
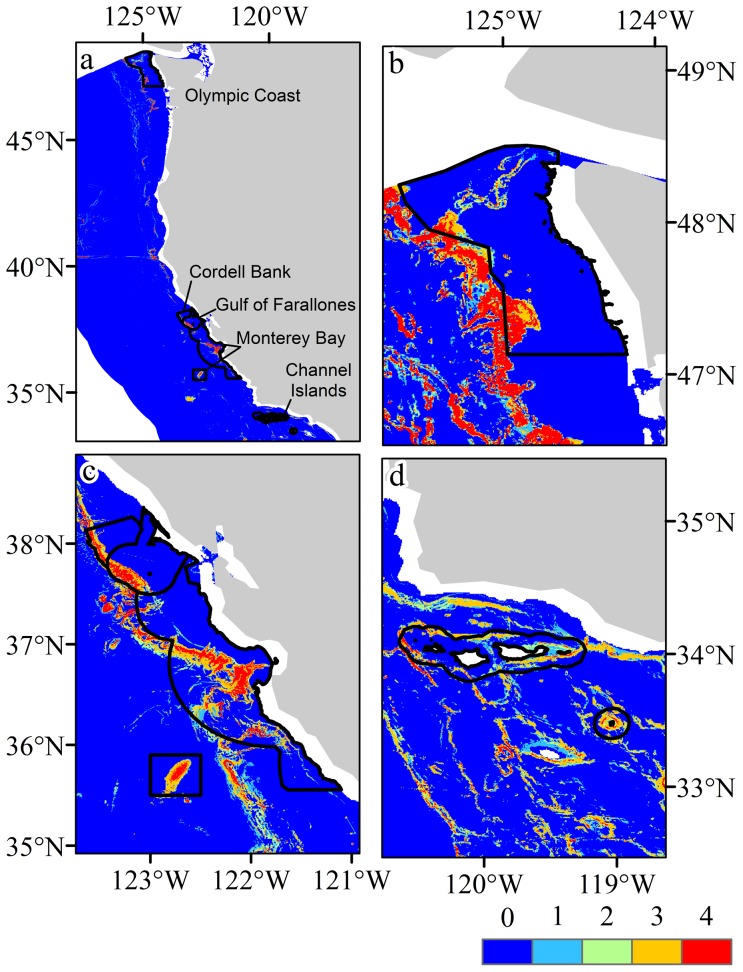
Locations of National Marine Sanctuaries overlain with predicted suitability. a) Overview with all taxa constrained to a 0.75 threshold, b) Olympic Coast and prediction for Alcyoniina, c) Cordell Bank, Gulf of the Farallones and Monterey Bay with prediction for Calcaxonia, d) Channel Islands and prediction for Holaxonia.

Habitat suitability maps for each taxon and all taxa combined are presented in Figures 5 and 6 and are available to download as GeoTIFF files (File S2). The locations of the Olympic Coast, Cordell Bank, Gulf of the Farallones, Monterey Bay and Channel Islands National Marine Sanctuaries overlain with predicted suitability for selected taxa are shown in Figure 7, additional and higher resolution digital figures are available as electronic supplementary materials (Figures S1–S7).

Olympic Coast National Marine Sanctuary (OCNMS): Areas within the OCNMS that have the highest probability of containing coral habitat include the eastern regions of Juan de Fuca Canyon, Quillayute Canyon, and Quinalt Canyon. Habitat suitability probabilities were highest in these areas for Alcyoniina (Figure 7b and S1a), Calcaxonia (Figure S1c), Holaxonia (Figure S1d), Scleractinia (Figure S1e), and Scleraxonia (Figure S1f). Predicted habitat suitability and the areal extent of suitable habitat were low in these areas for Antipatharia (Figure S1b). Large areas of suitable deep-sea coral habitat were predicted in areas adjacent to the western boundary of the OCNMS. This area of high habitat suitability extends approximately 30 km westward of the sanctuary's western boundary (Figure S1).

Cordell Bank (CBNMS), Gulf of the Farallones (GFNMS), and Monterey Bay National Marine Sanctuaries (MBNMS): The boundaries of these sanctuaries encompass the vast majority of suitable coral habitat in the region from the coastline westward to approximately 90 km offshore (from Monterey Bay). Habitat suitability probabilities and areal extent of predicted habitat were highest in these sanctuaries for Alcyoniina (Figure S2a), Calcaxonia (Figure 7c and S2c), Holaxonia (Figure S2d), and Scleractinia (Figure S2e). In contrast, suitability probabilities and areal extent were low in these areas for Antipatharia (Figure S2b) and Scleraxonia (Figure S2f). High probability areas were modeled to the west of MBNMS's northwest boundary. This area of highly suitable habitat extends approximately 50 km to the west of MBNMS's NW boundary (Figure S2).

Channel Islands National Marine Sanctuary (CINMS): Habitat suitability probabilities and areal extent of predicted habitat were generally low across taxa within the CINMS boundary when compared to surrounding waters. Predicted habitat probabilities and areal extent of suitable habitat were low for Alcyoniina (Figure S3a), Antipatharia (Figure S3b), Calcaxonia (Figure S3c), Holaxonia (Figure 7d and Figure S3d), and Scleraxonia (Figure S3f). Modeled habitat probabilities and areal extent of predicted habitat were high in waters surrounding all islands within the sanctuary for Scleractinia (Figure S3e). It is worth noting that probabilities and areal extent were high in the waters surrounding the islands of San Nicolas, Santa Catalina and San Clemente for Alcyoniina, Calcaxonia, Holaxonia, Scleractinia and to a lesser extent Scleraxonia. Probabilities and areal extent were very low in all island waters, both within and outside the sanctuary boundaries, for Antipatharia (Figure S3).

To evaluate the effectiveness of each NMS in encompassing predicted coral habitat, the total area of suitable habitat within each was calculated (Table 5). Overall, a large proportion of total area within each sanctuary encompassed habitat that was classed as suitable (a habitat suitability value greater than zero) for multiple taxa. In some NMS', certain taxa dominated. For example, suitable habitat for Holaxonia was predicted to occur within 45% of the area of OCNMS, whereas in the Gulf of Farallones, Antipatharia was predicted to occur in 60% of the area. To avoid over-prediction skewing the effectiveness, the highly constrained model for all taxa (cut-off above 0.75 logistic suitability) showed that the most effective NMS may be the Gulf of Farallones, which contained 16% suitable habitat, followed by Monterey Bay that contained 14%, Olympic Coast at 12% and the Channel Islands at 9%. The least effective may be Cordell Bank that encompassed 6% suitable habitat.

**Table 5:** Total area (nm^2^) of modeled habitat suitability within existing National Marine Sanctuary boundaries.

	Habitat suitability					
	0	1	2	3	4	Suitable (>0)
*Olympic Coast (2,854 nm^2^)*						
Alcyoniina	2,478	53	61	129	134	376 (13%)
Antipatharia	2,587	210	43	14	-	266 (9%)
Calcaxonia	2,351	119	103	230	51	503 (18%)
Holaxonia	1,571	487	252	388	155	1,282 (45%)
Scleractinia	2,262	277	188	127	-	592 (21%)
Scleraxonia	2,307	64	82	346	54	546 (19%)
All Taxa –0.5	1,820	184	216	327	306	1,033 (36%)
All Taxa –0.75	2,515	126	63	56	94	338 (12%)
*Monterey Bay (5,465 nm^2^)*						
Alcyoniina	3,408	343	234	547	934	2,057 (38%)
Antipatharia	4,488	335	407	235	-	977 (18%)
Calcaxonia	3,959	235	227	628	417	1,507 (28%)
Holaxonia	3,951	377	264	459	415	1,514 (28%)
Scleractinia	3,731	362	805	567	-	1,735 (32%)
Scleraxonia	4,662	203	163	278	159	803 (15%)
All Taxa –0.5	2,637	479	418	704	1,227	2,828 (52%)
All Taxa –0.75	4,716	131	137	204	278	749 (14%)
*Gulf of Farallones (1,149 nm^2^)*						
Alcyoniina	898	11	10	30	200	251 (22%)
Antipatharia	460	308	266	115	-	689 (60%)
Calcaxonia	917	12	17	84	118	232 (20%)
Holaxonia	788	109	35	67	149	360 (31%)
Scleractinia	663	150	210	125	-	486 (42%)
Scleraxonia	970	17	37	94	31	179 (16%)
All Taxa –0.5	598	130	97	85	239	551 (48%)
All Taxa –0.75	964	33	26	44	81	184 (16%)
*Cordell Bank (469 nm^2^)*						
Alcyoniina	346	13	15	32	64	123 (26%)
Antipatharia	400	32	33	4	-	69 (15%)
Calcaxonia	350	4	9	59	48	119 (25%)
Holaxonia	295	50	41	38	46	174 (37%)
Scleractinia	202	108	117	43	-	267 (57%)
Scleraxonia	431	15	15	8	0	38 (8%)
All Taxa –0.5	241	33	33	45	118	228 (49%)
All Taxa –0.75	441	9	5	6	8	28 (6%)
*Channel Islands (1,299 nm^2^)*						
Alcyoniina	959	61	45	123	110	339 (26%)
Antipatharia	1,278	16	4	0	-	21 (2%)
Calcaxonia	1,083	41	49	110	16	215 (17%)
Holaxonia	809	136	99	217	37	489 (38%)
Scleractinia	261	138	412	487	-	1,037 (80%)
Scleraxonia	1,143	12	123	20	0	155 (12%)
All Taxa –0.5	507	177	174	203	238	792 (61%)
All Taxa –0.75	1,184	51	31	21	11	114 (9%)
*Entire modelled area (284,863 nm^2^)*						
Alcyoniina	257,486	7,315	4,577	5,994	9,491	23,7378 (9.6%)
Antipatharia	269,471	7,477	4,191	3,725	-	15,393 (5.4%)
Calcaxonia	261,266	6,839	4,081	7,506	5,172	23,598 (8.3%)
Holaxonia	264,281	7,688	4,330	5,462	3,102	20,583 (7.2%)
Scleractinia	270,015	4,651	4,874	5,324	-	14,849 (5.2%)
Scleraxonia	269,606	5,991	2,933	4,470	1,863	15,257 (5.4%)
All Taxa –0.5	248,226	9,352	6,767	8,586	11,932	36,637 (12.9%)
All Taxa –0.75	278,384	2,705	1,391	1,177	1,206	6,480 (2.3%)

## Spatial distribution of predicted habitat suitability and bottom trawl closures: Essential Fish Habitat (EFH) and Cowcod Conservation Area West (CCA-West)

**Figure 8:**
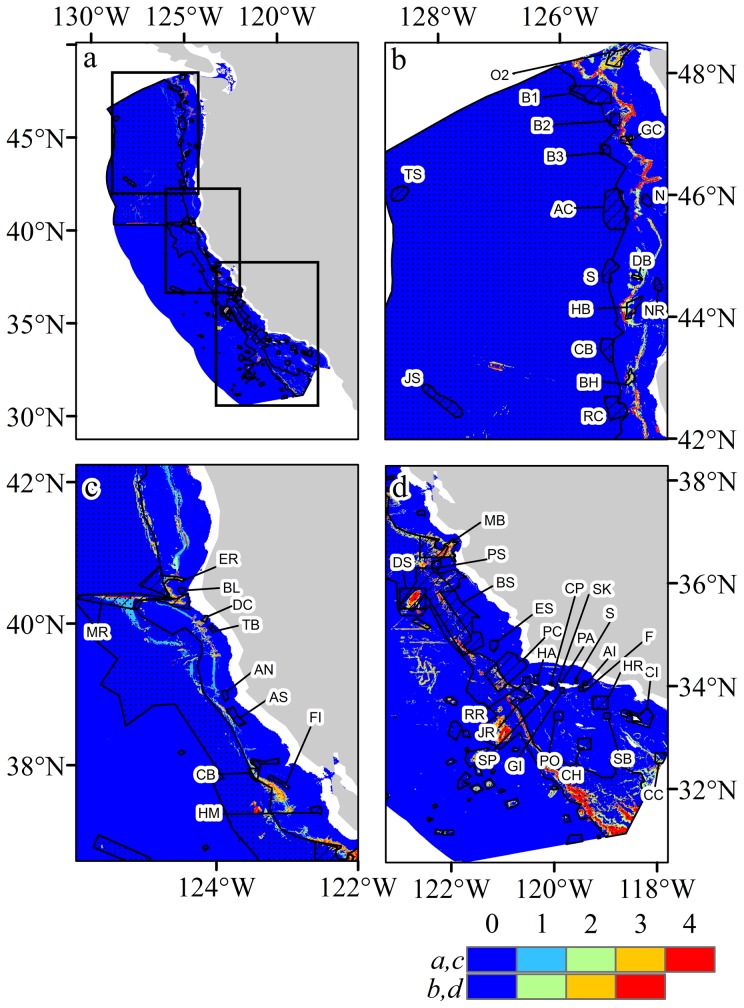
Spatial distribution of predicted habitat suitability and bottom trawl closures for areas designated Essential Fish Habitat (stippled) and CCA-West closures (hatched areas). a) Overview with 0.75 threshold suitability for all taxa, b) northern region (Washington and Oregon) and prediction for Scleractinia, c) central region (northern California) and prediction for Scleraxonia, d) southern region (central and southern California) and prediction for Antipatharia. Location abbreviations: O2: Olympic 2, B1: Biogenic 1, B2: Biogenic 2, B3: Biogenic 3, GC: Grays Canyon, N: Nehalem Bank/Shale Pile, AC: Astoria Canyon, TS: Thompson Seamount, S: Siletz Deepwater, DB: Daisy Bank/Nelson Island, NR: Newport Rockpile/Stonewall Bank, HB: Heceta Bank, CD: Deepwater off Coos Bay, BH: Brandon High Spot, RC: Rogue Canyon, JS: President Jackson Seamount, ER: Eel River Canyon, BL: Blunts Reef, MR: Mendocino Ridge, DC: Delgada Canyon, TB: Tolo Bank, AN: Pt. Arena North, AS: Pt. Arena South, CB: Cordell Bank Biogenic Area, FI: Farallon Islands/Fanny Shaol, HM: Half Moon Bay, MB: Monterey Bay/Canyon, PS: Point Sur Deep, BS: Big Sur Coast/Port San Luis, DS: Davidson Seamount, ES: East San Lucia Bank, PC: Point Conception, RR: Richardson Rock, JR: Judith Rock, HP: Harris Point, CP: Carrington Point, SP: South Point, SK: Skunk Point, S: Scorpion, PA: Painted Cave, AI: Anacopa Island, F: Footprint, HR: Hidden Reef/Kidney Bank, CI: Catalina Island, CC: Cowcod Conservation East Area, SB: Santa Barbara, CH: Cherry Bank, PO: Potato Bank, GI: Gull Island.

Coral habitat suitability maps for selected taxa and all taxa combined are depicted in Figure 8 (all taxa are shown in Figure S4) with overlays of essential fish habitat area closures and Cowcod Conservation Areas sourced from the Pacific Fishery Management Council. These area closures depict areas with fishing gear restrictions off Washington, Oregon, and California. Gear restrictions were established under NMFS' Final Rule to implement Amendment 19 to the Pacific Coast Groundfish Fishery Management Plan (71 FR 27408; May 11, 2006). Fishing with bottom trawl gear within these areas was prohibited to minimise adverse effects from fishing. All bottom contact gear was prohibited in waters surrounding Thompson Seamount, President Jackson Seamount, and several sites in the Channel Islands and Cordell Bank National Marine Sanctuaries. All bottom contact gear and any gear deployed deeper than 500 fathoms (914 m) was prohibited in the waters surrounding Davidson Seamount. There are two Cowcod Conservation Areas (East and West), but only CCA-East is designated as EFH. CCA-West is large in size and has high levels of habitat suitability for many of the coral taxa modelled. The EFH and the CCA-West areas are not the only bottom trawl closures present on the U.S. West Coast. There are also Rockfish Conservation Areas (RCAs) that extend along the entire length of the U.S. West Coast, but their boundaries can change throughout the year and are based on approximate depth contours between ∼100–150 fathoms (183–274 m). Both of these factors make quantitative assessment of RCA closures with predicted habitat suitability highly uncertain. In addition, California and Washington prohibit bottom trawling within their state territorial seas (out to 3 nautical miles). These trawl closures were not included in this analysis as the majority of suitable coral habitat is found in deeper areas outside state territorial waters.

## Northern Region (42°N to 48°N, Washington and Oregon)

Significant areas of high probability coral habitat for Alcyoniina were predicted off the coast of Washington state (Figure 5a and Figure S4). The areas with high probabilities that remain open to bottom contact gear include the entire western boundary of the OCNMS. These include regions adjacent to existing EFH area closures Biogenic 1 and Biogenic 2. Highly suitable habitat also was identified between existing EFH area closures Grays Canyon and Nehalem Bank/Shale Pile, along the western boundary of Hecata Bank, along the western boundary of Bandon High Spot, and areas between Bandon High Spot and Rouge Canyon EFH. The majority of Antipatharian predicted habitat was located in existing EFH area closures in this region; the exception to this are the predicted areas located between Grays Canyon and Nehalem Bank/Shale Pile, and within the OCNMS (Figure S4b). The most significant predicted habitat areas for Calcaxonia, which are not currently contained in EFH area closures, include waters north and south of Biogenic 1 and 2 and areas between Grays Canyon and Nehalem Bank/Shale Pile (Figure S4c). The predicted habitat pattern for Holaxonia was similar to that of Alcyoniina with two additional areas being highly suitable. These areas are located directly east of Nehalem Bank/Shale Pile and Heceta Bank (Figure S4d). Predictions for Scleractinia were limited in this region to a narrow depth band almost all of which is in areas open to bottom trawl gear (Figure S4e). High probability areas for Scleractinia were identified within the OCNMS and in a narrow depth band between Biogenic 2, Grays Canyon, and Nehalem Bank/Shale Pile. Areas west of Heceta Bank and Bandon High Spot were also identified, in addition to the area east of Rogue Canyon. The majority of predicted habitat for Scleraxonia that remains in open trawling areas is located within the OCNMS and between Grays Canyon and Nehalem Bank/Shale Pile (Figure S4f).

## Central Region (36°N to 42°N, Northern California)

The majority of high probability areas predicted for Alcyoniina occurred in areas currently open to bottom trawl gear (Figure S5a). These include areas between Rogue Canyon and Eel River Canyon and between Blunts reef and Pt. Arena South Biogenic Area. High probability areas for Antipatharia are almost completely contained within existing EFH area closures in this region (Figure S5b). Calcaxonia predicted habitat that remains in open trawl areas appears to follow the 700 fathoms (1,280 m) contour line between Rogue Canyon and Blunts Reef and also between Pt. Arena North and Cordell Bank Biogenic Area (Figure S5c). The majority of predicted Holaxonia and Scleractinian habitat are located in areas currently open to bottom trawl gear (Figure S5d and S5e). Scleraxonia habitat was predicted mostly in existing EFH area closures, but high probability areas were identified in areas open to bottom trawling in a narrow depth band between Rogue Canyon and Blunts Reef and again from Delgada Canyon to Pt. Arena South Biogenic area (Figure S5f).

## Southern Region (30°N to 38°N, Central and Southern California)

Predicted habitat for Alcyoniina was limited predominately to the continental shelf in this region. The majority of predicted Alcyoniina habitat in the northern extents of the mapped area is located within the boundaries for CBNMS, GFNMS and MBNMS (Figure S6a). Predicted habitat for Alcyoniina was identified in areas remaining open to bottom trawl gear in the southern extent of the mapped areas including the waters surrounding existing EFH area closures: Harris Point, Potato Bank, Hidden Reef/Kidney Bank, Catalina Island, and Cowcod Conservation Area East. High probability areas for Alcyoniina were also predicted along the northern boundary of the CINMS (north of San Miguel, Santa Rosa and Santa Cruz Islands). Predicted Alcyoniina habitat in the southern extent of the mapped area along the shelf break is mostly contained in existing EFH area closures. Most of the high probability areas for predicted Antipatharian habitat were located in current EFH area closures; the exception was the area approximately 65–200 km south of Davidson Seamount (Figure S6b). The majority of predicted Calcaxonia habitat in the region is located within current EFH area closures, but high probability areas remain open to bottom trawl gear in the southern extent of the mapped region in waters adjacent to Potato Bank, Catalina Island, and Cowcod Conservation Area East EFH area closures (Figure S6c). The majority of Holaxonia predicted habitat was identified in areas open to bottom trawl gear, most of which occurs within the boundaries of the CBNMS, GFNMS, and MBNMS (Figure S6d). High probability areas were also predicted in trawl areas surrounding Cowcod Conservation Area East. The majority of predicted Scleractinian habitat in the region was identified in areas currently open to bottom trawl gear (Figure S6e). Most of this habitat is limited to a depth range of ∼50–400 m (along the coast and islands) and a ∼3,500 km^2^ deep-water area south of Davidson Seamount. Scleractinian coral presence records documented in this deep-water region were *Fungiacyathus marenzelleri*. Most Scleraxonia habitat was identified in areas currently designated as EFH area closures (Figure S6f). High probability habitat results in areas open to bottom trawl gear include the waters surrounding to Farallon Islands/Fanny Shoal, Monterey Bay/Canyon, and Catalina Island.

## Bottom-trawl intensity

**Figure 9:**
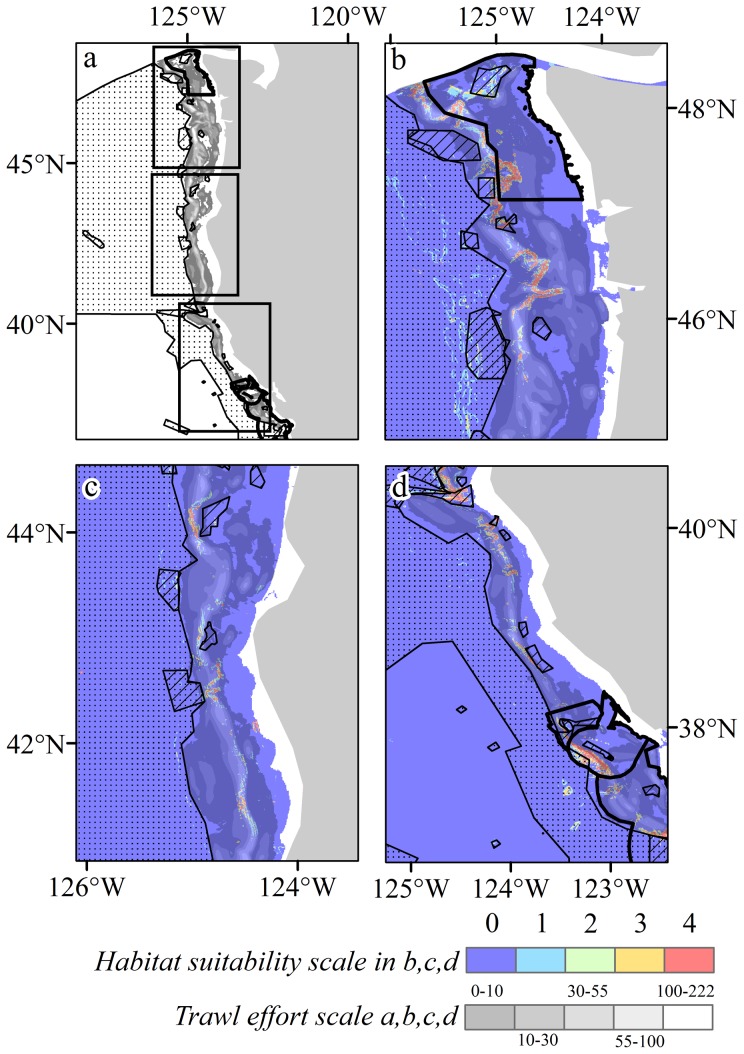
Fishing effort data overlain with predicted habitat from the 0.75 threshold all taxa model. a) Overview with fishing intensity grid, frames indicate location of panels b, c and d. b) Northern region, c) Central region and d) Southern region. Bold black areas in panel b, c, d indicate the locations of National Marine Sanctuaries, the hatched areas indicate locations of CCA-West closures and the stippled areas indicate Essential Fish Habitat closures. The upper legend (0–4) shows habitat suitability, higher is more suitable. The lower scale shows fishing intensity (km per km^2^).

**Figure 10:**
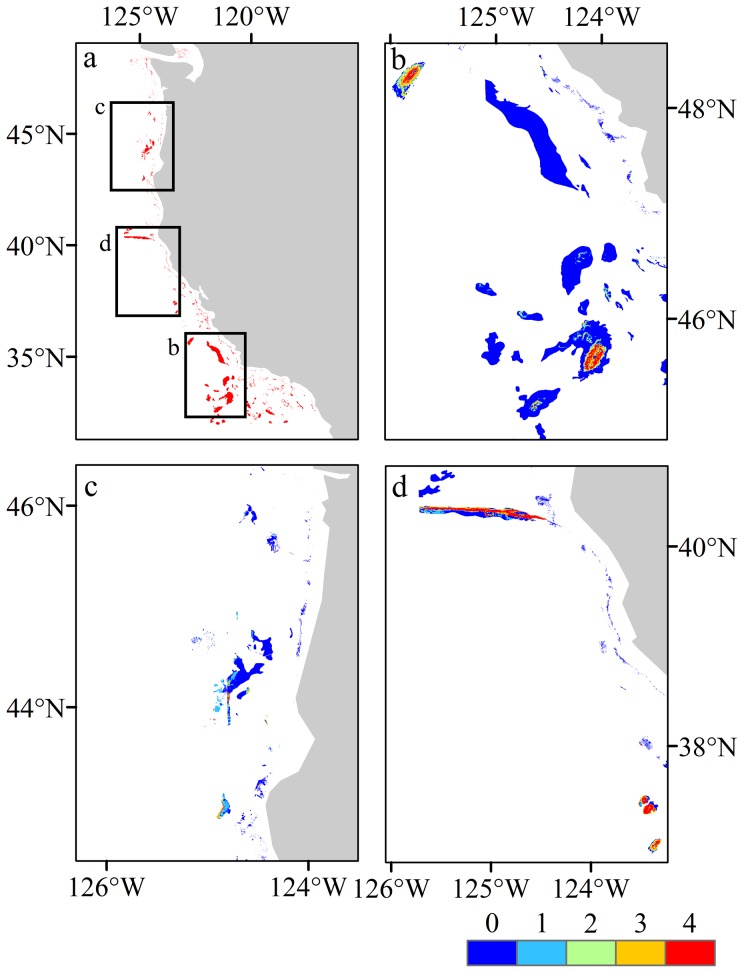
Habitat suitability only in areas with probable hard substrate. a) The distribution of probable hard substrate in red, b) the 0.75 threshold all taxa model, c) the Holaxonia model and d) the Alcyoniina model. The scale bar shows the habitat suitability (0–4, with 4 being high).

The bottom-trawl intensity data obtained from Whitmire [40] only covered the northern region between 33°N and 45°N and became fragmented below 37°N (Figure 9). The layer covers the shelf, and is largely constrained to depths shallower than 1,000 m. Much of this area is open for trawling, with EFH closures generally being deeper, however, as the intensity data does not cover the whole shelf, it is difficult to draw parallels between protection and trawling activity. There are areas where trawling activity overlaps with predicted suitable habitat (Figures 9 and 10), some areas are enclosed within closed areas but the majority falls outside areas of protection. Using the 0.75 logistic threshold-all-taxa model provided a spatially constrained prediction that focuses on areas with the highest predicted suitability. In the Northern region, and the Olympic Coast National Marine Sanctuary, there are several areas with high trawling intensity and high suitability, the majority of which falls outside of any designated area (Figure 9b). Within the Central region there are again areas of high suitability that fall outside of EFH areas such as adjacent to Heceta Bank, Brandon High Spot and Rogue Canyon (Figure 9c and see Figure 8 for locations of EFH areas). In the Southern region, Mendocino Ridge captures an area of high suitability with no trawling intensity data but further south adjacent to Delgada Canyon and Tolo Bank there are large areas of suitability with moderate trawling intensity (Figure 9d). Intersecting the trawling intensity grid with the habitat suitability classes for the whole region shows that there are areas of high suitability that are trawled at moderate levels compared to lower suitability classes, but the level of trawling for habitat suitability classes 1 and 4 are similar in contrast to 2 and 3, indicating that it is possible that trawlers are focusing both on areas that are not likely to contain corals (i.e. suitability values of 1) and are also targeting areas that do contain corals (i.e. suitability values of 4).

## Substrate data

The substrate data showed that for the area available, 91% of the shelf was described as probable soft sediment, 1% was probable mixed hard and soft sediment and 8% was classified as hard substrate (Figure 10a). To determine how much suitable habitat fell within areas of each probable substrate type, the modeled suitability layers for each taxa were spatially intersected with each substrate class and the proportion of area enumerated. By contrasting this, it was possible to provide an estimation of the level of over-prediction within the habitat suitability models (Figure 10 and Figure S7). In general, the majority of predicted habitat was found to fall within areas of probable soft sediment for all taxa (Table 6). However, for areas that were predicted as higher suitability (>2 on the habitat suitability scale), the proportion of predicted suitable habitat that fell within hard substrate area generally increased, indicating a link between the variables used in the model, the taxa niche and where hard substrate was found.

**Table 6:** Proportion of predicted habitat (higher values indicate more suitable) in relation to different substrate type for each taxa.

	Habitat suitability				
Sediment type	0	1	2	3	4
Alcyoniina					
Hard	6.8%	7.5%	10.4%	12.2%	13.9%
Mixed	1.2%	1.4%	1.0%	0.6%	0.3%
Soft	91.9%	91.1%	88.6%	87.1%	85.7%
Antipatharia					
Hard	6.6%	16.7%	25.8%	33.3%	-
Mixed	1.2%	1.5%	0.2%	0%	-
Soft	92.2%	81.8%	74.1%	66.7%	-
Calcaxonia					
Hard	6.7%	7.9%	8.6%	12.3%	22.8%
Mixed	1.1%	1.6%	1.5%	0.8%	0.4%
Soft	92.1%	90.5%	89.9%	86.9%	76.8%
Holaxonia					
Hard	7.5%	6.5%	9.4%	13.1%	12.3%
Mixed	0.5%	4.3%	2.4%	4.1%	1.0%
Soft	92.0%	89.2%	88.2%	82.8%	86.7%
Scleractinia					
Hard	8.1%	5.9%	6.2%	9.4%	-
Mixed	0.7%	5.9%	2.7%	1.8%	-
Soft	91.1%	88.2%	91.1%	88.7%	-
Scleraxonia					
Hard	7.2%	7.2%	9.5%	16.4%	26.3%
Mixed	1.1%	1.3%	0.8%	2.3%	0.1%
Soft	91.7%	91.5%	89.7%	81.3%	73.6%
All Taxa –0.5					
Hard	7.0%	6.6%	8.8%	9.4%	12.9%
Mixed	0.7%	2.2%	2.2%	2.7%	1.3%
Soft	92.4%	91.2%	89.0%	87.9%	85.8%
All Taxa –0.75					
Hard	7.3%	17.1%	20.1%	22.7%	15.4%
Mixed	1.1%	2.4%	2.1%	2.1%	0.6%
Soft	91.6%	80.5%	77.9%	75.2%	84.1%

## Discussion

This study is the first attempt to model the potential distribution of deep-sea coral habitat for the U.S. West Coast EEZ. The approaches presented here are a significant improvement over previous regional efforts such as those in the North East Atlantic [3] and Canadian Shelf [1], [2]. In recent studies there have been significant advancements in parallel with the data presented in this study, especially with respects to identifying usable datasets for regional-scale habitat suitability modelling in the deep-ocean [12]–[14]. However, there still remain several limitations that must be considered when interpreting broad-scale model results.

### Unincorporated model variables and model accuracy

There are several variables that are important for coral settlement, growth and survival that were not included in the model because they do not exist at sufficient resolutions, a problem shared with all habitat suitability efforts [43]. These variables include benthic hard substrata, high-resolution current direction/velocity, and the distribution of mobile or benthic sediments. Many corals require hard substrata for colonisation and like depth; substrate tends to be highly variable over small spatial scales. Model results presented here will overpredict the amount of suitable habitat in some areas because fine-scale and moderate scale bathymetric features (10′s of metres to 300 metres), substrate, and current data are not available. It is likely that model results indicate suitable coral habitat in areas that are known soft bottom regions with high sediment loadings where corals are likely or known to be absent, as indicated by 91% of the shelf being classed as probable soft sediment [41]. By contrasting the amount of available hard substrate and the predicted suitability for many taxa, the level of over-prediction can be estimated (Table 6). For example, of the area of habitat predicted as unsuitable (habitat suitability of 0) for Alcyoniina, 6.8% fell in areas classed as probable hard substrate. This increased as habitat suitability increased to the highest value (4), where 14.2% of area fell in areas classed as probable hard and mixed substrate. This shows that the model for this species may overpredict in approximately 85.8% of the area, assuming that the hard substrate layer is accurate and that the distribution of this taxa is entirely dependent upon hard substrate that can be mapped at a 25 m×25 m resolution.

Data on the distribution of sediments is unfortunately scarce for much of the world's seafloor, a fact that urgently needs to be addressed by mapping programs around the world. In this study, the IOOS Surficial Geologic Habitat maps for the Washington and Oregon continental shelves (Version 2.2) were initially explored to determine whether or not this information could be used in the habitat suitability models to refine taxon niches but these data were not suitable due to incomplete coverage of the study area. The National Marine Fisheries Service produced a composite substrate dataset as part of their 5-year Essential Fish Habitat Review for the West Coast [42]. This dataset aggregated many sources into a standardised classification (hard, mixed and soft substrate) layer at 25 m×25 m resolution, but covered only the shallower parts of the shelf limiting its utility in this modelling effort. In addition, the layer was also provided with a confidence layer that stated low confidence for most of the area, with only shallower water having medium or high confidence in the probable substrate type. This layer was used to constrain the output of the habitat suitability models for each taxa to produce a focused dataset that highlights areas where the habitat is suitable for coral and areas of probable hard substrate overlap (Figure 10 and Figure S7).

Model results for all taxa combined undoubtedly overpredict as the suborders and orders modeled separately occupy different niches, depth ranges, and caution should be exercised when using all taxa combined model results. However, we introduced a constrained model by increasing the threshold to the 0.75 logistic suitability for all taxa, producing a model that was far more focused on areas of very high suitability. Assessment of model accuracy will be dependent on field operations to validate model predictions. In addition to several unincorporated datasets, the extent, quality, and availability of environmental, chemical and physical data are continually improving and should be incorporated in an iterative process with field surveys to refine predictions and reduce the number of false positives and negatives in habitat suitability models.

### Presence records

The limited number of coral presence records used to model habitat distribution for some coral taxa highlights the need for more targeted sampling to document coral locations. For example, very few presence localities for Suborders Filifera and Stolonifera were obtained and preliminary models suffered from significant overprediction and artificially high AUC scores. Low presence numbers could be due to coral rarity among these taxa and/or undersampling. The lack of coral records for these suborders resulted in the omission of these models from the analysis. Several recent studies have investigated the effectiveness and reliability of habitat suitability models constructed with low numbers of presences, a common problem for difficult to detect species (i.e. deep-sea corals) and those that have had limited systematic survey effort such as records from museum collections [44]. This does not preclude the possibility of modelling species distributions with low sample numbers, as Maxent is capable of producing acceptable models with relatively limited numbers of presences [37]. However, Maxent does appear to overpredict suitable habitat when using small presence datasets compared with other methods [37], [45]. In addition, grouping coral records at the order and suborder level undoubtedly combines coral taxa (family, genus, species) with different environmental niches. This is a recognised limitation of the approach, but one that is necessary due to taxonomic uncertainty and total number of coral records available.

### Model validation and targeting areas for field operations

Field validation of modeled habitat is needed to 1) Assess the accuracy of model predictions. 2) Refine models by identifying false positives and negatives. 3) Gauge the utility of these modelling methods for identifying deep-sea coral habitat in unsurveyed areas. The predicted habitat suitability results presented here are not meant to identify coral occurrences with pin point accuracy and are unlikely to achieve this based on currently available data. They are more useful for directing research effort to areas that have the highest probability of supporting deep-sea corals and identifying low probability areas that could be avoided to maximise time spent in high probability areas. Broad-scale predictive habitat results should be used in conjunction with multibeam surveys, geologic substrate maps and other tools to determine the most likely areas for harboring deep-sea corals. One additional complication for field validation efforts using these predictions is the current technological limitation of survey vehicles and equipment (i.e. ROVs, submersibles, drop cameras, etc.). The distribution of deep-sea corals within a single grid cell of these models (500 m×500 m) could be patchy [46] and could be missed on vehicle transects with limited range and narrow fields of view. To address this limitation and to improve the probability of locating undiscovered coral areas, research ships should first use multibeam surveys (in high probability areas) to identify substrate characteristics that can support deep-sea coral growth or identify corals (e.g. emergent hard substrata, coral rubble) and then move towards visual detection methodologies.

### Assessment of closures and trawl intensity

**Figure 11:**
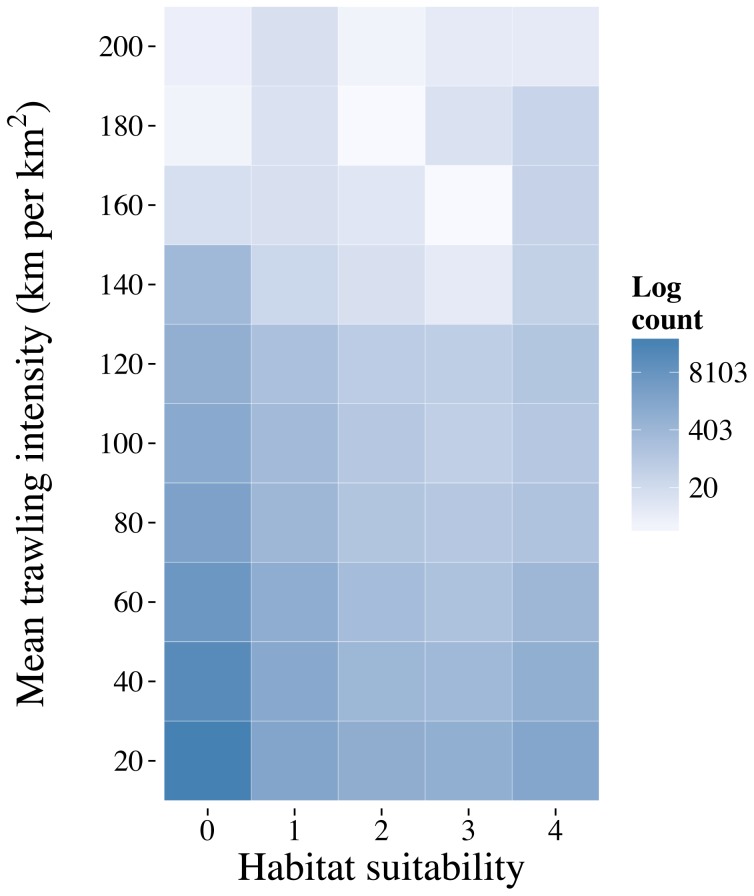
Heat plot of trawling intensity falling within habitat suitability classes for the 0.75 threshold all taxa model. Darker colours indicate higher cell counts compared to lighter, the plot shows that trawling intensity is greatest in cells classified as suitable unsuitable, however suitability classes 1 and 4 tend to have more trawl activity compared to classes 2 and 3.

Predictive models have been used to assess the suitability of existing protected areas in several areas including the North East Atlantic [14] and South Pacific [47]. Our findings are broadly similar, showing that the boundaries of U.S. National Marine Sanctuaries contained suitable habitat for corals above the average proportions of predicted suitable habitat throughout the entire study area (Table 5). Significant areas of highly suitable deep-sea coral habitat were modeled both within and outside existing NMS, EFH, and CCA-West closure boundaries. However, the majority of suitable habitat for Suborder Holaxonia and Order Scleractinia was predicted in areas outside of existing area closure boundaries. We also, however, identified numerous areas where areas of suitable habitat, from the most constrained model (75^th^ percentile) fell outside of current protection initiatives. This was particularly evident in the EEZ waters off of Washington and Oregon. Overlaying the spatially limited trawling intensity layer from Whitmire [40] revealed that the majority of intense trawling was outside of current closed areas (Figure 11), as expected, but many areas did not have intensity data available. In previous studies, data from vessel monitoring systems have shown that vessels will enter closed areas occasionally and that vessel behaviour may be linked to the establishment of a closed area [48]. There were several high suitability areas that had a higher trawling intensity than other areas (Figure 11), implying that there may be some overlap in areas that are being fished and that contain suitable habitat for corals. Trawling has been shown to be highly damaging to corals, especially reef-forming scleractinans [49]. Emergent epifauna including octocorals will be adversely affected particularly in areas with repeated, high intensity trawling [50].

## Conclusion

The U.S. West Coast has been relatively well researched with respect to the distribution of deep-sea coral species when compared to other regions of the world's oceans. However, significant spatial bias in sampling effort exists in the region and future field research efforts should be directed to unsampled areas to improve habitat predictions. Target areas for future field operations should include high probability areas identified in this study in regions of the U.S. West Coast EEZ that have not been visited. Predictive habitat model results are the only data available for areas that have not been sampled and should be used in in conjunction with other tools, data (i.e. geologic maps, multibeam bathymetry, etc.), and field surveys (where available) to help managers identify potential coral areas that remain at risk from human activity.

## Supporting Information

Figure S1 **Predicted suitability for the area of the Olympic Coast National Marine Sanctuary, a) Alcyoniina, b) Antipatharia, c) Calcaxonia, d) Holaxonia, e) Scleractinia, f) Scleraxonia, g) all taxa (50% threshold), h) all taxa (75% threshold).**
(TIF)

Figure S2 **Predicted suitability for the area of the Cordell Bank, Gulf of the Farallones, and Monterey Bay National Marine Sanctuaries, a) Alcyoniina, b) Antipatharia, c) Calcaxonia, d) Holaxonia, e) Scleractinia, f) Scleraxonia, g) all taxa (50% threshold), h) all taxa (75% threshold).**
(TIF)

Figure S3 **Predicted suitability for the area of the Channel Islands National Marine Sanctuary, a) Alcyoniina, b) Antipatharia, c) Calcaxonia, d) Holaxonia, e) Scleractinia, f) Scleraxonia, g) all taxa (50% threshold), h) all taxa (75% threshold).**
(TIF)

Figure S4 **Predicted habitat suitability in the Northern Region with EFH area closures (stippled areas) and CCA-West closures (hatched areas) for a) Alcyoniina, b) Antipatharia, c) Calcaxonia, d) Holaxonia, e) Scleractinia, f) Scleraxonia, g) all taxa (50% threshold), h) all taxa (75% threshold).** For abbreviations, see Figure 8 in the manuscript.
(TIF)

Figure S5 **Predicted habitat suitability in the Central Region with EFH area closures (stippled areas) and CCA-West closures (hatched areas) for a) Alcyoniina, b) Antipatharia, c) Calcaxonia, d) Holaxonia, e) Scleractinia, f) Scleraxonia, g) all taxa (50% threshold), h) all taxa (75% threshold).** For abbreviations, see Figure 8 in the manuscript.
(TIF)

Figure S6 **Predicted habitat suitability in the Southern Region with EFH area closures (stippled areas) and CCA-West closures (hatched areas) for a) Alcyoniina, b) Antipatharia, c) Calcaxonia, d) Holaxonia, e) Scleractinia, f) Scleraxonia, g) all taxa (50% threshold), h) all taxa (75% threshold).** For abbreviations, see Figure 8 in the manuscript.
(TIF)

Figure S7 **Predicted habitat suitability for areas identified as probable hard substrate for a) Alcyoniina, b) Antipatharia, c) Calcaxonia, d) Holaxonia, e) Scleractinia, f) Scleraxonia, g) all taxa (50% threshold), h) all taxa (75% threshold).**
(TIF)

File S1 **Contains Tables S1–S10.** Table S1: Correlation matrix for 10000 randomly placed points within the model domain. Table S2: Correlation matrix for points where the taxon Alcyoniina was found (n = 791). Table S3: Correlation matrix for points where the taxon Antipatharia was found (n = 128). Table S4: Correlation matrix for points where the taxon Calcaxonia was found (n = 413). Table S5: Correlation matrix for points where the taxon Filifera was found (n = 11). Table S6: Correlation matrix for points where the taxon Holaxonia was found (n = 308). Table S7: Correlation matrix for points where the taxon Scleractinia was found (n = 203). Table S8: Correlation matrix for points where the taxon Scleraxonia was found (n = 277). Table S9: Correlation matrix for points where the taxon Stolonifera was found (n = 30). Table S10: Correlation matrix for points where the taxon all species were found (n = 1059).
(DOCX)

File S2 **Model outputs for each taxa as ArcGIS GeoTIFF files with ArcGIS Map Documents and categorical layer files.**
(ZIP)
