# Supplementary figures and images for: Predicted Deep-Sea Coral Habitat Suitability for the U.S. West Coast
Source: PLoS One. 2014 Apr 23;9(4):e93918. doi: 10.1371/journal.pone.0093918 (PMC3997739; doi:10.1371/journal.pone.0093918)

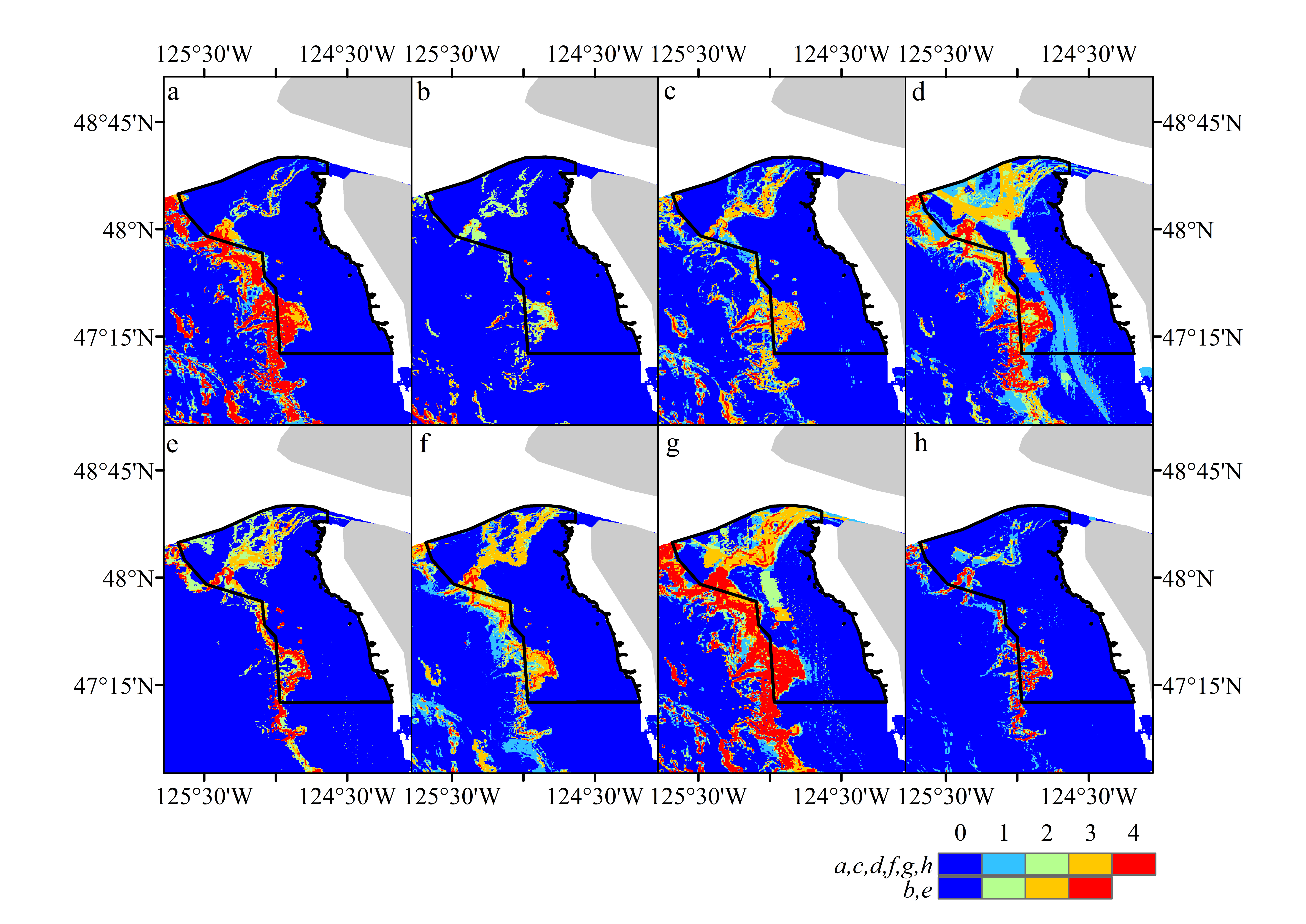

Supplement: Figure S1 — Predicted suitability for the area of the Olympic Coast National Marine Sanctuary, a) Alcyoniina, b) Antipatharia, c) Calcaxonia, d) Holaxonia, e) Scleractinia, f) Scleraxonia, g) all taxa (50% threshold), h) all taxa (75% threshold). (TIF) [file pone.0093918.s001.tif]

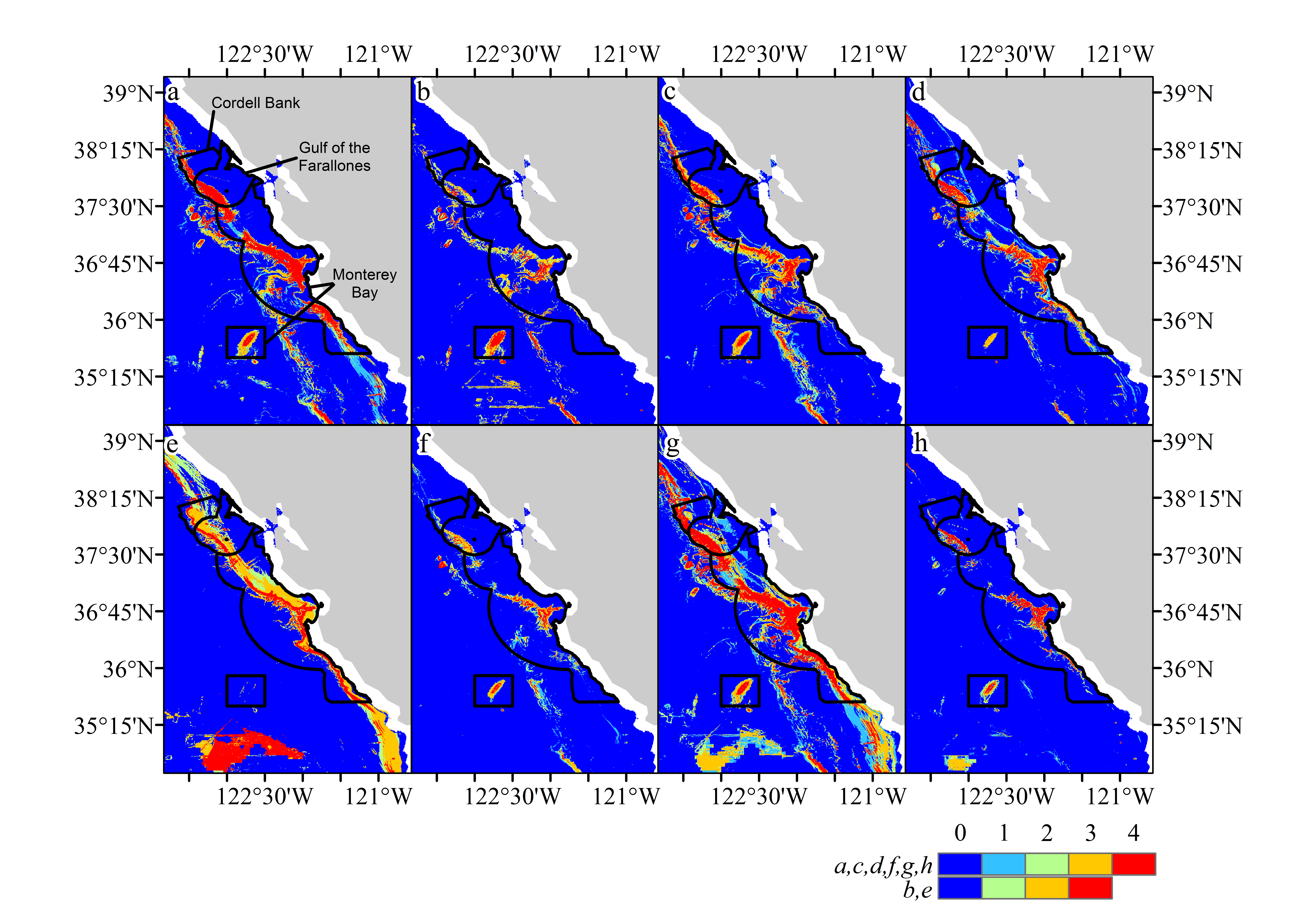

Supplement: Figure S2 — Predicted suitability for the area of the Cordell Bank, Gulf of the Farallones, and Monterey Bay National Marine Sanctuaries, a) Alcyoniina, b) Antipatharia, c) Calcaxonia, d) Holaxonia, e) Scleractinia, f) Scleraxonia, g) all taxa (50% threshold), h) all taxa (75% threshold). (TIF) [file pone.0093918.s002.tif]

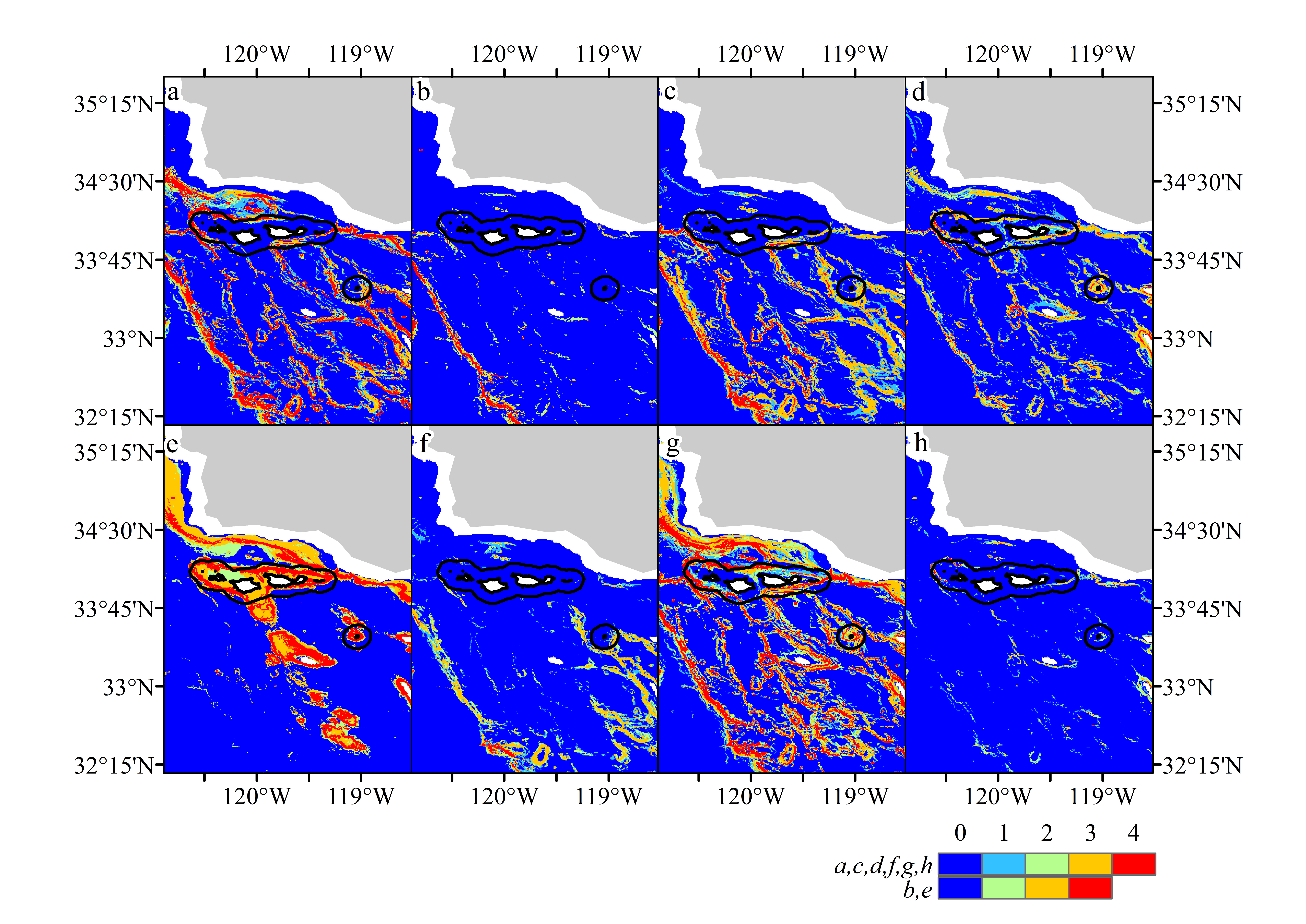

Supplement: Figure S3 — Predicted suitability for the area of the Channel Islands National Marine Sanctuary, a) Alcyoniina, b) Antipatharia, c) Calcaxonia, d) Holaxonia, e) Scleractinia, f) Scleraxonia, g) all taxa (50% threshold), h) all taxa (75% threshold). (TIF) [file pone.0093918.s003.tif]

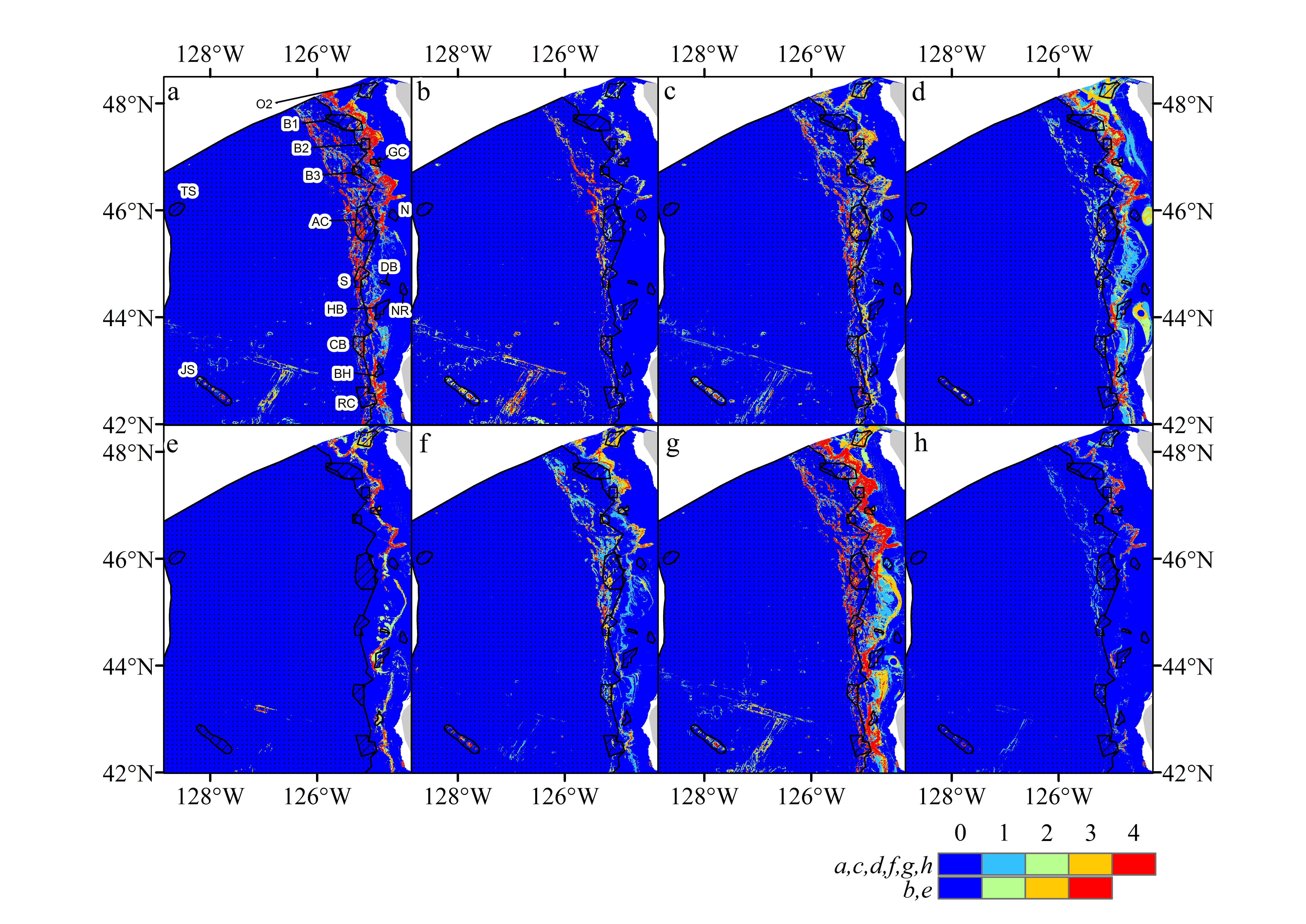

Supplement: Figure S4 — Predicted habitat suitability in the Northern Region with EFH area closures (stippled areas) and CCA-West closures (hatched areas) for a) Alcyoniina, b) Antipatharia, c) Calcaxonia, d) Holaxonia, e) Scleractinia, f) Scleraxonia, g) all taxa (50% threshold), h) all taxa (75% threshold). For abbreviations, see Figure 8 in the manuscript. (TIF) [file pone.0093918.s004.tif]

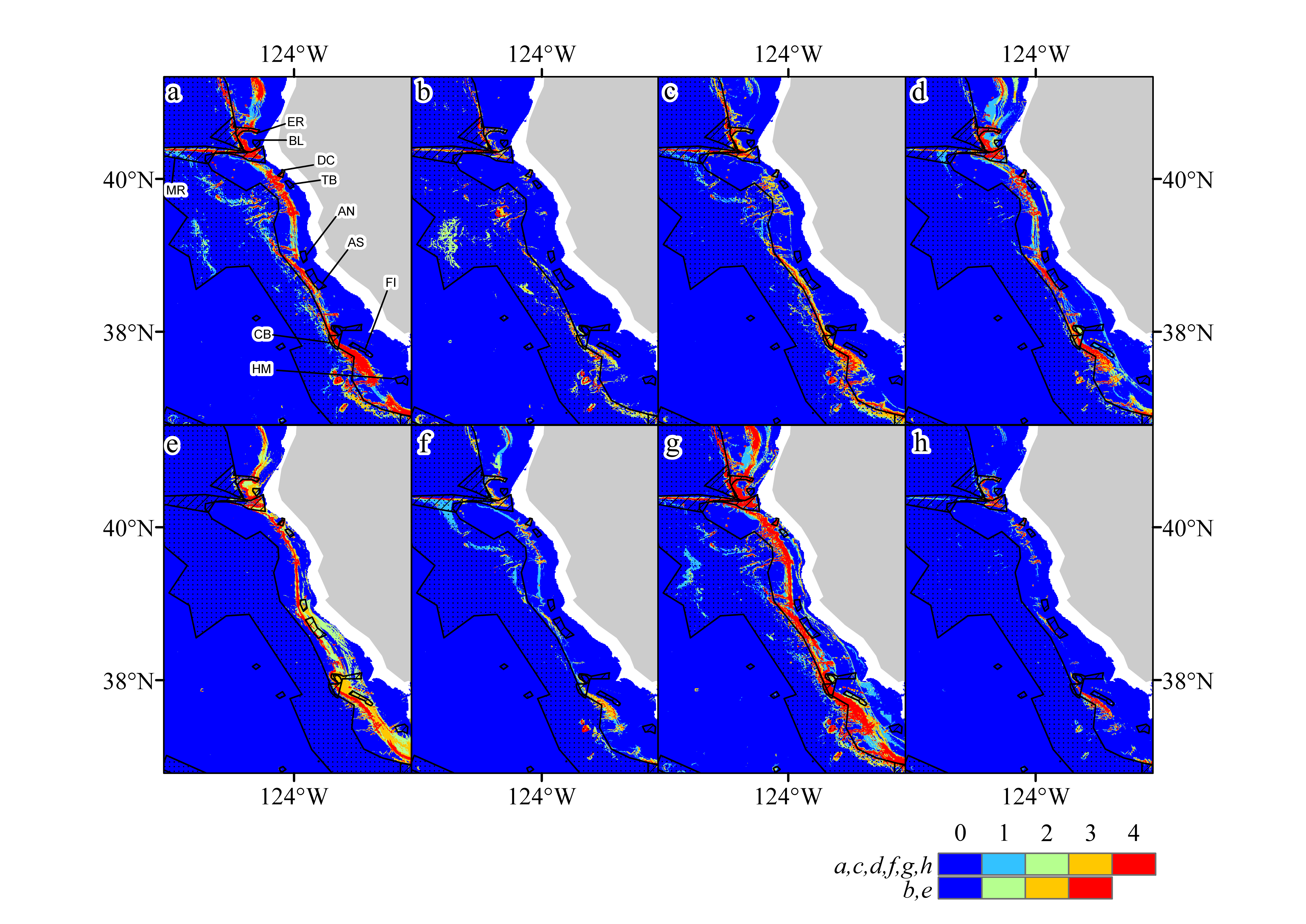

Supplement: Figure S5 — Predicted habitat suitability in the Central Region with EFH area closures (stippled areas) and CCA-West closures (hatched areas) for a) Alcyoniina, b) Antipatharia, c) Calcaxonia, d) Holaxonia, e) Scleractinia, f) Scleraxonia, g) all taxa (50% threshold), h) all taxa (75% threshold). For abbreviations, see Figure 8 in the manuscript. (TIF) [file pone.0093918.s005.tif]

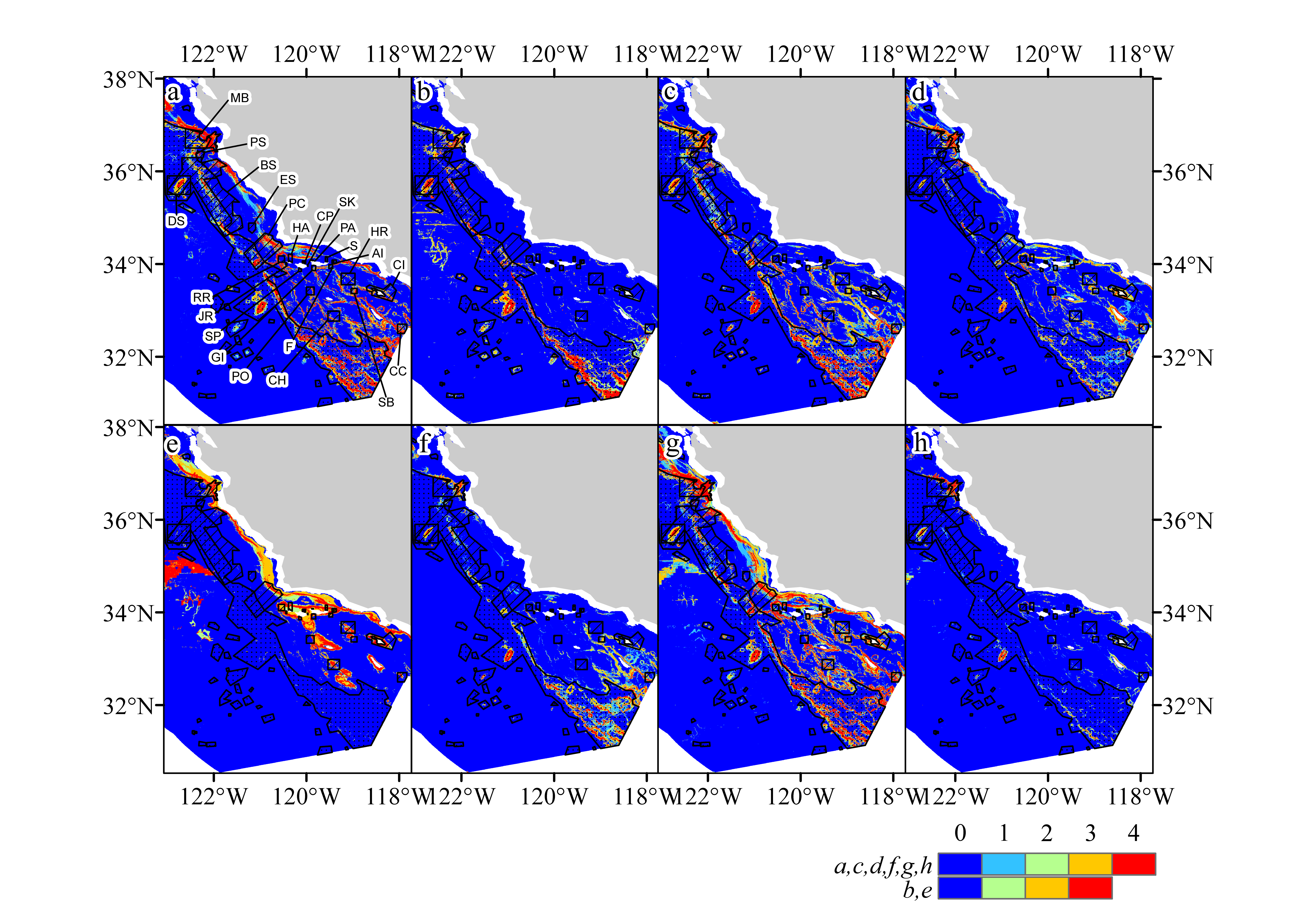

Supplement: Figure S6 — Predicted habitat suitability in the Southern Region with EFH area closures (stippled areas) and CCA-West closures (hatched areas) for a) Alcyoniina, b) Antipatharia, c) Calcaxonia, d) Holaxonia, e) Scleractinia, f) Scleraxonia, g) all taxa (50% threshold), h) all taxa (75% threshold). For abbreviations, see Figure 8 in the manuscript. (TIF) [file pone.0093918.s006.tif]

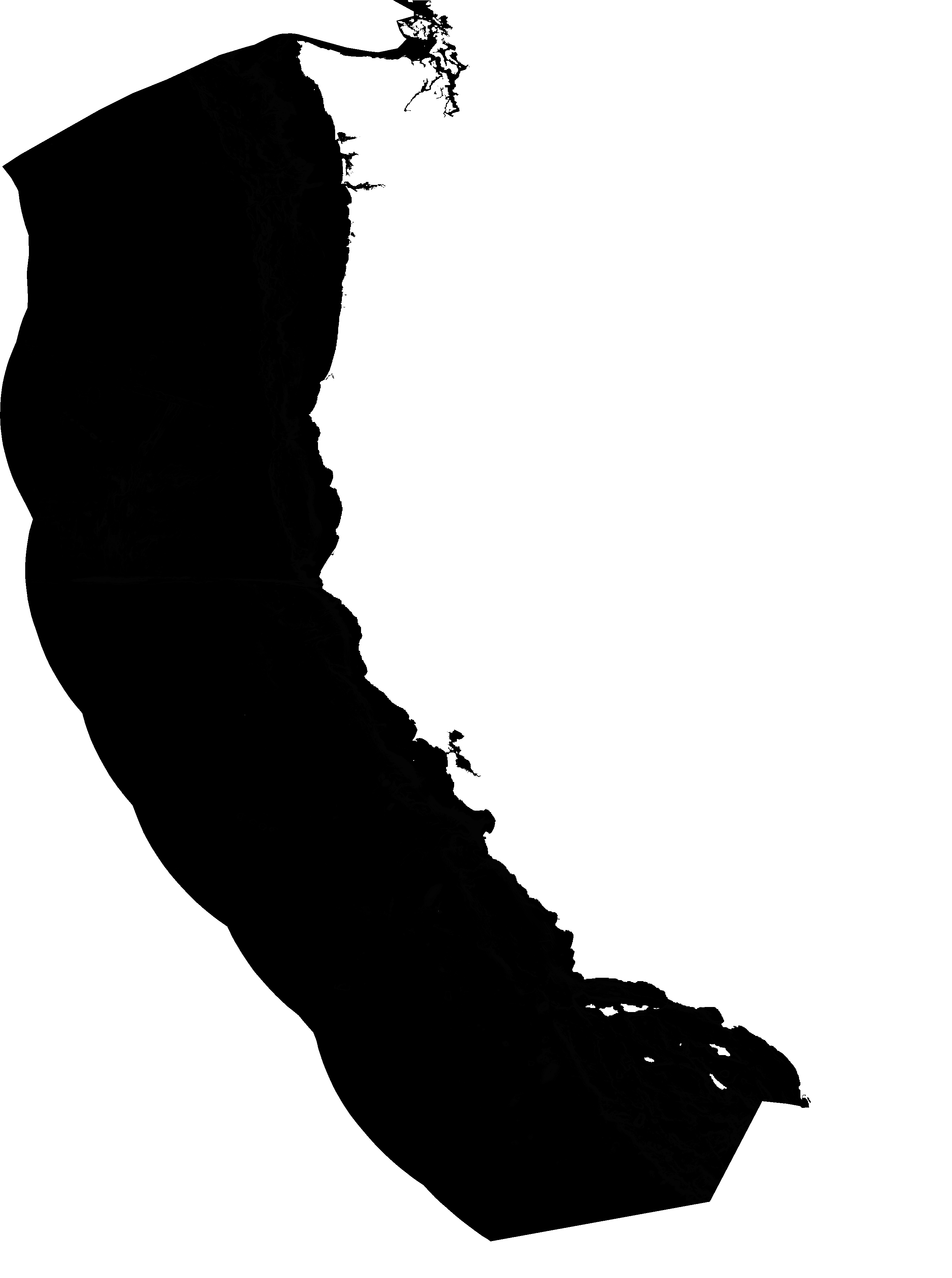

Supplement: File S2 — Model outputs for each taxa as ArcGIS GeoTIFF files with ArcGIS Map Documents and categorical layer files. (ZIP) [file pone.0093918.s009.zip › Alcyoniina_Full_Extent/alcyoniina_full_extent.tif]

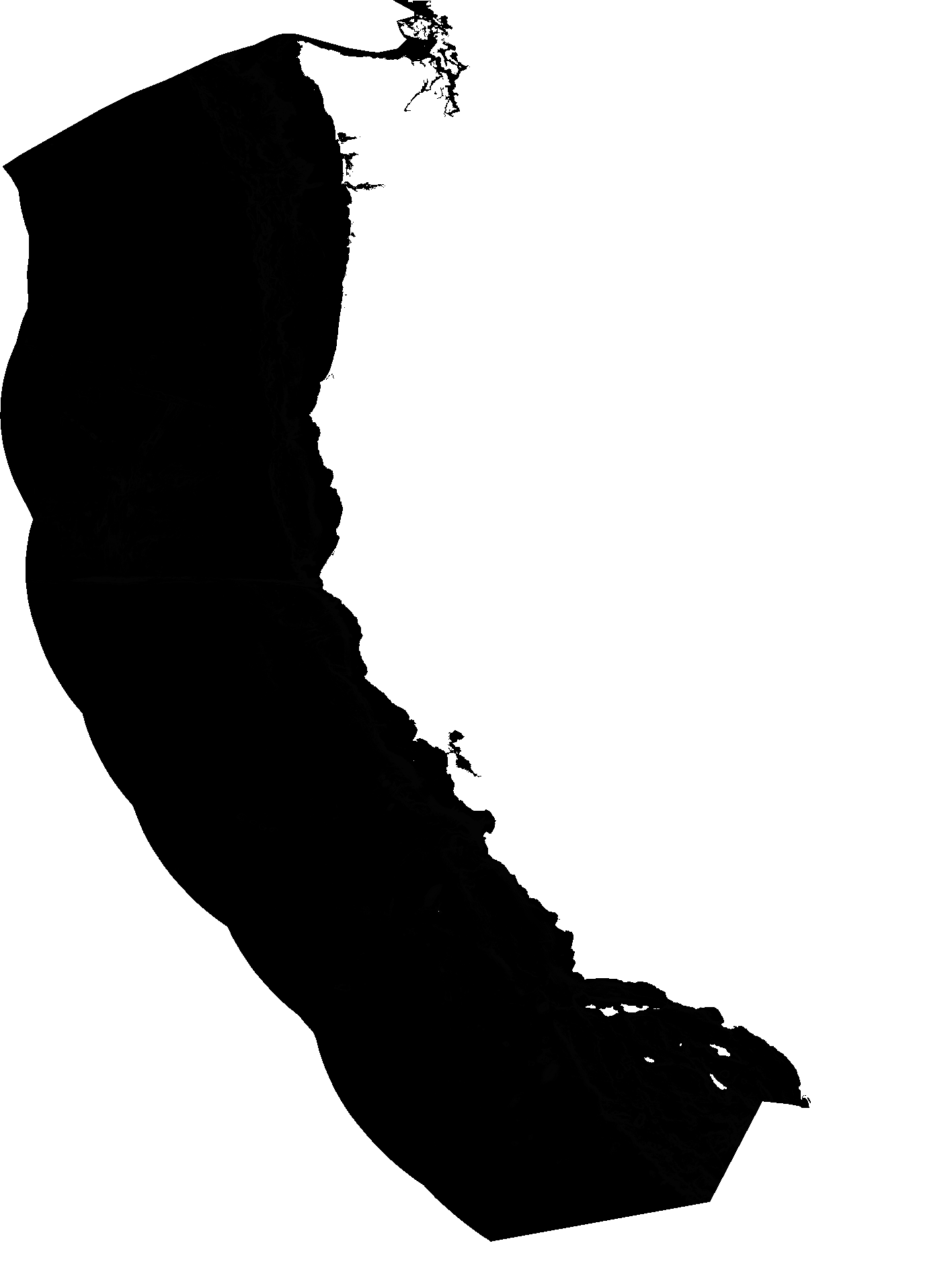

Supplement: File S2 — Model outputs for each taxa as ArcGIS GeoTIFF files with ArcGIS Map Documents and categorical layer files. (ZIP) [file pone.0093918.s009.zip › Alcyoniina_Full_Extent/alcyoniina_full_extent.tif.ovr]

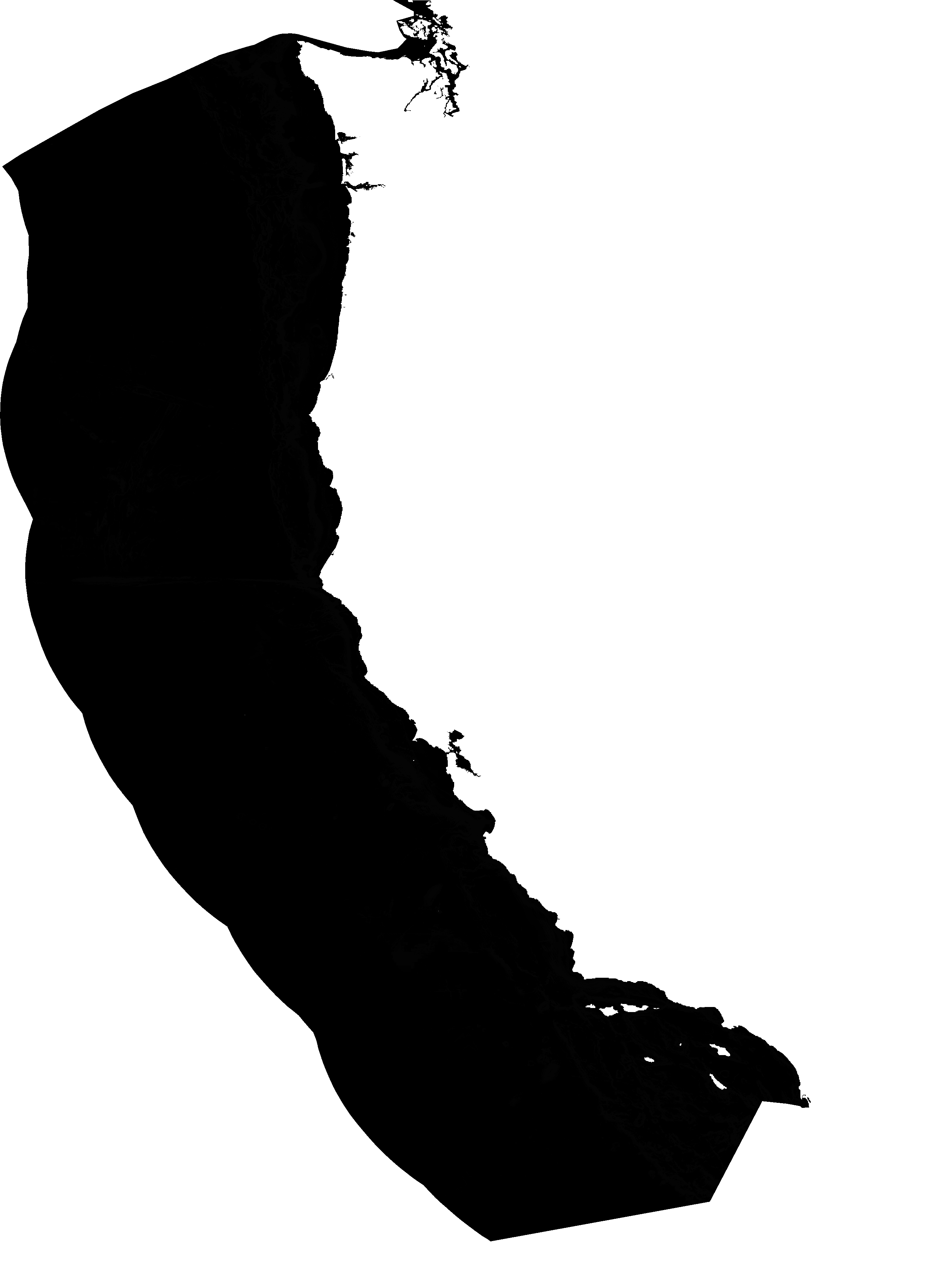

Supplement: File S2 — Model outputs for each taxa as ArcGIS GeoTIFF files with ArcGIS Map Documents and categorical layer files. (ZIP) [file pone.0093918.s009.zip › All_Taxa_50_Full_Extent/all_taxa_50_full_extent.tif]

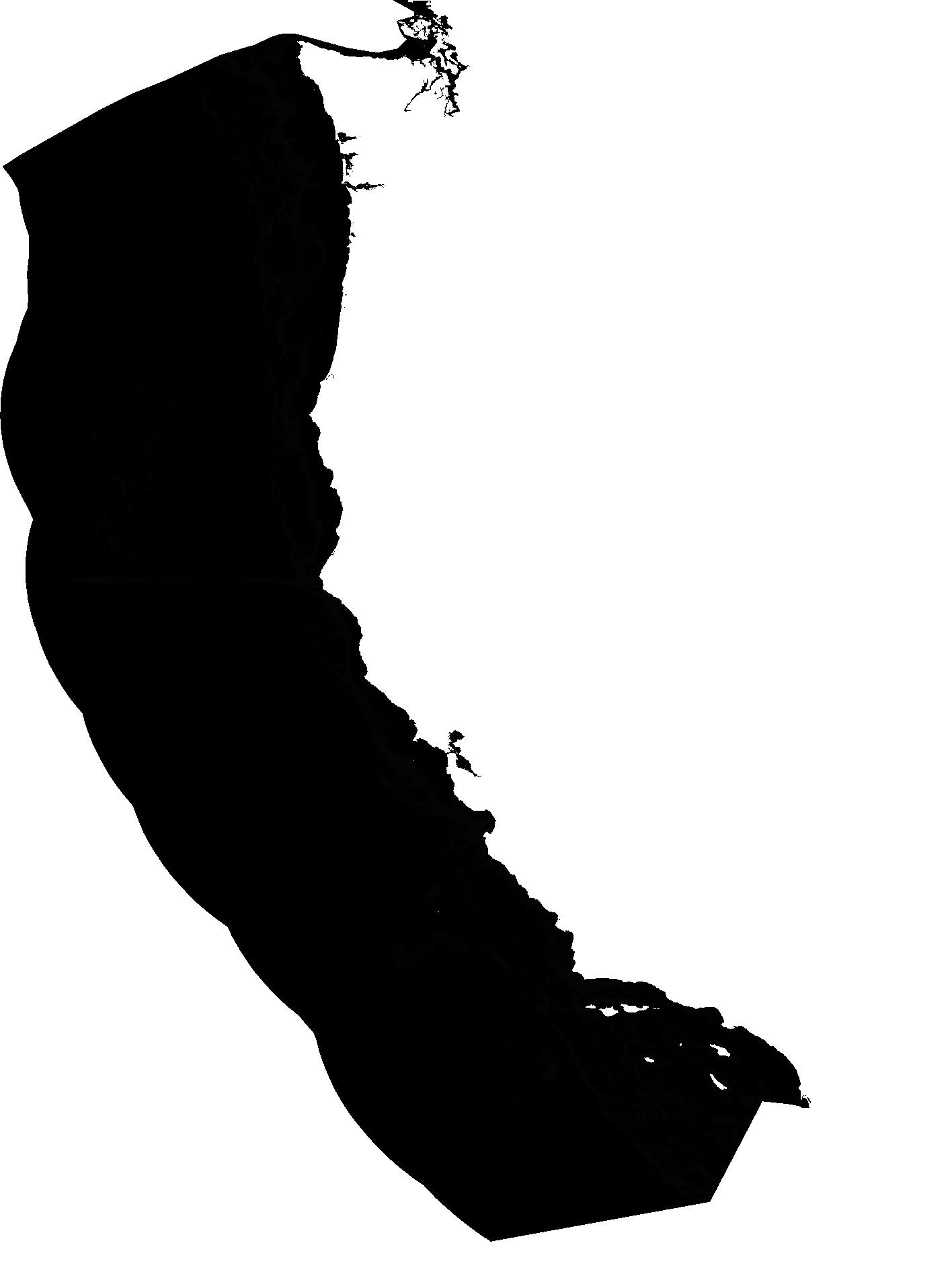

Supplement: File S2 — Model outputs for each taxa as ArcGIS GeoTIFF files with ArcGIS Map Documents and categorical layer files. (ZIP) [file pone.0093918.s009.zip › All_Taxa_50_Full_Extent/all_taxa_50_full_extent.tif.ovr]

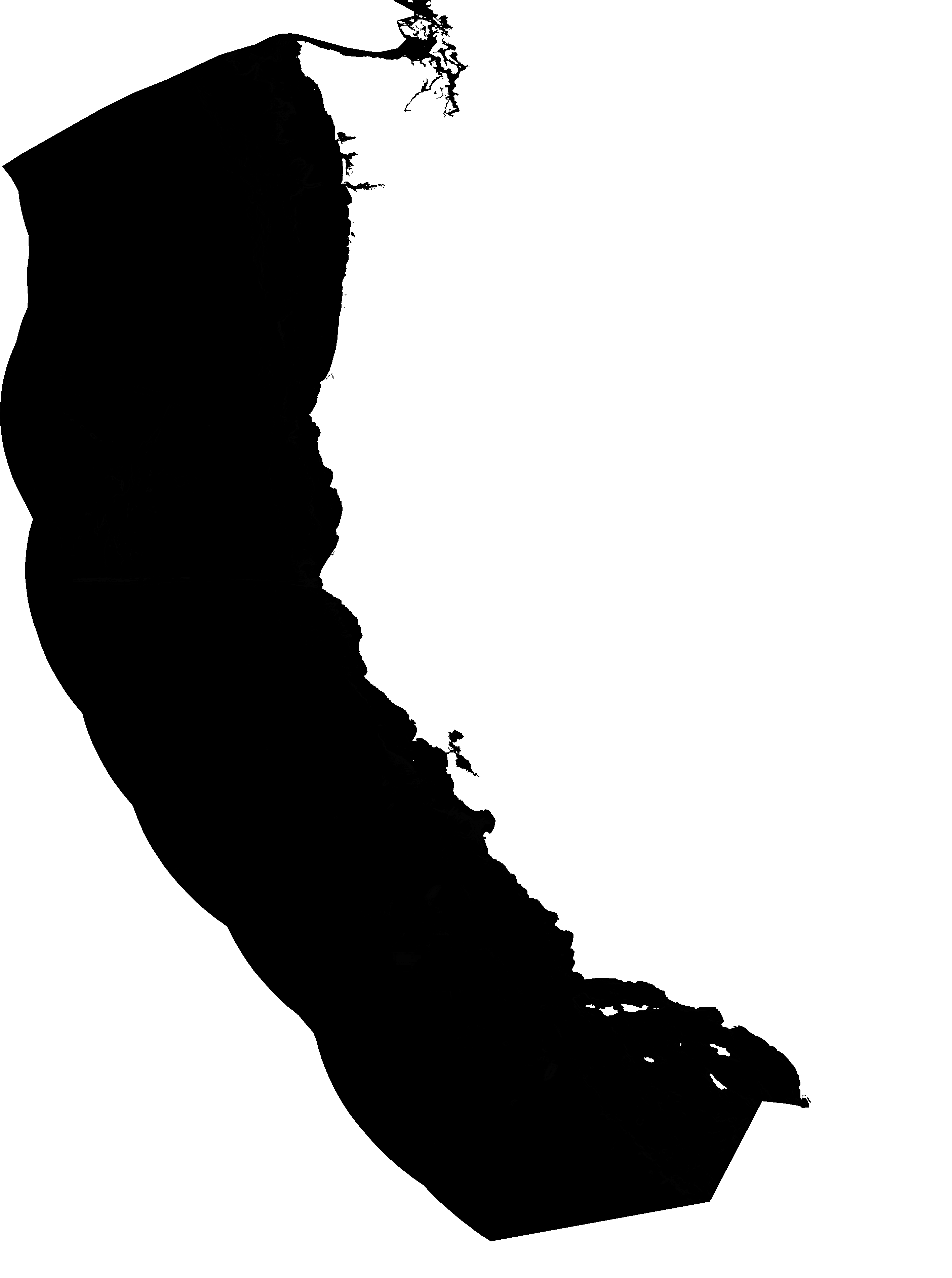

Supplement: File S2 — Model outputs for each taxa as ArcGIS GeoTIFF files with ArcGIS Map Documents and categorical layer files. (ZIP) [file pone.0093918.s009.zip › All_Taxa_75_Full_Extent/all_taxa_75_full_extent.tif]

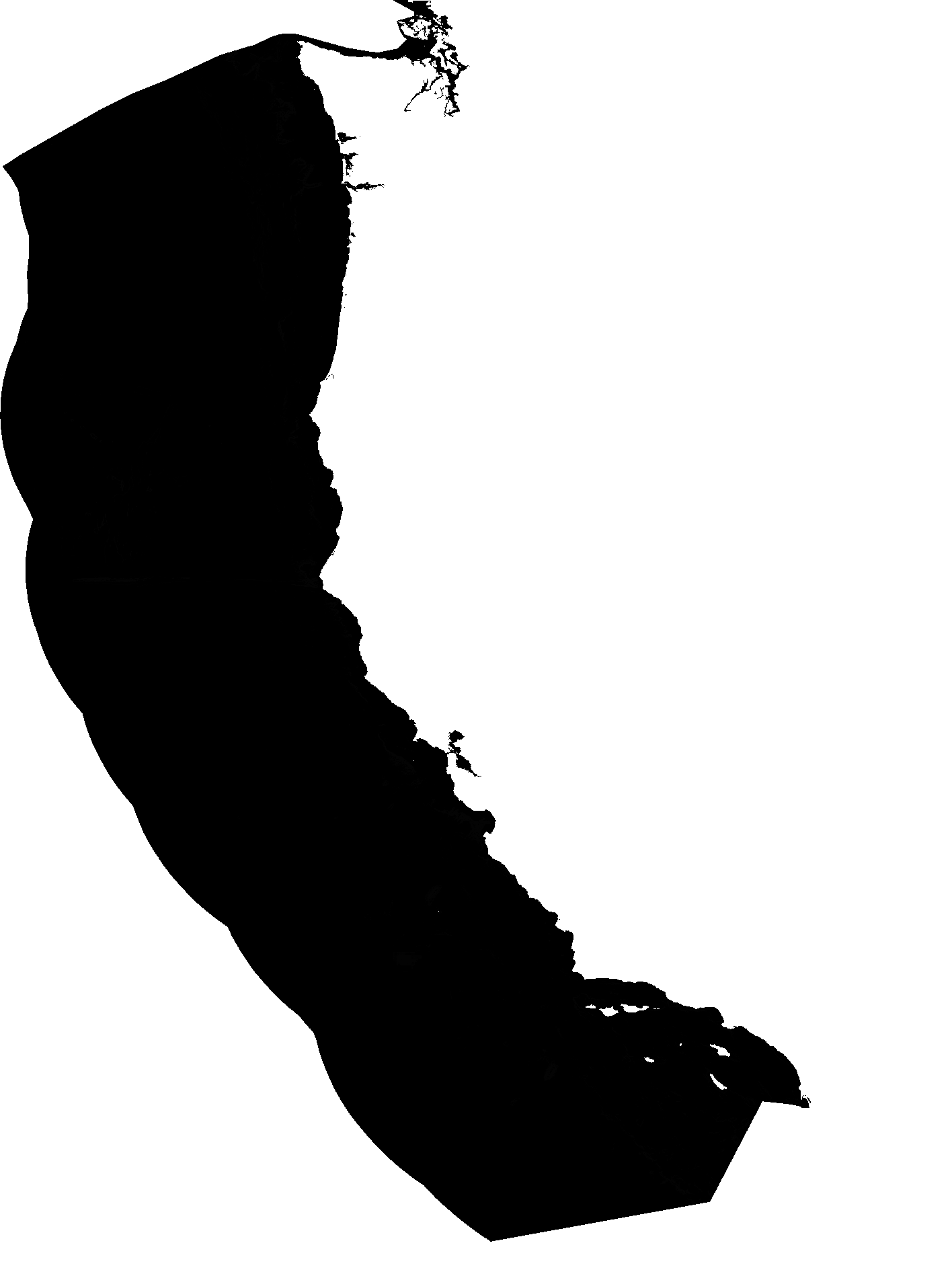

Supplement: File S2 — Model outputs for each taxa as ArcGIS GeoTIFF files with ArcGIS Map Documents and categorical layer files. (ZIP) [file pone.0093918.s009.zip › All_Taxa_75_Full_Extent/all_taxa_75_full_extent.tif.ovr]

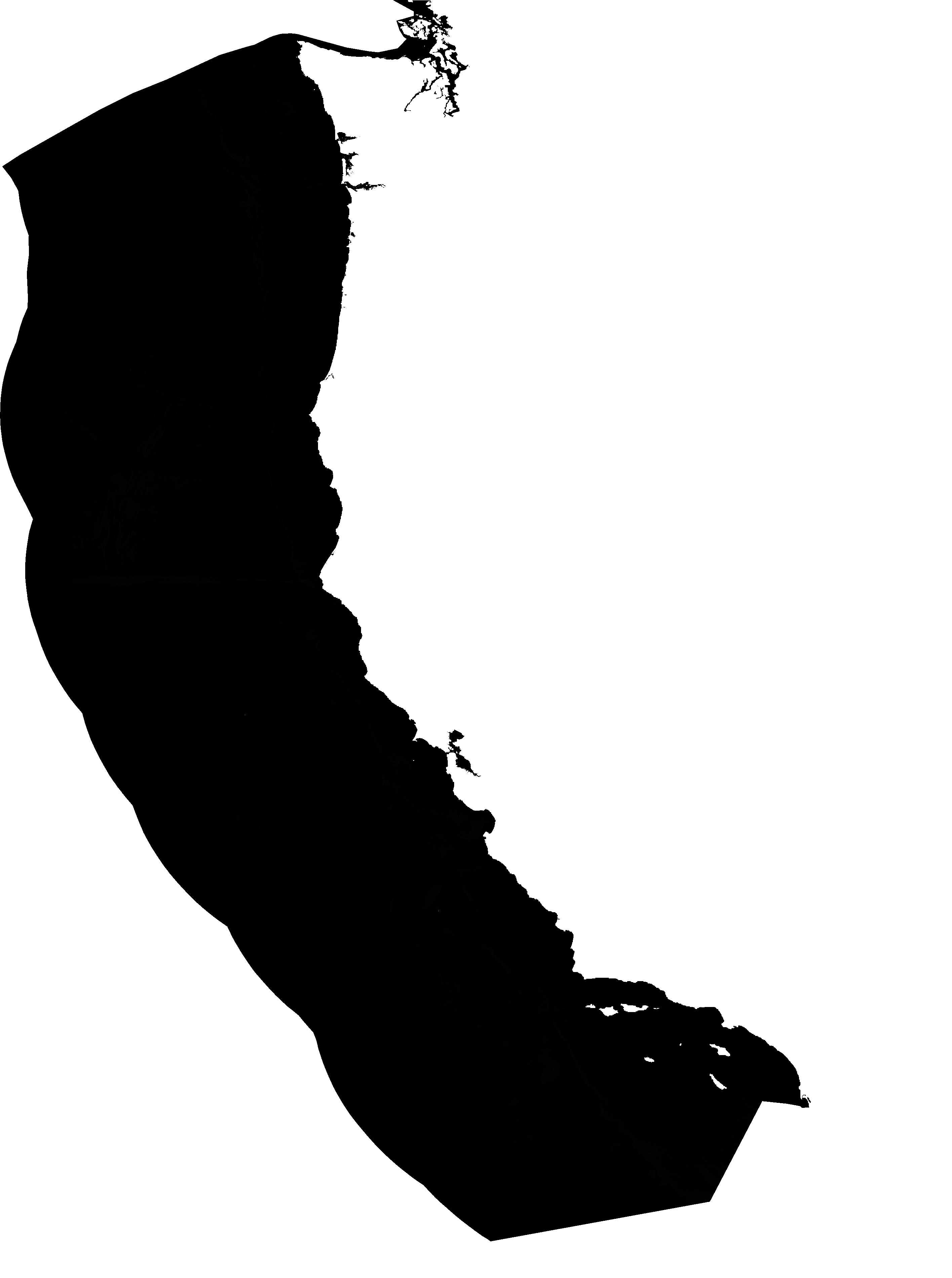

Supplement: File S2 — Model outputs for each taxa as ArcGIS GeoTIFF files with ArcGIS Map Documents and categorical layer files. (ZIP) [file pone.0093918.s009.zip › Antipatharia_Full_Extent/antipatharia_full_extent.tif]

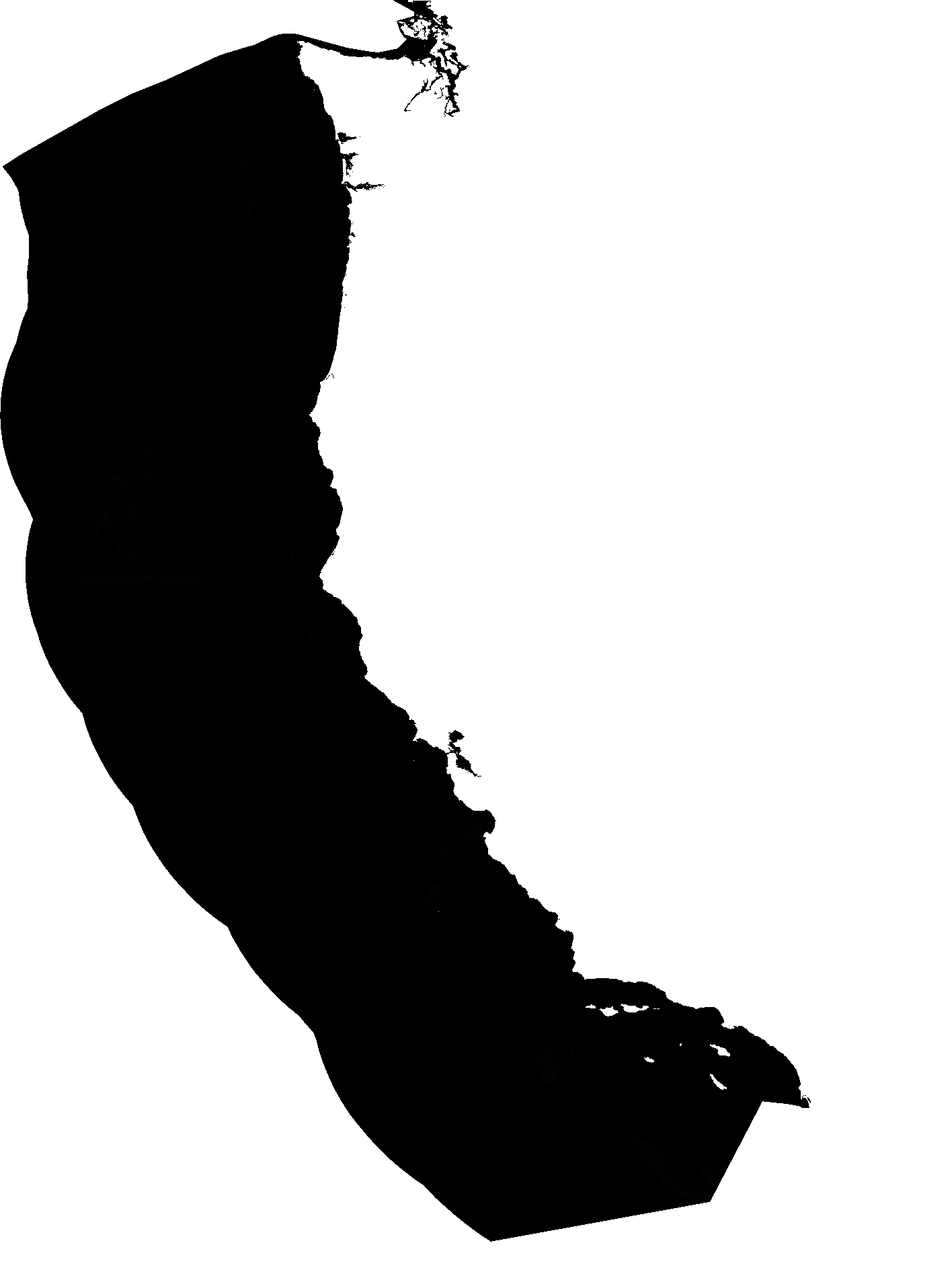

Supplement: File S2 — Model outputs for each taxa as ArcGIS GeoTIFF files with ArcGIS Map Documents and categorical layer files. (ZIP) [file pone.0093918.s009.zip › Antipatharia_Full_Extent/antipatharia_full_extent.tif.ovr]

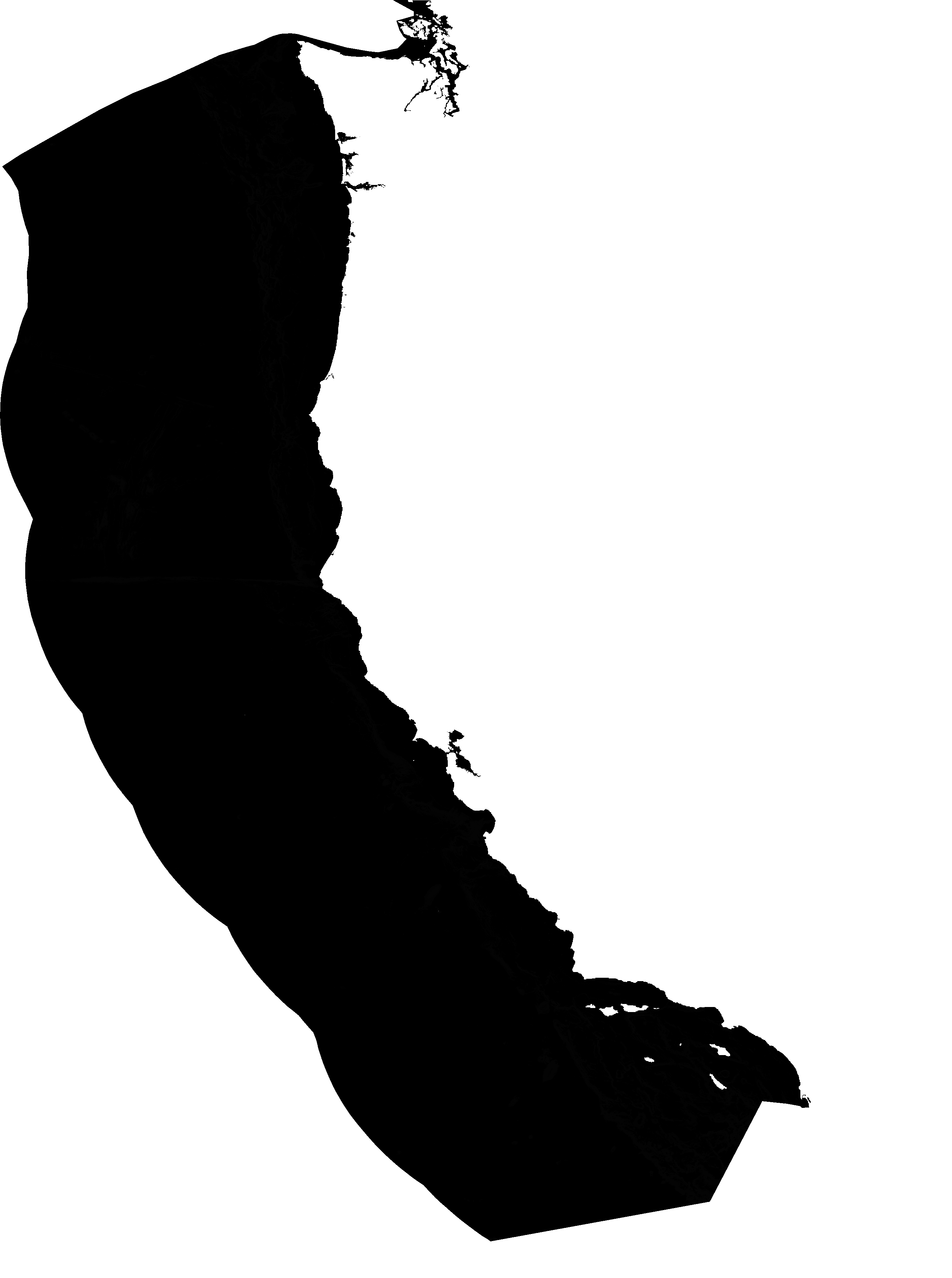

Supplement: File S2 — Model outputs for each taxa as ArcGIS GeoTIFF files with ArcGIS Map Documents and categorical layer files. (ZIP) [file pone.0093918.s009.zip › Calcaxonia_Full_Extent/calcaxonia_full_extent.tif]

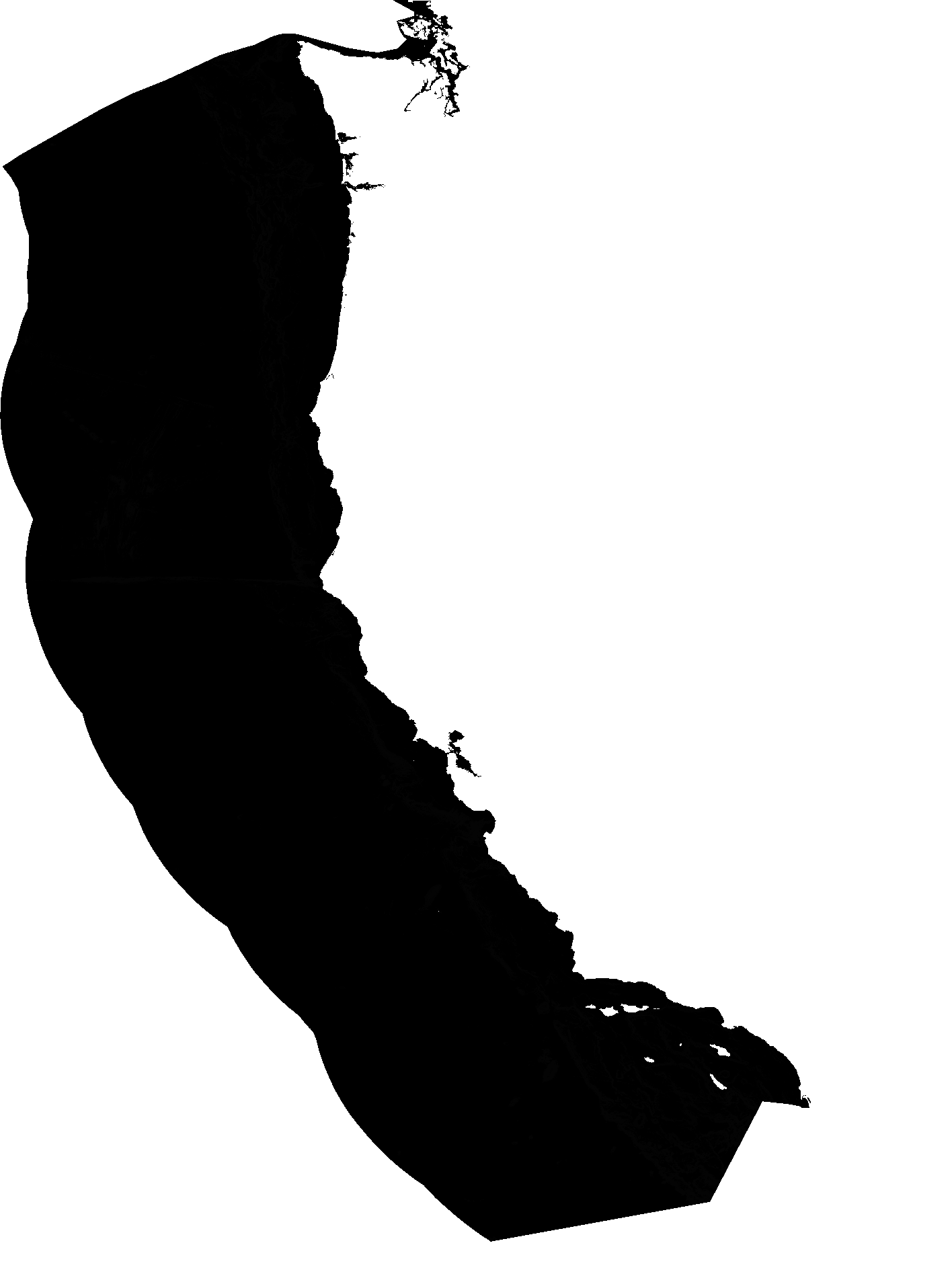

Supplement: File S2 — Model outputs for each taxa as ArcGIS GeoTIFF files with ArcGIS Map Documents and categorical layer files. (ZIP) [file pone.0093918.s009.zip › Calcaxonia_Full_Extent/calcaxonia_full_extent.tif.ovr]

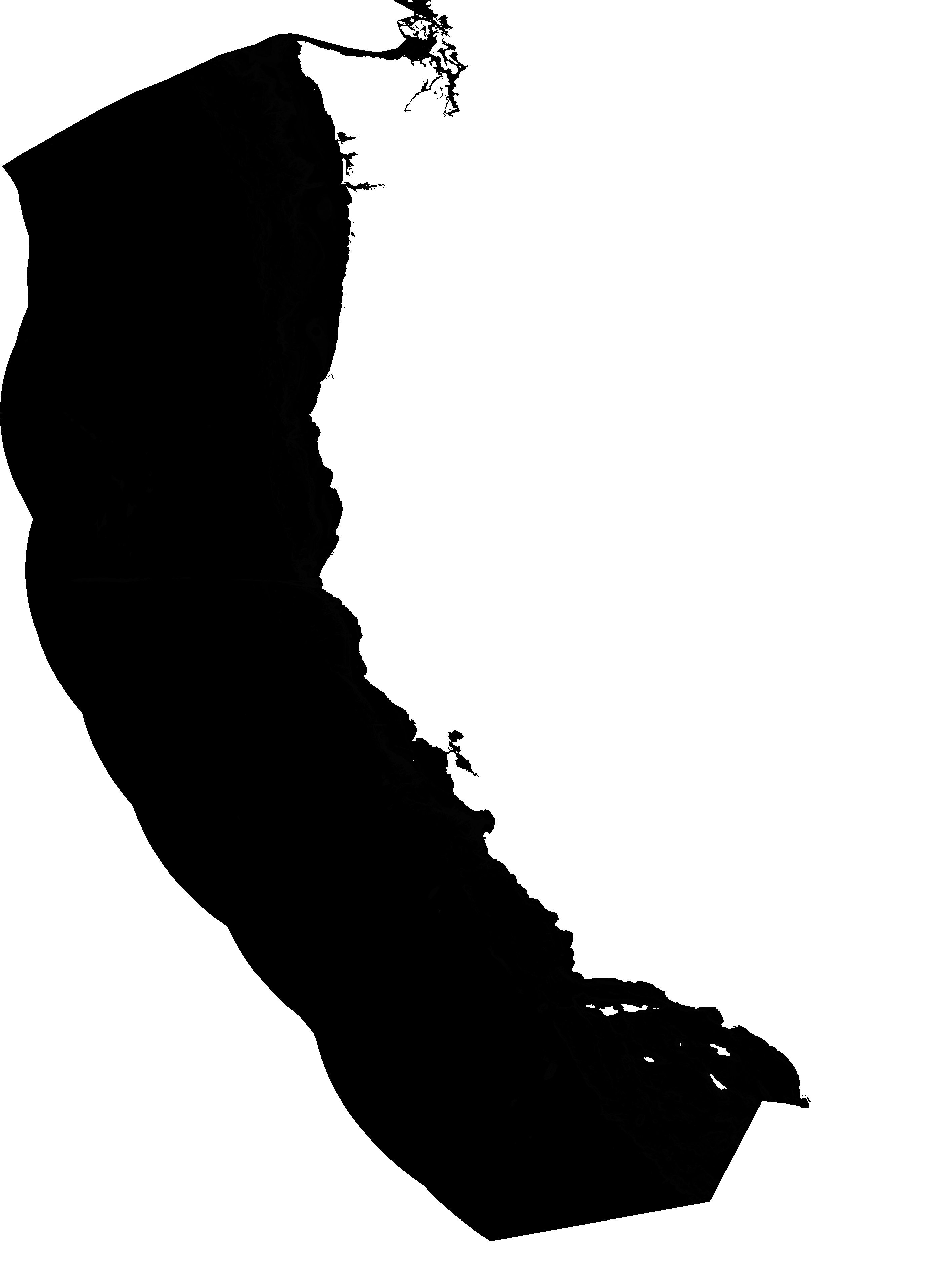

Supplement: File S2 — Model outputs for each taxa as ArcGIS GeoTIFF files with ArcGIS Map Documents and categorical layer files. (ZIP) [file pone.0093918.s009.zip › Holaxonia_Full_Extent/holaxonia_full_extent.tif]

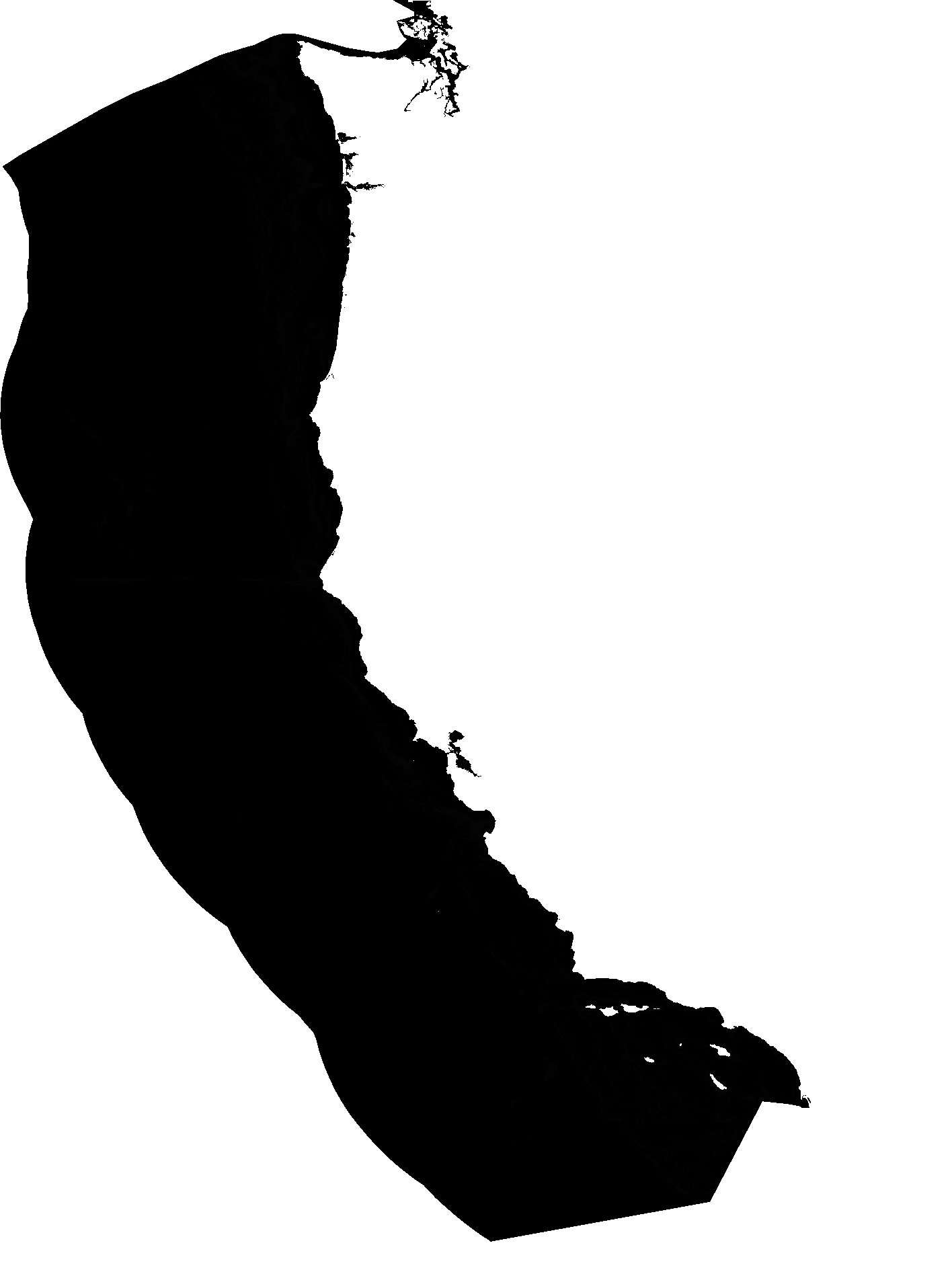

Supplement: File S2 — Model outputs for each taxa as ArcGIS GeoTIFF files with ArcGIS Map Documents and categorical layer files. (ZIP) [file pone.0093918.s009.zip › Holaxonia_Full_Extent/holaxonia_full_extent.tif.ovr]

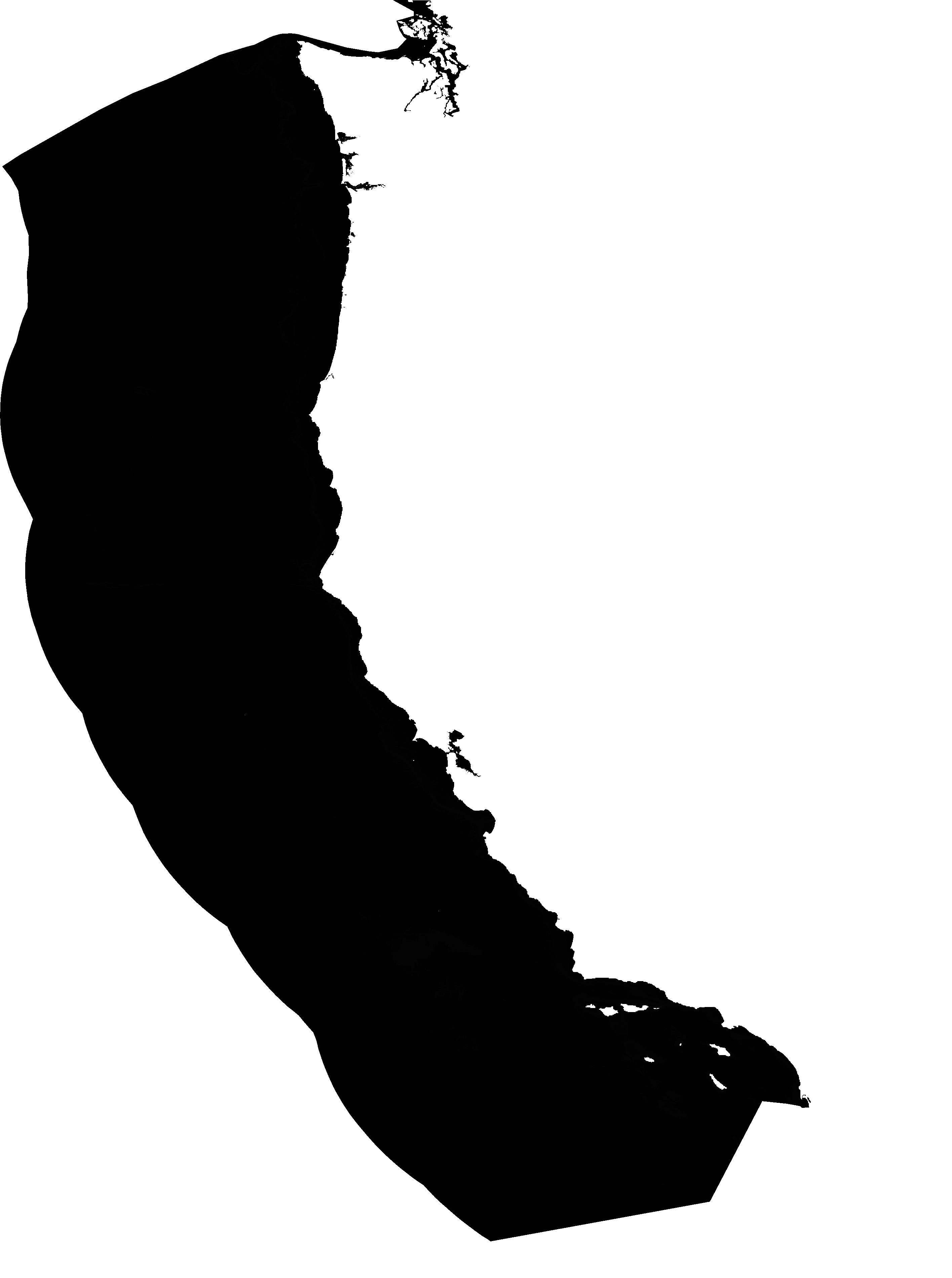

Supplement: File S2 — Model outputs for each taxa as ArcGIS GeoTIFF files with ArcGIS Map Documents and categorical layer files. (ZIP) [file pone.0093918.s009.zip › Scleractinia_Full_Extent/scleractinia_full_extent.tif]

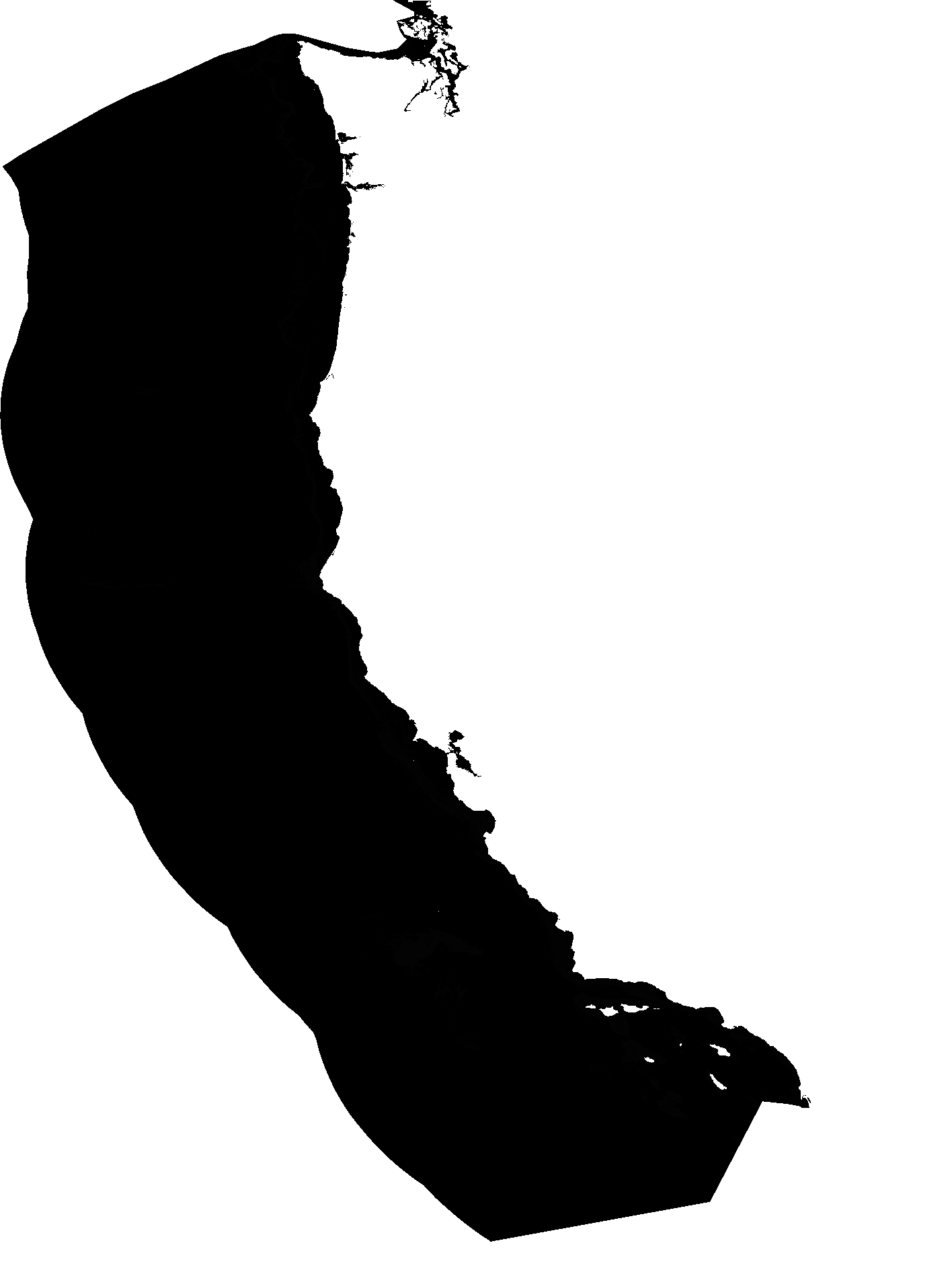

Supplement: File S2 — Model outputs for each taxa as ArcGIS GeoTIFF files with ArcGIS Map Documents and categorical layer files. (ZIP) [file pone.0093918.s009.zip › Scleractinia_Full_Extent/scleractinia_full_extent.tif.ovr]

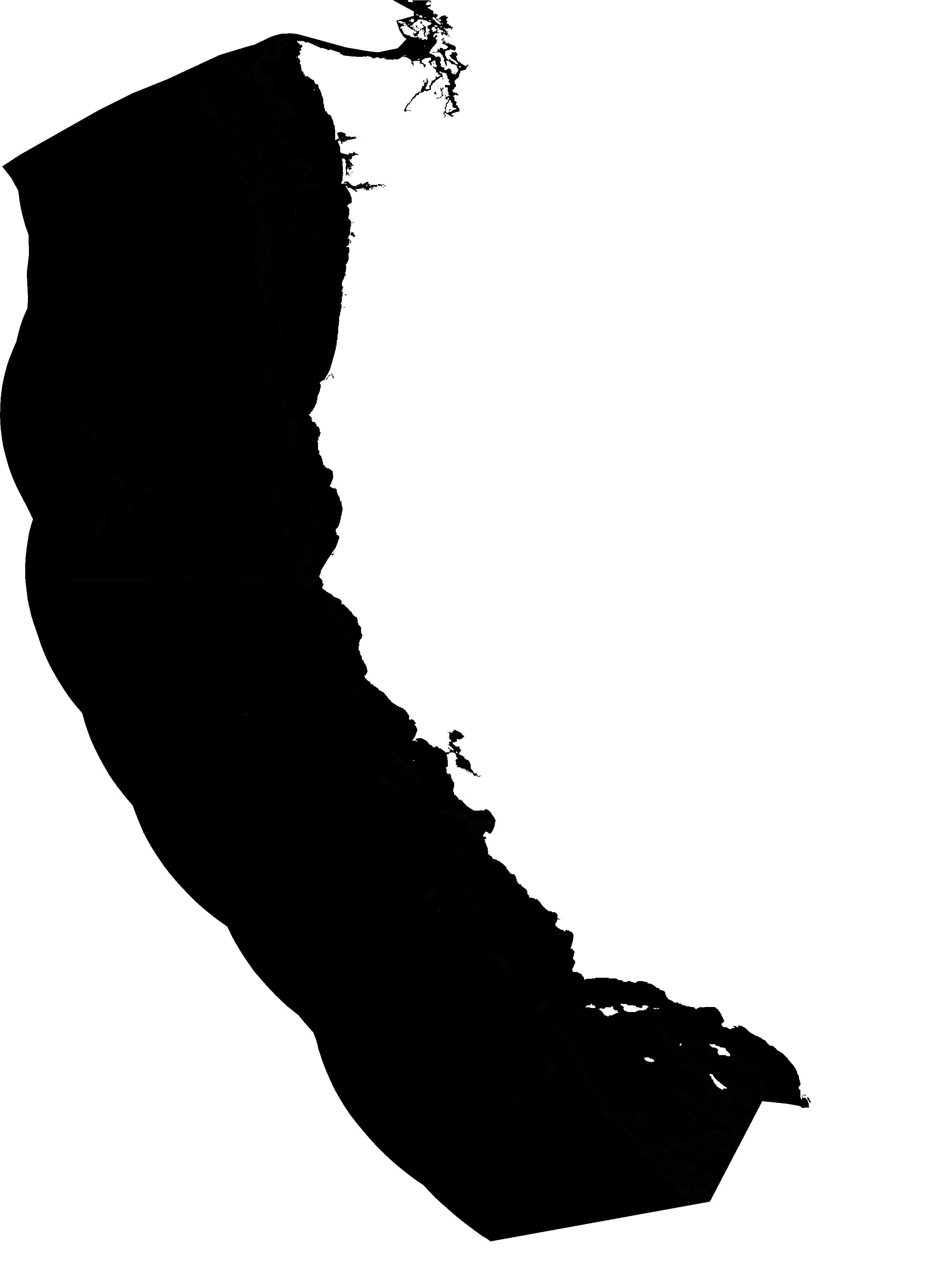

Supplement: File S2 — Model outputs for each taxa as ArcGIS GeoTIFF files with ArcGIS Map Documents and categorical layer files. (ZIP) [file pone.0093918.s009.zip › Scleraxonia_Full_Extent/scleraxonia_full_extent.tif]

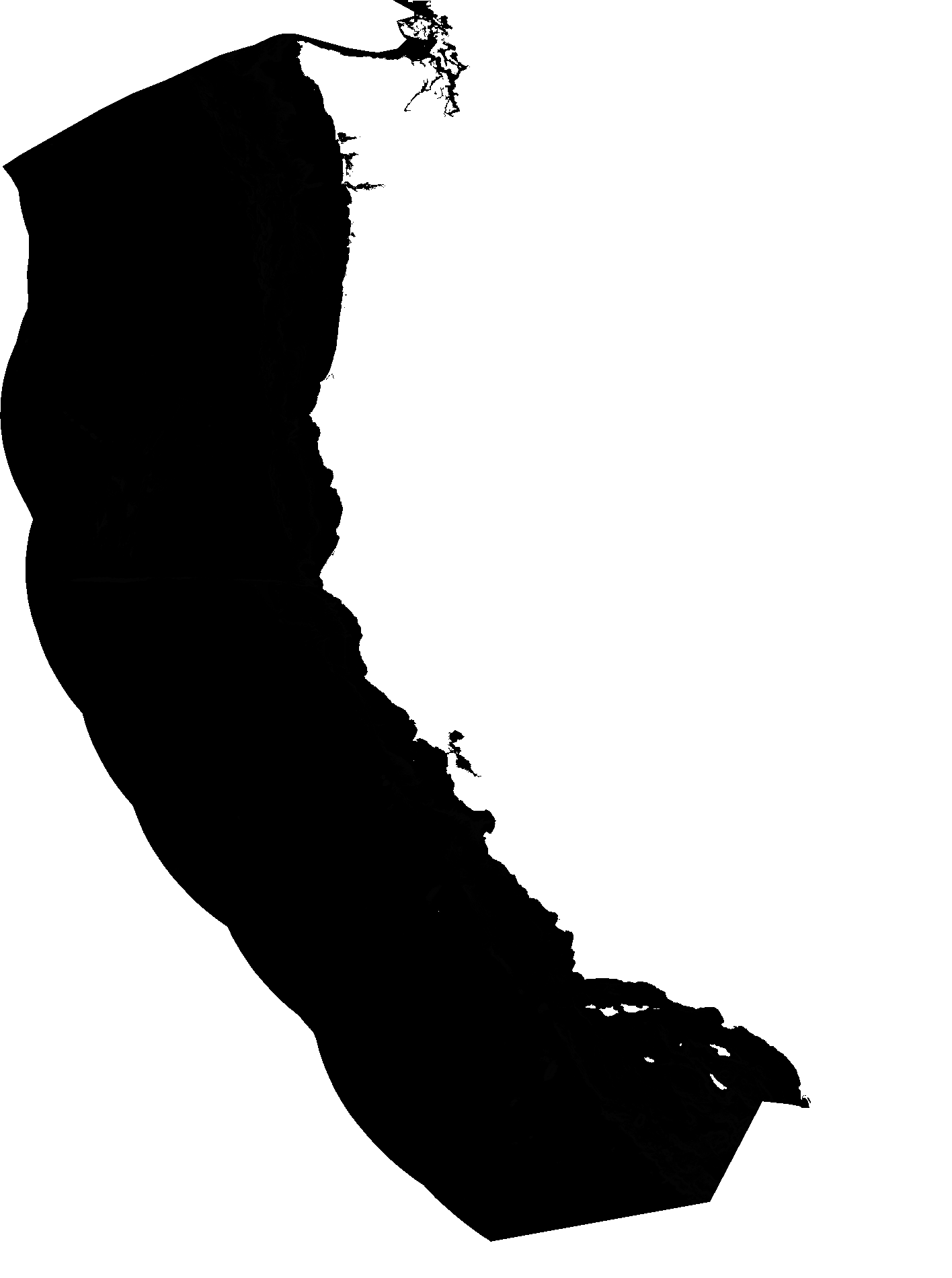

Supplement: File S2 — Model outputs for each taxa as ArcGIS GeoTIFF files with ArcGIS Map Documents and categorical layer files. (ZIP) [file pone.0093918.s009.zip › Scleraxonia_Full_Extent/scleraxonia_full_extent.tif.ovr]
