# Supplementary material for: Predicted Deep-Sea Coral Habitat Suitability for the U.S. West Coast
Source: PLoS One. 2014 Apr 23;9(4):e93918. doi: 10.1371/journal.pone.0093918 (PMC3997739; doi:10.1371/journal.pone.0093918)
Supplement: File S1 — Contains Tables S1–S10. Table S1: Correlation matrix for 10000 randomly placed points within the model domain. Table S2: Correlation matrix for points where the taxon Alcyoniina was found (n = 791). Table S3: Correlation matrix for points where the taxon Antipatharia was found (n = 128). Table S4: Correlation matrix for points where the taxon Calcaxonia was found (n = 413). Table S5: Correlation matrix for points where the taxon Filifera was found (n = 11). Table S6: Correlation matrix for points where the taxon Holaxonia was found (n = 308). Table S7: Correlation matrix for points where the taxon Scleractinia was found (n = 203). Table S8: Correlation matrix for points where the taxon Scleraxonia was found (n = 277). Table S9: Correlation matrix for points where the taxon Stolonifera was found (n = 30). Table S10: Correlation matrix for points where the taxon all species were found (n = 1059). (DOCX) [file pone.0093918.s008.docx]

**Table S1:** Correlation matrix for 10000 randomly placed points within the model domain.

|  | Depth | Dissolved O^2^ | MODIS | Nitrate | Omega A Orr | Omega C Orr | Phosphate | POC | POC DIVA | Salinity | Silicate | Slope 5km | Slope 10km | Slope 20km | Slope 2.5km | Slope 1km | Slope 500m | Omega A Stein | Omega C Stein |
| --- | --- | --- | --- | --- | --- | --- | --- | --- | --- | --- | --- | --- | --- | --- | --- | --- | --- | --- | --- |
|  |  |  |  |  |  |  |  |  |  |  |  |  |  |  |  |  |  |  |  |
| Dissolved O^2^ | -0.34 |  |  |  |  |  |  |  |  |  |  |  |  |  |  |  |  |  |  |
| MODIS | 0.58 | 0.34 |  |  |  |  |  |  |  |  |  |  |  |  |  |  |  |  |  |
| Nitrate | -0.36 | -0.74 | -0.73 |  |  |  |  |  |  |  |  |  |  |  |  |  |  |  |  |
| OmegaA Orr | 0.74 | 0.34 | 0.79 | -0.85 |  |  |  |  |  |  |  |  |  |  |  |  |  |  |  |
| OmegaC Orr | 0.75 | 0.32 | 0.79 | -0.84 | 1.00 |  |  |  |  |  |  |  |  |  |  |  |  |  |  |
| Phosphate | -0.16 | -0.86 | -0.63 | 0.97 | -0.74 | -0.72 |  |  |  |  |  |  |  |  |  |  |  |  |  |
| POC | 0.78 | 0.17 | 0.81 | -0.70 | 0.83 | 0.84 | -0.57 |  |  |  |  |  |  |  |  |  |  |  |  |
| POC DIVA | 0.79 | 0.18 | 0.83 | -0.71 | 0.84 | 0.85 | -0.58 | 0.98 |  |  |  |  |  |  |  |  |  |  |  |
| Salinity | -0.65 | -0.32 | -0.79 | 0.76 | -0.84 | -0.84 | 0.65 | -0.78 | -0.79 |  |  |  |  |  |  |  |  |  |  |
| Silicate | -0.83 | -0.14 | -0.74 | 0.73 | -0.84 | -0.85 | 0.58 | -0.92 | -0.92 | 0.81 |  |  |  |  |  |  |  |  |  |
| Slope 5km | 0.21 | -0.33 | -0.04 | 0.19 | -0.01 | -0.01 | 0.23 | 0.02 | 0.01 | 0.04 | -0.02 |  |  |  |  |  |  |  |  |
| Slope 10km | 0.26 | -0.37 | -0.02 | 0.21 | 0.00 | 0.01 | 0.26 | 0.04 | 0.04 | 0.03 | -0.05 | 0.90 |  |  |  |  |  |  |  |
| Slope 20km | 0.33 | -0.43 | 0.02 | 0.22 | 0.03 | 0.04 | 0.28 | 0.10 | 0.10 | 0.00 | -0.10 | 0.69 | 0.84 |  |  |  |  |  |  |
| Slope 2.5km | 0.17 | -0.30 | -0.06 | 0.19 | -0.03 | -0.02 | 0.22 | -0.01 | -0.02 | 0.06 | 0.02 | 0.93 | 0.80 | 0.61 |  |  |  |  |  |
| Slope 1km | 0.15 | -0.27 | -0.05 | 0.18 | -0.03 | -0.03 | 0.21 | -0.01 | -0.02 | 0.06 | 0.02 | 0.82 | 0.70 | 0.55 | 0.92 |  |  |  |  |
| Slope 500m | 0.14 | -0.27 | -0.05 | 0.18 | -0.04 | -0.03 | 0.21 | -0.01 | -0.02 | 0.06 | 0.03 | 0.77 | 0.66 | 0.53 | 0.86 | 0.94 |  |  |  |
| OmegaA Stein | 0.92 | 0.00 | 0.69 | -0.65 | 0.87 | 0.88 | -0.49 | 0.87 | 0.88 | -0.76 | -0.95 | 0.10 | 0.13 | 0.19 | 0.06 | 0.05 | 0.04 |  |  |
| OmegaC Stein | 0.92 | -0.01 | 0.69 | -0.65 | 0.87 | 0.88 | -0.48 | 0.87 | 0.88 | -0.76 | -0.95 | 0.10 | 0.13 | 0.19 | 0.06 | 0.05 | 0.04 | 1.00 |  |
| Temperature | 0.83 | 0.17 | 0.74 | -0.76 | 0.89 | 0.90 | -0.62 | 0.90 | 0.91 | -0.81 | -0.98 | 0.02 | 0.05 | 0.10 | -0.01 | -0.02 | -0.02 | 0.97 | 0.97 |

**Table S2:** Correlation matrix for points where the taxon Alcyoniina was found (n = 791).

|  | Depth | Dissolved O^2^ | MODIS | Nitrate | Omega A Orr | Omega C Orr | Phosphate | POC | POC DIVA | Salinity | Silicate | Slope 5km | Slope 10km | Slope 20km | Slope 2.5km | Slope 1km | Slope 500m | Omega A Stein | Omega C Stein |
| --- | --- | --- | --- | --- | --- | --- | --- | --- | --- | --- | --- | --- | --- | --- | --- | --- | --- | --- | --- |
|  |  |  |  |  |  |  |  |  |  |  |  |  |  |  |  |  |  |  |  |
| Dissolved O^2^ | -0.48 |  |  |  |  |  |  |  |  |  |  |  |  |  |  |  |  |  |  |
| MODIS | 0.57 | -0.18 |  |  |  |  |  |  |  |  |  |  |  |  |  |  |  |  |  |
| Nitrate | -0.33 | -0.65 | -0.33 |  |  |  |  |  |  |  |  |  |  |  |  |  |  |  |  |
| OmegaA Orr | 0.42 | 0.48 | 0.23 | -0.80 |  |  |  |  |  |  |  |  |  |  |  |  |  |  |  |
| OmegaC Orr | 0.44 | 0.46 | 0.24 | -0.80 | 1.00 |  |  |  |  |  |  |  |  |  |  |  |  |  |  |
| Phosphate | -0.07 | -0.83 | -0.16 | 0.94 | -0.77 | -0.76 |  |  |  |  |  |  |  |  |  |  |  |  |  |
| POC | 0.71 | -0.27 | 0.88 | -0.36 | 0.26 | 0.27 | -0.13 |  |  |  |  |  |  |  |  |  |  |  |  |
| POC DIVA | 0.72 | -0.26 | 0.88 | -0.38 | 0.27 | 0.29 | -0.15 | 0.98 |  |  |  |  |  |  |  |  |  |  |  |
| Salinity | -0.86 | 0.04 | -0.58 | 0.72 | -0.62 | -0.64 | 0.49 | -0.71 | -0.73 |  |  |  |  |  |  |  |  |  |  |
| Silicate | -0.89 | 0.13 | -0.61 | 0.66 | -0.54 | -0.55 | 0.42 | -0.74 | -0.76 | 0.98 |  |  |  |  |  |  |  |  |  |
| Slope 5km | 0.08 | -0.10 | -0.03 | 0.07 | 0.03 | 0.03 | 0.07 | 0.04 | 0.01 | 0.03 | 0.01 |  |  |  |  |  |  |  |  |
| Slope 10km | 0.10 | -0.14 | 0.03 | 0.09 | -0.03 | -0.02 | 0.12 | 0.16 | 0.13 | 0.01 | -0.02 | 0.75 |  |  |  |  |  |  |  |
| Slope 20km | 0.28 | -0.16 | 0.15 | -0.05 | 0.10 | 0.11 | 0.03 | 0.31 | 0.31 | -0.20 | -0.21 | 0.49 | 0.67 |  |  |  |  |  |  |
| Slope 2.5km | 0.08 | -0.11 | 0.02 | 0.09 | 0.02 | 0.02 | 0.10 | 0.06 | 0.03 | 0.04 | 0.02 | 0.84 | 0.58 | 0.38 |  |  |  |  |  |
| Slope 1km | 0.14 | -0.14 | 0.19 | 0.05 | 0.01 | 0.01 | 0.09 | 0.25 | 0.22 | -0.04 | -0.07 | 0.71 | 0.54 | 0.37 | 0.86 |  |  |  |  |
| Slope 500m | 0.14 | -0.16 | 0.22 | 0.07 | -0.01 | -0.01 | 0.11 | 0.27 | 0.25 | -0.03 | -0.06 | 0.62 | 0.50 | 0.34 | 0.66 | 0.85 |  |  |  |
| OmegaA Stein | 0.93 | -0.18 | 0.59 | -0.62 | 0.57 | 0.59 | -0.38 | 0.73 | 0.74 | -0.98 | -0.98 | 0.02 | 0.04 | 0.24 | 0.01 | 0.08 | 0.07 |  |  |
| OmegaC Stein | 0.94 | -0.19 | 0.59 | -0.61 | 0.57 | 0.58 | -0.37 | 0.73 | 0.75 | -0.98 | -0.98 | 0.02 | 0.04 | 0.24 | 0.01 | 0.08 | 0.07 | 1.00 |  |
| Temperature | 0.87 | -0.05 | 0.60 | -0.72 | 0.62 | 0.63 | -0.49 | 0.72 | 0.74 | -0.98 | -0.99 | 0.01 | 0.03 | 0.22 | 0.00 | 0.08 | 0.07 | 0.98 | 0.98 |

**Table S3:** Correlation matrix for points where the taxon Antipatharia was found (n = 128).

|  | Depth | Dissolved O^2^ | MODIS | Nitrate | Omega A Orr | Omega C Orr | Phosphate | POC | POC DIVA | Salinity | Silicate | Slope 5km | Slope 10km | Slope 20km | Slope 2.5km | Slope 1km | Slope 500m | Omega A Stein | Omega C Stein |
| --- | --- | --- | --- | --- | --- | --- | --- | --- | --- | --- | --- | --- | --- | --- | --- | --- | --- | --- | --- |
|  |  |  |  |  |  |  |  |  |  |  |  |  |  |  |  |  |  |  |  |
| Dissolved O^2^ | -0.89 |  |  |  |  |  |  |  |  |  |  |  |  |  |  |  |  |  |  |
| MODIS | 0.41 | -0.31 |  |  |  |  |  |  |  |  |  |  |  |  |  |  |  |  |  |
| Nitrate | 0.52 | -0.80 | 0.01 |  |  |  |  |  |  |  |  |  |  |  |  |  |  |  |  |
| OmegaA Orr | 0.74 | -0.50 | 0.15 | 0.25 |  |  |  |  |  |  |  |  |  |  |  |  |  |  |  |
| OmegaC Orr | 0.76 | -0.53 | 0.16 | 0.29 | 1.00 |  |  |  |  |  |  |  |  |  |  |  |  |  |  |
| Phosphate | 0.69 | -0.92 | 0.17 | 0.94 | 0.30 | 0.33 |  |  |  |  |  |  |  |  |  |  |  |  |  |
| POC | 0.52 | -0.34 | 0.84 | -0.15 | 0.26 | 0.26 | 0.10 |  |  |  |  |  |  |  |  |  |  |  |  |
| POC DIVA | 0.46 | -0.28 | 0.86 | -0.22 | 0.16 | 0.16 | 0.03 | 0.96 |  |  |  |  |  |  |  |  |  |  |  |
| Salinity | -0.76 | 0.50 | -0.53 | 0.10 | -0.60 | -0.61 | -0.16 | -0.79 | -0.76 |  |  |  |  |  |  |  |  |  |  |
| Silicate | -0.76 | 0.55 | -0.56 | 0.04 | -0.49 | -0.49 | -0.24 | -0.78 | -0.78 | 0.97 |  |  |  |  |  |  |  |  |  |
| Slope 5km | 0.18 | -0.17 | 0.18 | 0.25 | 0.16 | 0.17 | 0.22 | 0.08 | 0.04 | 0.05 | 0.07 |  |  |  |  |  |  |  |  |
| Slope 10km | 0.16 | -0.12 | 0.11 | 0.20 | 0.13 | 0.15 | 0.15 | 0.07 | 0.04 | 0.04 | 0.09 | 0.90 |  |  |  |  |  |  |  |
| Slope 20km | 0.13 | -0.12 | 0.17 | 0.12 | 0.03 | 0.04 | 0.13 | 0.16 | 0.15 | -0.01 | -0.01 | 0.59 | 0.71 |  |  |  |  |  |  |
| Slope 2.5km | 0.26 | -0.28 | 0.24 | 0.34 | 0.20 | 0.21 | 0.32 | 0.11 | 0.07 | 0.00 | -0.01 | 0.87 | 0.77 | 0.54 |  |  |  |  |  |
| Slope 1km | 0.19 | -0.21 | 0.43 | 0.23 | 0.09 | 0.09 | 0.24 | 0.27 | 0.24 | -0.03 | -0.05 | 0.79 | 0.65 | 0.52 | 0.87 |  |  |  |  |
| Slope 500m | 0.11 | -0.12 | 0.42 | 0.15 | 0.02 | 0.03 | 0.14 | 0.26 | 0.26 | 0.01 | 0.01 | 0.71 | 0.62 | 0.54 | 0.74 | 0.85 |  |  |  |
| OmegaA Stein | 0.94 | -0.78 | 0.49 | 0.28 | 0.65 | 0.67 | 0.52 | 0.67 | 0.63 | -0.91 | -0.92 | 0.08 | 0.07 | 0.10 | 0.16 | 0.15 | 0.06 |  |  |
| OmegaC Stein | 0.95 | -0.78 | 0.49 | 0.28 | 0.65 | 0.67 | 0.52 | 0.66 | 0.63 | -0.90 | -0.92 | 0.08 | 0.07 | 0.10 | 0.16 | 0.15 | 0.06 | 1.00 |  |
| Temperature | 0.80 | -0.58 | 0.53 | 0.00 | 0.55 | 0.56 | 0.27 | 0.76 | 0.75 | -0.98 | -0.99 | -0.04 | -0.04 | 0.02 | 0.03 | 0.05 | 0.00 | 0.94 | 0.94 |

**Table S4:** Correlation matrix for points where the taxon Calcaxonia was found (n = 413).

|  | Depth | Dissolved O^2^ | MODIS | Nitrate | Omega A Orr | Omega C Orr | Phosphate | POC | POC DIVA | Salinity | Silicate | Slope 5km | Slope 10km | Slope 20km | Slope 2.5km | Slope 1km | Slope 500m | Omega A Stein | Omega C Stein |
| --- | --- | --- | --- | --- | --- | --- | --- | --- | --- | --- | --- | --- | --- | --- | --- | --- | --- | --- | --- |
|  |  |  |  |  |  |  |  |  |  |  |  |  |  |  |  |  |  |  |  |
| Dissolved O^2^ | -0.63 |  |  |  |  |  |  |  |  |  |  |  |  |  |  |  |  |  |  |
| MODIS | 0.42 | -0.35 |  |  |  |  |  |  |  |  |  |  |  |  |  |  |  |  |  |
| Nitrate | -0.09 | -0.67 | 0.05 |  |  |  |  |  |  |  |  |  |  |  |  |  |  |  |  |
| OmegaA Orr | 0.29 | 0.43 | -0.07 | -0.71 |  |  |  |  |  |  |  |  |  |  |  |  |  |  |  |
| OmegaC Orr | 0.31 | 0.40 | -0.05 | -0.69 | 1.00 |  |  |  |  |  |  |  |  |  |  |  |  |  |  |
| Phosphate | 0.25 | -0.89 | 0.22 | 0.89 | -0.67 | -0.65 |  |  |  |  |  |  |  |  |  |  |  |  |  |
| POC | 0.58 | -0.28 | 0.90 | -0.18 | 0.12 | 0.13 | 0.04 |  |  |  |  |  |  |  |  |  |  |  |  |
| POC DIVA | 0.58 | -0.28 | 0.90 | -0.18 | 0.10 | 0.11 | 0.05 | 0.98 |  |  |  |  |  |  |  |  |  |  |  |
| Salinity | -0.76 | 0.06 | -0.34 | 0.66 | -0.61 | -0.61 | 0.33 | -0.59 | -0.59 |  |  |  |  |  |  |  |  |  |  |
| Silicate | -0.89 | 0.36 | -0.39 | 0.42 | -0.33 | -0.34 | 0.05 | -0.60 | -0.61 | 0.90 |  |  |  |  |  |  |  |  |  |
| Slope 5km | -0.11 | -0.06 | -0.15 | 0.20 | -0.11 | -0.12 | 0.16 | -0.22 | -0.22 | 0.23 | 0.15 |  |  |  |  |  |  |  |  |
| Slope 10km | -0.13 | 0.02 | -0.07 | 0.13 | -0.09 | -0.09 | 0.07 | -0.09 | -0.09 | 0.21 | 0.16 | 0.75 |  |  |  |  |  |  |  |
| Slope 20km | 0.04 | -0.08 | 0.19 | 0.13 | 0.00 | 0.02 | 0.09 | 0.14 | 0.13 | 0.10 | 0.06 | 0.42 | 0.67 |  |  |  |  |  |  |
| Slope 2.5km | -0.08 | -0.10 | -0.12 | 0.24 | -0.10 | -0.11 | 0.20 | -0.20 | -0.20 | 0.21 | 0.15 | 0.77 | 0.54 | 0.27 |  |  |  |  |  |
| Slope 1km | -0.04 | -0.14 | 0.20 | 0.23 | -0.14 | -0.14 | 0.22 | 0.11 | 0.10 | 0.15 | 0.10 | 0.62 | 0.45 | 0.32 | 0.80 |  |  |  |  |
| Slope 500m | -0.03 | -0.13 | 0.31 | 0.21 | -0.15 | -0.14 | 0.20 | 0.22 | 0.21 | 0.13 | 0.09 | 0.48 | 0.42 | 0.41 | 0.53 | 0.80 |  |  |  |
| OmegaA Stein | 0.94 | -0.42 | 0.40 | -0.36 | 0.38 | 0.40 | -0.01 | 0.60 | 0.60 | -0.89 | -0.97 | -0.17 | -0.17 | -0.03 | -0.16 | -0.11 | -0.10 |  |  |
| OmegaC Stein | 0.94 | -0.43 | 0.40 | -0.35 | 0.38 | 0.39 | 0.00 | 0.60 | 0.60 | -0.89 | -0.97 | -0.17 | -0.17 | -0.03 | -0.16 | -0.11 | -0.10 | 1.00 |  |
| Temperature | 0.88 | -0.33 | 0.37 | -0.46 | 0.39 | 0.39 | -0.10 | 0.58 | 0.59 | -0.91 | -0.99 | -0.15 | -0.15 | -0.05 | -0.17 | -0.11 | -0.10 | 0.98 | 0.98 |

**Table S5:** Correlation matrix for points where the taxon Filifera was found (n = 11).

|  | Depth | Dissolved O^2^ | MODIS | Nitrate | Omega A Orr | Omega C Orr | Phosphate | POC | POC DIVA | Salinity | Silicate | Slope 5km | Slope 10km | Slope 20km | Slope 2.5km | Slope 1km | Slope 500m | Omega A Stein | Omega C Stein |
| --- | --- | --- | --- | --- | --- | --- | --- | --- | --- | --- | --- | --- | --- | --- | --- | --- | --- | --- | --- |
|  |  |  |  |  |  |  |  |  |  |  |  |  |  |  |  |  |  |  |  |
| Dissolved O^2^ | 0.99 |  |  |  |  |  |  |  |  |  |  |  |  |  |  |  |  |  |  |
| MODIS | 0.64 | 0.65 |  |  |  |  |  |  |  |  |  |  |  |  |  |  |  |  |  |
| Nitrate | -0.86 | -0.86 | -0.44 |  |  |  |  |  |  |  |  |  |  |  |  |  |  |  |  |
| OmegaA Orr | 0.86 | 0.86 | 0.44 | -1.00 |  |  |  |  |  |  |  |  |  |  |  |  |  |  |  |
| Omeg C Orr | 0.86 | 0.86 | 0.43 | -1.00 | 1.00 |  |  |  |  |  |  |  |  |  |  |  |  |  |  |
| Phosphate | -0.99 | -1.00 | -0.63 | 0.89 | -0.89 | -0.89 |  |  |  |  |  |  |  |  |  |  |  |  |  |
| POC | 0.76 | 0.77 | 0.36 | -0.39 | 0.40 | 0.40 | -0.74 |  |  |  |  |  |  |  |  |  |  |  |  |
| POC DIVA | 0.87 | 0.88 | 0.59 | -0.52 | 0.53 | 0.53 | -0.85 | 0.96 |  |  |  |  |  |  |  |  |  |  |  |
| Salinity | -0.97 | -0.98 | -0.68 | 0.76 | -0.76 | -0.76 | 0.97 | -0.85 | -0.95 |  |  |  |  |  |  |  |  |  |  |
| Silicate | -0.88 | -0.88 | -0.46 | 1.00 | -1.00 | -1.00 | 0.91 | -0.44 | -0.56 | 0.79 |  |  |  |  |  |  |  |  |  |
| Slope 5km | -0.43 | -0.46 | -0.44 | -0.01 | 0.00 | 0.00 | 0.41 | -0.81 | -0.78 | 0.60 | 0.04 |  |  |  |  |  |  |  |  |
| Slope 10km | -0.77 | -0.79 | -0.62 | 0.37 | -0.38 | -0.38 | 0.75 | -0.94 | -0.97 | 0.88 | 0.42 | 0.88 |  |  |  |  |  |  |  |
| Slope 20km | -0.88 | -0.89 | -0.58 | 0.54 | -0.54 | -0.54 | 0.86 | -0.96 | -1.00 | 0.95 | 0.58 | 0.76 | 0.96 |  |  |  |  |  |  |
| Slope 2.5km | -0.91 | -0.92 | -0.56 | 0.70 | -0.71 | -0.71 | 0.91 | -0.85 | -0.92 | 0.94 | 0.73 | 0.60 | 0.84 | 0.92 |  |  |  |  |  |
| Slope 1km | -0.60 | -0.59 | -0.04 | 0.30 | -0.31 | -0.31 | 0.56 | -0.88 | -0.77 | 0.65 | 0.34 | 0.71 | 0.73 | 0.78 | 0.73 |  |  |  |  |
| Slope 500m | -0.67 | -0.63 | -0.11 | 0.42 | -0.43 | -0.43 | 0.61 | -0.80 | -0.72 | 0.66 | 0.45 | 0.62 | 0.66 | 0.73 | 0.73 | 0.93 |  |  |  |
| OmegaA Stein | -0.87 | -0.88 | -0.66 | 0.52 | -0.53 | -0.53 | 0.85 | -0.93 | -1.00 | 0.95 | 0.56 | 0.78 | 0.98 | 0.99 | 0.91 | 0.72 | 0.68 |  |  |
| OmegaC Stein | -0.87 | -0.88 | -0.66 | 0.52 | -0.53 | -0.53 | 0.85 | -0.94 | -1.00 | 0.95 | 0.56 | 0.78 | 0.98 | 0.99 | 0.91 | 0.72 | 0.68 | 1.00 |  |
| Temperature | 0.83 | 0.83 | 0.41 | -1.00 | 1.00 | 1.00 | -0.87 | 0.35 | 0.47 | -0.72 | -0.99 | 0.06 | -0.32 | -0.49 | -0.67 | -0.27 | -0.39 | -0.47 | -0.48 |

**Table S6:** Correlation matrix for points where the taxon Holaxonia was found (n = 308).

|  | Depth | Dissolved O^2^ | MODIS | Nitrate | Omega A Orr | Omega C Orr | Phosphate | POC | POC DIVA | Salinity | Silicate | Slope 5km | Slope 10km | Slope 20km | Slope 2.5km | Slope 1km | Slope 500m | Omega A Stein | Omega C Stein |
| --- | --- | --- | --- | --- | --- | --- | --- | --- | --- | --- | --- | --- | --- | --- | --- | --- | --- | --- | --- |
|  |  |  |  |  |  |  |  |  |  |  |  |  |  |  |  |  |  |  |  |
| Dissolved O^2^ | 0.42 |  |  |  |  |  |  |  |  |  |  |  |  |  |  |  |  |  |  |
| MODIS | 0.30 | -0.06 |  |  |  |  |  |  |  |  |  |  |  |  |  |  |  |  |  |
| Nitrate | -0.68 | -0.94 | -0.09 |  |  |  |  |  |  |  |  |  |  |  |  |  |  |  |  |
| Omega A Orr | 0.57 | 0.96 | 0.01 | -0.96 |  |  |  |  |  |  |  |  |  |  |  |  |  |  |  |
| Omega C Orr | 0.57 | 0.96 | 0.01 | -0.96 | 1.00 |  |  |  |  |  |  |  |  |  |  |  |  |  |  |
| Phosphate | -0.60 | -0.96 | -0.02 | 0.98 | -0.96 | -0.96 |  |  |  |  |  |  |  |  |  |  |  |  |  |
| POC | 0.65 | 0.20 | 0.70 | -0.35 | 0.26 | 0.26 | -0.28 |  |  |  |  |  |  |  |  |  |  |  |  |
| POC DIVA | 0.66 | 0.22 | 0.73 | -0.39 | 0.29 | 0.29 | -0.32 | 0.97 |  |  |  |  |  |  |  |  |  |  |  |
| Salinity | -0.82 | -0.80 | -0.15 | 0.90 | -0.86 | -0.86 | 0.86 | -0.55 | -0.57 |  |  |  |  |  |  |  |  |  |  |
| Silicate | -0.95 | -0.63 | -0.26 | 0.84 | -0.73 | -0.73 | 0.78 | -0.60 | -0.62 | 0.91 |  |  |  |  |  |  |  |  |  |
| Slope 5km | -0.46 | -0.45 | -0.05 | 0.49 | -0.48 | -0.48 | 0.48 | -0.31 | -0.34 | 0.57 | 0.49 |  |  |  |  |  |  |  |  |
| Slope 10km | -0.42 | -0.42 | 0.08 | 0.45 | -0.46 | -0.46 | 0.45 | -0.17 | -0.20 | 0.54 | 0.44 | 0.74 |  |  |  |  |  |  |  |
| Slope 20km | -0.08 | -0.28 | 0.08 | 0.20 | -0.25 | -0.25 | 0.23 | -0.11 | -0.11 | 0.29 | 0.11 | 0.53 | 0.67 |  |  |  |  |  |  |
| Slope 2.5km | -0.47 | -0.47 | 0.02 | 0.52 | -0.48 | -0.49 | 0.51 | -0.26 | -0.29 | 0.58 | 0.52 | 0.83 | 0.62 | 0.41 |  |  |  |  |  |
| Slope 1km | -0.39 | -0.47 | 0.20 | 0.49 | -0.47 | -0.48 | 0.50 | -0.06 | -0.08 | 0.52 | 0.44 | 0.72 | 0.58 | 0.40 | 0.87 |  |  |  |  |
| Slope 500m | -0.32 | -0.48 | 0.28 | 0.47 | -0.47 | -0.47 | 0.49 | 0.03 | 0.02 | 0.49 | 0.39 | 0.64 | 0.58 | 0.42 | 0.72 | 0.89 |  |  |  |
| OmegaA Stein | 0.89 | 0.66 | 0.21 | -0.87 | 0.78 | 0.78 | -0.82 | 0.47 | 0.50 | -0.86 | -0.95 | -0.49 | -0.43 | -0.09 | -0.52 | -0.46 | -0.41 |  |  |
| OmegaC Stein | 0.90 | 0.66 | 0.21 | -0.87 | 0.77 | 0.78 | -0.82 | 0.48 | 0.51 | -0.86 | -0.96 | -0.49 | -0.43 | -0.09 | -0.52 | -0.46 | -0.41 | 1.00 |  |
| Temperature | 0.90 | 0.71 | 0.24 | -0.90 | 0.81 | 0.81 | -0.85 | 0.51 | 0.54 | -0.89 | -0.98 | -0.47 | -0.41 | -0.09 | -0.51 | -0.44 | -0.39 | 0.98 | 0.98 |

**Table S7:** Correlation matrix for points where the taxon Scleractinia was found (n = 203).

|  | Depth | Dissolved O^2^ | MODIS | Nitrate | Omega A Orr | Omega C Orr | Phosphate | POC | POC DIVA | Salinity | Silicate | Slope 5km | Slope 10km | Slope 20km | Slope 2.5km | Slope 1km | Slope 500m | Omega A Stein | Omega C Stein |
| --- | --- | --- | --- | --- | --- | --- | --- | --- | --- | --- | --- | --- | --- | --- | --- | --- | --- | --- | --- |
|  |  |  |  |  |  |  |  |  |  |  |  |  |  |  |  |  |  |  |  |
| Dissolved O^2^ | -0.01 |  |  |  |  |  |  |  |  |  |  |  |  |  |  |  |  |  |  |
| MODIS | 0.44 | -0.30 |  |  |  |  |  |  |  |  |  |  |  |  |  |  |  |  |  |
| Nitrate | -0.50 | -0.85 | 0.00 |  |  |  |  |  |  |  |  |  |  |  |  |  |  |  |  |
| OmegaA Orr | 0.64 | 0.74 | 0.05 | -0.96 |  |  |  |  |  |  |  |  |  |  |  |  |  |  |  |
| OmegaC Orr | 0.65 | 0.73 | 0.06 | -0.96 | 1.00 |  |  |  |  |  |  |  |  |  |  |  |  |  |  |
| Phosphate | -0.40 | -0.90 | 0.08 | 0.99 | -0.93 | -0.93 |  |  |  |  |  |  |  |  |  |  |  |  |  |
| POC | 0.71 | -0.27 | 0.80 | -0.14 | 0.23 | 0.24 | -0.04 |  |  |  |  |  |  |  |  |  |  |  |  |
| POC DIVA | 0.74 | -0.20 | 0.79 | -0.22 | 0.31 | 0.32 | -0.12 | 0.97 |  |  |  |  |  |  |  |  |  |  |  |
| Salinity | -0.81 | -0.53 | -0.22 | 0.87 | -0.91 | -0.92 | 0.81 | -0.48 | -0.54 |  |  |  |  |  |  |  |  |  |  |
| Silicate | -0.92 | -0.32 | -0.34 | 0.76 | -0.81 | -0.82 | 0.69 | -0.59 | -0.64 | 0.93 |  |  |  |  |  |  |  |  |  |
| Slope 5km | 0.26 | -0.42 | 0.25 | 0.22 | -0.11 | -0.10 | 0.27 | 0.27 | 0.25 | 0.06 | -0.07 |  |  |  |  |  |  |  |  |
| Slope 10km | 0.34 | -0.42 | 0.28 | 0.18 | -0.05 | -0.04 | 0.24 | 0.28 | 0.27 | 0.00 | -0.12 | 0.91 |  |  |  |  |  |  |  |
| Slope 20km | 0.53 | -0.16 | 0.15 | -0.16 | 0.28 | 0.29 | -0.08 | 0.25 | 0.25 | -0.30 | -0.41 | 0.71 | 0.83 |  |  |  |  |  |  |
| Slope 2.5km | 0.23 | -0.42 | 0.28 | 0.24 | -0.14 | -0.13 | 0.28 | 0.29 | 0.28 | 0.07 | -0.05 | 0.94 | 0.82 | 0.62 |  |  |  |  |  |
| Slope 1km | 0.25 | -0.52 | 0.36 | 0.32 | -0.21 | -0.20 | 0.37 | 0.38 | 0.36 | 0.11 | -0.03 | 0.86 | 0.78 | 0.60 | 0.93 |  |  |  |  |
| Slope 500m | 0.23 | -0.62 | 0.39 | 0.42 | -0.29 | -0.28 | 0.47 | 0.41 | 0.38 | 0.19 | 0.03 | 0.79 | 0.75 | 0.56 | 0.80 | 0.91 |  |  |  |
| OmegaA Stein | 0.88 | 0.41 | 0.26 | -0.81 | 0.88 | 0.89 | -0.75 | 0.50 | 0.56 | -0.93 | -0.96 | 0.06 | 0.12 | 0.40 | 0.03 | -0.01 | -0.06 |  |  |
| OmegaC Stein | 0.89 | 0.40 | 0.27 | -0.81 | 0.88 | 0.88 | -0.74 | 0.51 | 0.57 | -0.93 | -0.97 | 0.07 | 0.12 | 0.41 | 0.03 | 0.00 | -0.05 | 1.00 |  |
| Temperature | 0.87 | 0.44 | 0.27 | -0.84 | 0.89 | 0.89 | -0.78 | 0.51 | 0.57 | -0.94 | -0.98 | 0.03 | 0.09 | 0.39 | 0.01 | -0.02 | -0.09 | 0.98 | 0.98 |

**Table S8:** Correlation matrix for points where the taxon Scleraxonia was found (n = 277).

|  | Depth | Dissolved O^2^ | MODIS | Nitrate | Omega A Orr | Omega C Orr | Phosphate | POC | POC DIVA | Salinity | Silicate | Slope 5km | Slope 10km | Slope 20km | Slope 2.5km | Slope 1km | Slope 500m | Omega A Stein | Omega C Stein |
| --- | --- | --- | --- | --- | --- | --- | --- | --- | --- | --- | --- | --- | --- | --- | --- | --- | --- | --- | --- |
|  |  |  |  |  |  |  |  |  |  |  |  |  |  |  |  |  |  |  |  |
| Dissolved O^2^ | -0.49 |  |  |  |  |  |  |  |  |  |  |  |  |  |  |  |  |  |  |
| MODIS | 0.43 | -0.30 |  |  |  |  |  |  |  |  |  |  |  |  |  |  |  |  |  |
| Nitrate | -0.36 | -0.58 | -0.10 |  |  |  |  |  |  |  |  |  |  |  |  |  |  |  |  |
| OmegaA Orr | 0.10 | 0.62 | -0.09 | -0.66 |  |  |  |  |  |  |  |  |  |  |  |  |  |  |  |
| OmegaC Orr | 0.12 | 0.61 | -0.09 | -0.66 | 1.00 |  |  |  |  |  |  |  |  |  |  |  |  |  |  |
| Phosphate | 0.02 | -0.85 | 0.13 | 0.85 | -0.74 | -0.74 |  |  |  |  |  |  |  |  |  |  |  |  |  |
| POC | 0.62 | -0.12 | 0.87 | -0.42 | 0.14 | 0.15 | -0.17 |  |  |  |  |  |  |  |  |  |  |  |  |
| POC DIVA | 0.63 | -0.12 | 0.88 | -0.43 | 0.12 | 0.13 | -0.17 | 0.98 |  |  |  |  |  |  |  |  |  |  |  |
| Salinity | -0.86 | 0.04 | -0.38 | 0.74 | -0.38 | -0.40 | 0.42 | -0.69 | -0.69 |  |  |  |  |  |  |  |  |  |  |
| Silicate | -0.94 | 0.28 | -0.42 | 0.59 | -0.18 | -0.20 | 0.20 | -0.66 | -0.67 | 0.95 |  |  |  |  |  |  |  |  |  |
| Slope 5km | -0.30 | -0.08 | 0.02 | 0.30 | -0.22 | -0.22 | 0.26 | -0.16 | -0.15 | 0.42 | 0.33 |  |  |  |  |  |  |  |  |
| Slope 10km | -0.31 | -0.07 | 0.15 | 0.28 | -0.29 | -0.29 | 0.25 | -0.04 | -0.01 | 0.40 | 0.32 | 0.76 |  |  |  |  |  |  |  |
| Slope 20km | -0.12 | -0.20 | 0.30 | 0.25 | -0.28 | -0.28 | 0.27 | 0.09 | 0.13 | 0.23 | 0.16 | 0.54 | 0.70 |  |  |  |  |  |  |
| Slope 2.5km | -0.26 | -0.10 | -0.03 | 0.30 | -0.19 | -0.19 | 0.26 | -0.21 | -0.20 | 0.39 | 0.30 | 0.81 | 0.61 | 0.44 |  |  |  |  |  |
| Slope 1km | -0.14 | -0.15 | 0.22 | 0.24 | -0.22 | -0.23 | 0.26 | 0.06 | 0.06 | 0.26 | 0.17 | 0.72 | 0.59 | 0.47 | 0.83 |  |  |  |  |
| Slope 500m | -0.12 | -0.15 | 0.34 | 0.20 | -0.25 | -0.25 | 0.25 | 0.18 | 0.18 | 0.21 | 0.13 | 0.62 | 0.59 | 0.50 | 0.62 | 0.84 |  |  |  |
| OmegaA Stein | 0.96 | -0.33 | 0.43 | -0.53 | 0.15 | 0.16 | -0.16 | 0.66 | 0.67 | -0.93 | -0.98 | -0.35 | -0.33 | -0.14 | -0.32 | -0.19 | -0.16 |  |  |
| OmegaC Stein | 0.96 | -0.34 | 0.43 | -0.53 | 0.14 | 0.16 | -0.16 | 0.66 | 0.67 | -0.93 | -0.98 | -0.35 | -0.33 | -0.13 | -0.32 | -0.19 | -0.16 | 1.00 |  |
| Temperature | 0.93 | -0.26 | 0.42 | -0.62 | 0.22 | 0.23 | -0.23 | 0.65 | 0.66 | -0.94 | -0.99 | -0.31 | -0.29 | -0.16 | -0.28 | -0.15 | -0.11 | 0.97 | 0.97 |

**Table S9:** Correlation matrix for points where the taxon Stolonifera was found (n = 30).

|  | Depth | Dissolved O^2^ | MODIS | Nitrate | Omega A Orr | Omega C Orr | Phosphate | POC | POC DIVA | Salinity | Silicate | Slope 5km | Slope 10km | Slope 20km | Slope 2.5km | Slope 1km | Slope 500m | Omega A Stein | Omega C Stein |
| --- | --- | --- | --- | --- | --- | --- | --- | --- | --- | --- | --- | --- | --- | --- | --- | --- | --- | --- | --- |
|  |  |  |  |  |  |  |  |  |  |  |  |  |  |  |  |  |  |  |  |
| Dissolved O^2^ | 0.33 |  |  |  |  |  |  |  |  |  |  |  |  |  |  |  |  |  |  |
| MODIS | 0.14 | 0.01 |  |  |  |  |  |  |  |  |  |  |  |  |  |  |  |  |  |
| Nitrate | -0.60 | -0.94 | -0.06 |  |  |  |  |  |  |  |  |  |  |  |  |  |  |  |  |
| OmegaA Orr | 0.61 | 0.94 | 0.06 | -0.97 |  |  |  |  |  |  |  |  |  |  |  |  |  |  |  |
| OmegaC Orr | 0.61 | 0.94 | 0.07 | -0.97 | 1.00 |  |  |  |  |  |  |  |  |  |  |  |  |  |  |
| Phosphate | -0.52 | -0.98 | -0.06 | 0.99 | -0.98 | -0.98 |  |  |  |  |  |  |  |  |  |  |  |  |  |
| POC | 0.46 | 0.43 | 0.80 | -0.53 | 0.50 | 0.50 | -0.51 |  |  |  |  |  |  |  |  |  |  |  |  |
| POC DIVA | 0.41 | 0.39 | 0.85 | -0.48 | 0.44 | 0.45 | -0.47 | 0.97 |  |  |  |  |  |  |  |  |  |  |  |
| Salinity | -0.81 | -0.80 | -0.14 | 0.95 | -0.93 | -0.93 | 0.91 | -0.58 | -0.53 |  |  |  |  |  |  |  |  |  |  |
| Silicate | -0.89 | -0.65 | -0.11 | 0.85 | -0.81 | -0.81 | 0.79 | -0.53 | -0.49 | 0.97 |  |  |  |  |  |  |  |  |  |
| Slope 5km | -0.20 | -0.42 | 0.13 | 0.46 | -0.36 | -0.36 | 0.44 | -0.17 | -0.12 | 0.42 | 0.40 |  |  |  |  |  |  |  |  |
| Slope 10km | -0.09 | -0.45 | 0.31 | 0.43 | -0.38 | -0.38 | 0.43 | 0.10 | 0.14 | 0.34 | 0.28 | 0.78 |  |  |  |  |  |  |  |
| Slope 20km | -0.33 | -0.24 | 0.32 | 0.33 | -0.30 | -0.30 | 0.29 | 0.09 | 0.17 | 0.34 | 0.36 | 0.64 | 0.67 |  |  |  |  |  |  |
| Slope 2.5km | -0.29 | -0.39 | 0.11 | 0.47 | -0.35 | -0.34 | 0.43 | -0.30 | -0.24 | 0.45 | 0.48 | 0.84 | 0.58 | 0.39 |  |  |  |  |  |
| Slope 1km | -0.28 | -0.30 | 0.16 | 0.40 | -0.28 | -0.27 | 0.35 | -0.26 | -0.19 | 0.39 | 0.43 | 0.75 | 0.45 | 0.36 | 0.91 |  |  |  |  |
| Slope 500m | -0.27 | -0.32 | 0.48 | 0.38 | -0.31 | -0.31 | 0.35 | 0.07 | 0.13 | 0.37 | 0.41 | 0.59 | 0.46 | 0.49 | 0.62 | 0.71 |  |  |  |
| OmegaA Stein | 0.83 | 0.79 | 0.13 | -0.94 | 0.92 | 0.93 | -0.90 | 0.57 | 0.52 | -1.00 | -0.96 | -0.41 | -0.34 | -0.36 | -0.44 | -0.38 | -0.36 |  |  |
| OmegaC Stein | 0.83 | 0.78 | 0.13 | -0.94 | 0.92 | 0.92 | -0.90 | 0.57 | 0.52 | -0.99 | -0.97 | -0.40 | -0.33 | -0.36 | -0.44 | -0.38 | -0.36 | 1.00 |  |
| Temperature | 0.84 | 0.76 | 0.11 | -0.93 | 0.90 | 0.90 | -0.88 | 0.55 | 0.51 | -1.00 | -0.98 | -0.42 | -0.33 | -0.37 | -0.47 | -0.41 | -0.39 | 0.99 | 0.99 |

**Table S10:** Correlation matrix for points where the taxon all species were found (n = 1059).

|  | Depth | Dissolved O^2^ | MODIS | Nitrate | Omega A Orr | Omega C Orr | Phosphate | POC | POC DIVA | Salinity | Silicate | Slope 5km | Slope 10km | Slope 20km | Slope 2.5km | Slope 1km | Slope 500m | Omega A Stein | Omega C Stein |
| --- | --- | --- | --- | --- | --- | --- | --- | --- | --- | --- | --- | --- | --- | --- | --- | --- | --- | --- | --- |
|  |  |  |  |  |  |  |  |  |  |  |  |  |  |  |  |  |  |  |  |
| Dissolved O^2^ | -0.11 |  |  |  |  |  |  |  |  |  |  |  |  |  |  |  |  |  |  |
| MODIS | 0.48 | -0.18 |  |  |  |  |  |  |  |  |  |  |  |  |  |  |  |  |  |
| Nitrate | -0.42 | -0.84 | -0.13 |  |  |  |  |  |  |  |  |  |  |  |  |  |  |  |  |
| OmegaA Orr | 0.52 | 0.74 | 0.09 | -0.92 |  |  |  |  |  |  |  |  |  |  |  |  |  |  |  |
| OmegaC Orr | 0.53 | 0.73 | 0.10 | -0.92 | 1.00 |  |  |  |  |  |  |  |  |  |  |  |  |  |  |
| Phosphate | -0.28 | -0.91 | -0.04 | 0.98 | -0.90 | -0.89 |  |  |  |  |  |  |  |  |  |  |  |  |  |
| POC | 0.70 | -0.06 | 0.81 | -0.32 | 0.29 | 0.29 | -0.20 |  |  |  |  |  |  |  |  |  |  |  |  |
| POC DIVA | 0.70 | -0.04 | 0.82 | -0.35 | 0.32 | 0.32 | -0.23 | 0.98 |  |  |  |  |  |  |  |  |  |  |  |
| Salinity | -0.78 | -0.48 | -0.33 | 0.84 | -0.83 | -0.84 | 0.75 | -0.62 | -0.64 |  |  |  |  |  |  |  |  |  |  |
| Silicate | -0.87 | -0.28 | -0.46 | 0.74 | -0.69 | -0.70 | 0.62 | -0.69 | -0.71 | 0.93 |  |  |  |  |  |  |  |  |  |
| Slope 5km | 0.01 | -0.36 | 0.02 | 0.33 | -0.23 | -0.23 | 0.34 | -0.07 | -0.09 | 0.28 | 0.19 |  |  |  |  |  |  |  |  |
| Slope 10km | 0.05 | -0.37 | 0.06 | 0.31 | -0.22 | -0.21 | 0.34 | 0.00 | -0.01 | 0.25 | 0.16 | 0.81 |  |  |  |  |  |  |  |
| Slope 20km | 0.23 | -0.28 | 0.13 | 0.11 | -0.03 | -0.03 | 0.17 | 0.13 | 0.13 | 0.03 | -0.07 | 0.57 | 0.74 |  |  |  |  |  |  |
| Slope 2.5km | 0.00 | -0.37 | 0.06 | 0.35 | -0.25 | -0.24 | 0.35 | -0.05 | -0.07 | 0.29 | 0.21 | 0.87 | 0.67 | 0.47 |  |  |  |  |  |
| Slope 1km | 0.04 | -0.37 | 0.20 | 0.32 | -0.24 | -0.24 | 0.34 | 0.10 | 0.09 | 0.24 | 0.14 | 0.76 | 0.62 | 0.45 | 0.88 |  |  |  |  |
| Slope 500m | 0.05 | -0.39 | 0.24 | 0.32 | -0.26 | -0.25 | 0.35 | 0.14 | 0.13 | 0.24 | 0.14 | 0.69 | 0.58 | 0.43 | 0.73 | 0.88 |  |  |  |
| OmegaA Stein | 0.88 | 0.31 | 0.40 | -0.76 | 0.77 | 0.78 | -0.66 | 0.62 | 0.64 | -0.91 | -0.96 | -0.16 | -0.13 | 0.11 | -0.18 | -0.13 | -0.13 |  |  |
| OmegaC Stein | 0.88 | 0.30 | 0.41 | -0.76 | 0.77 | 0.77 | -0.65 | 0.62 | 0.65 | -0.92 | -0.96 | -0.16 | -0.13 | 0.11 | -0.18 | -0.13 | -0.12 | 1.00 |  |
| Temperature | 0.84 | 0.38 | 0.42 | -0.82 | 0.78 | 0.79 | -0.71 | 0.63 | 0.66 | -0.93 | -0.98 | -0.19 | -0.16 | 0.08 | -0.21 | -0.15 | -0.14 | 0.98 | 0.98 |
